# The effects of intensified training on resting metabolic rate (RMR), body composition and performance in trained cyclists

**DOI:** 10.1371/journal.pone.0191644

**Published:** 2018-02-14

**Authors:** Amy L. Woods, Anthony J. Rice, Laura A. Garvican-Lewis, Alice M. Wallett, Bronwen Lundy, Margot A. Rogers, Marijke Welvaert, Shona Halson, Andrew McKune, Kevin G. Thompson

**Affiliations:** 1 Research Institute for Sport and Exercise, University of Canberra, Bruce ACT, Australia; 2 Department of Physiology, Australian Institute of Sport, Bruce ACT, Australia; 3 Mary Mackillop Institute for Health Research, Australian Catholic University, Melbourne, Victoria, Australia; 4 Department of Nutrition, Australian Institute of Sport, Bruce ACT, Australia; 5 Discipline of Biokinetics, Exercise and Leisure Sciences, School of Health Sciences, University of KwaZulu-Natal, Durban, South Africa; University of Rome, ITALY

## Abstract

**Background:**

Recent research has demonstrated decreases in resting metabolic rate (RMR), body composition and performance following a period of intensified training in elite athletes, however the underlying mechanisms of change remain unclear. Therefore, the aim of the present study was to investigate how an intensified training period, designed to elicit overreaching, affects RMR, body composition, and performance in trained endurance athletes, and to elucidate underlying mechanisms.

**Method:**

Thirteen (n = 13) trained male cyclists completed a six-week training program consisting of a “Baseline” week (100% of regular training load), a “Build” week (~120% of Baseline load), two “Loading” weeks (~140, 150% of Baseline load, respectively) and two “Recovery” weeks (~80% of Baseline load). Training comprised of a combination of laboratory based interval sessions and on-road cycling. RMR, body composition, energy intake, appetite, heart rate variability (HRV), cycling performance, biochemical markers and mood responses were assessed at multiple time points throughout the six-week period. Data were analysed using a linear mixed modeling approach.

**Results:**

The intensified training period elicited significant decreases in RMR (F_(5,123.36)_ = 12.0947, p = <0.001), body mass (F_(2,19.242)_ = 4.3362, p = 0.03), fat mass (F_(2,20.35)_ = 56.2494, p = <0.001) and HRV (F_(2,22.608)_ = 6.5212, p = 0.005); all of which improved following a period of recovery. A state of overreaching was induced, as identified by a reduction in anaerobic performance (F_(5,121.87)_ = 8.2622, p = <0.001), aerobic performance (F_(5,118.26)_ = 2.766, p = 0.02) and increase in total mood disturbance (F_(5, 110.61)_ = 8.1159, p = <0.001).

**Conclusion:**

Intensified training periods elicit greater energy demands in trained cyclists, which, if not sufficiently compensated with increased dietary intake, appears to provoke a cascade of metabolic, hormonal and neural responses in an attempt to restore homeostasis and conserve energy. The proactive monitoring of energy intake, power output, mood state, body mass and HRV during intensified training periods may alleviate fatigue and attenuate the observed decrease in RMR, providing more optimal conditions for a positive training adaptation.

## Introduction

Periods of intensified training are deliberately programmed to foster physiological and psychological adaptations to potentially improve physical performance. It is critical, however, to ensure that a balance between training-induced fatigue and sufficient recovery exists, in order to prevent excessive load on the athlete, and minimize the risk of maladaptation to training, illness or injury. Training-related distress can be viewed along a continuum from acute fatigue to overtraining. Short-term periods of intensified training may result in performance decrements associated with acute fatigue, which, upon appropriate recovery, can elicit an adaptive response to improve performance. This state is classically termed ‘overreaching’ or ‘functional overreaching (FOR)’, and is often employed during training camp-situations, with symptoms resolved within several days to weeks. It is important to distinguish between acute fatigue and FOR, however, since the super-compensation effect is reported to be smaller in FOR than acute fatigue [1], and FOR can elicit a greater risk for training maladaptation [2].

Progression of symptoms, and continued imbalance between training and recovery may lead to a more extreme state of severe overreaching, or ‘non-functional overreaching (NFOR)’. NFOR is typically characterized by the inability to sustain effort through intense exercise, diminished performance with maintenance or progression of the training load, and excessive fatigue both at rest and during exercise. Athletes may also present with mood disturbances, psychosocial stress, nutritional and sleep disturbances, and illness, with recovery from NFOR taking several weeks to months [3–5]. Whilst the progression from NFOR to overtraining is considered the most debilitating, the distinction between the two states is complex, since the “clinical features [of overtraining] are non-specific, anecdotal and numerous [5]”, and vary from one individual to another. Consequent long-term performance decrements from overtraining may require several months to years for recovery [3, 4, 6, 7], and should be prevented, wherever possible.

Athletic responses to intensified training periods have been studied extensively [2, 5, 8–11], but there remains no single diagnostic marker to distinguish between acute fatigue, overreaching and overtraining. Much of the applied literature has largely centered on declines in psychological and perceptual measures [1], as well as external measures such as power output, to aid in assessing the severity of an athletes’ condition. The continuum toward overtraining has also been proposed to involve disturbances at the hypothalamic-pituitary level, which may manifest in a reduced hormonal response to exercise [12–14]. In particular, previous studies suggest a disturbance in mood state, impaired race times and decreased power output may occur in athletes suffering from overreaching or overtraining [14–19].

Previous research from the present group suggests that changes in resting metabolic rate (RMR), body composition and energy intake may also be plausible indicators of training distress [20]. RMR is the minimum energy the body requires to perform its basic functions, and is principally dependent on lean mass [21]. In an applied setting, RMR can be used as an indicator of energy availability (EA); defined as the energy remaining for metabolic processes once the energy cost of exercise has been subtracted from dietary intake [22]. Sufficient energy is critical for training consistency, particularly during intensified periods, since prolonged energy restriction can lead to impaired physiological function and increased risk of fatigue, illness and injury, as well as maladaptation to the prescribed training [23]. Significant reductions in RMR, body mass and fat mass have been observed in elite rowers completing four weeks of intensified training at sea level [20], however increases and decreases in RMR have also been observed during altitude training camps in elite and highly-trained athletes, contingent on training volume and dietary practices [24, 25]. Energy homeostasis is centrally regulated, and RMR is closely linked to appetite and energy intake [26, 27]. Therefore, when energy intake is insufficient to support an intensified training load, athletes are more likely to suffer suboptimal EA and a lower RMR. Under such conditions, time trial performance has been demonstrated to decrease in an elite rowing cohort where a state of substantial fatigue and possible overreaching may have occurred [20]. It is plausible that a relationship exists between RMR, energy intake, EA and training load tolerance in endurance athletes, but further data is required to support this premise and to determine the underlying mechanisms involved. Further examination of this relationship is currently being undertaken by a subgroup of our authors.

The aim of the present study was to investigate how an intensified training period, designed to elicit overreaching, affects RMR, body composition and performance in trained endurance athletes, and to elucidate underlying mechanisms. We hypothesised that intensified training would elicit an increased energy demand, leading to reductions in RMR, body composition and performance.

## Method

### Study design

**Fig 1:**
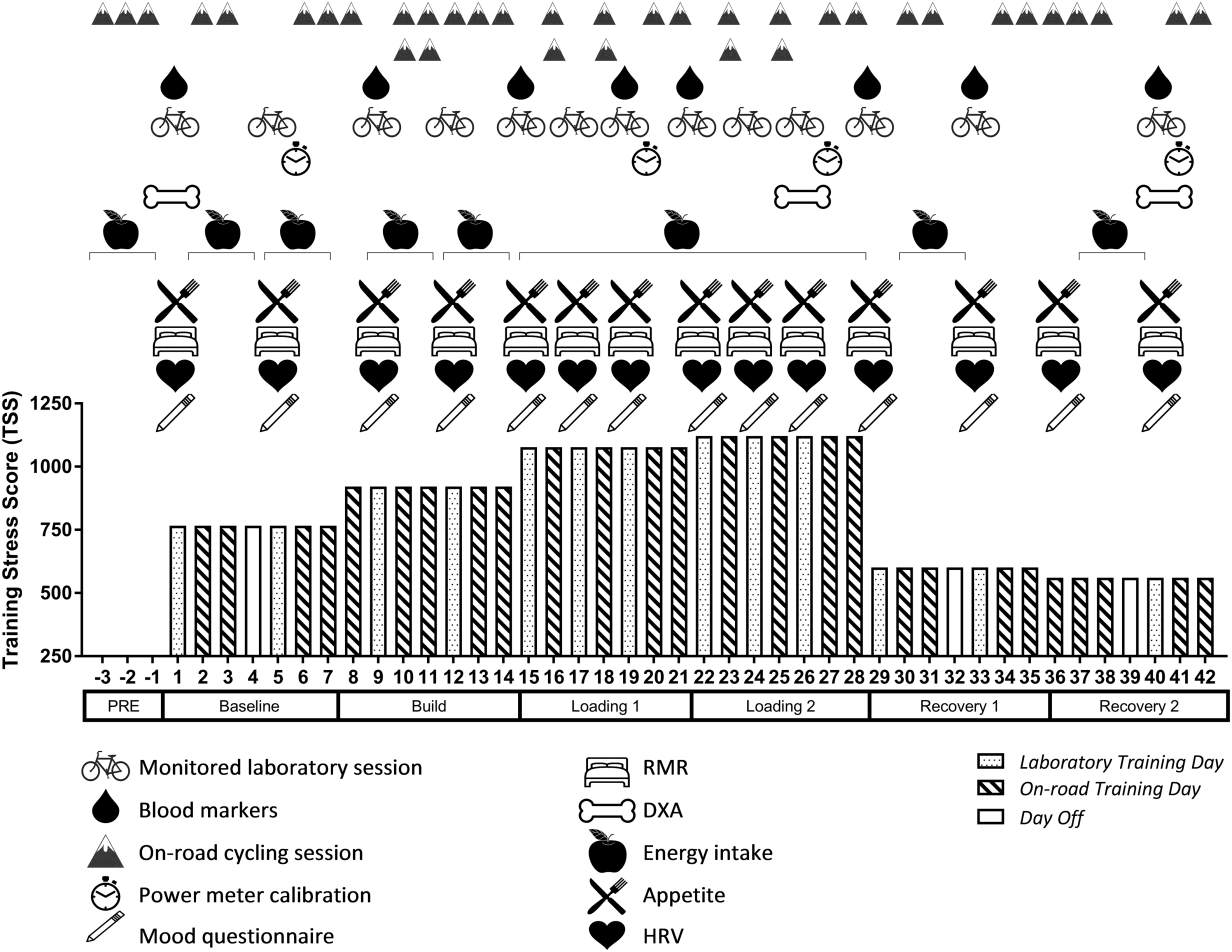
Study design showing the training load undertaken in TSS points per week, the training sessions prescribed, and the corresponding physiological and perceptual measures taken. Key: Monitored Laboratory Session—consisting of the standardised warm up, assessment of cycling performance, and HIIT training session; Biochemical Markers—PRE and POST warm up blood samples for leptin and fT3; On-road Cycling Session– 1) long duration, aerobic-based session and 2) hill repeats; Power Meter Calibration—timed repetition of a known distance and elevation; RMR—Resting Metabolic Rate; Body Composition—from Dual-Energy X-Ray Densitometry (DXA); Energy Intake—from 3-day food diaries; Appetite—visual analogue scales to determine appetite; Mood Questionnaire—consisting of the Multicomponent Training Distress Scale, Recovery Stress Questionnaire for Athletes (RESTQ-52 Sport); HRV—Heart Rate Variability. The spotted bars indicate a laboratory-training day; the striped bars indicate an on-road cycling training day; the white bars indicate a rest day.

Thirteen trained male cyclists completed a six-week training program designed to achieve an overreached state followed by a recovery period. The study was approved by both the Australian Institute of Sport Human Ethics Committee and University of Canberra Human Research Ethics Committee. All participants provided written informed consent prior to involvement. Training was individualized based on each participant’s training history. Training consisted of a combination of monitored, laboratory-based high-intensity interval sessions, and on-road cycling. RMR, body composition, energy intake, appetite, cycling performance, heart rate variability (HRV), biochemical markers and mood responses were assessed at multiple time points throughout the six-week period (Fig 1)

### Participants

Fourteen male cyclists were recruited from local cycling and triathlon clubs in Canberra, Australia between December 2015 and March 2016 for participation in the six-week program. One participant was unable to continue the training commitments after week 2. Characteristics of the 13 participants who completed the study were (mean ± SD, range): age 35 ± 8 years, 20–47 years; height 185 ± 7 cm, 175–195 cm; body mass 80.5 ± 7.3 kg, 66.0–94.5 kg; maximal oxygen uptake (V̇O_2max,_ relative) 61.1 ± 6.2 ml.min^-1^kg^-1^, 52.9–73.0 ml.min^-1^kg^-1^; maximal aerobic power (MAP, absolute) 378 ± 28 W, 333–425 W; V̇O_2max_ (absolute) 4.9 ± 0.2 L.min^-1^, 4.7–5.3 L.min^-1^; MAP (relative) 4.8 ± 0.6 W.kg^-1^, 3.7–5.5 W.kg^-1^. Participants had a consistent cycling training history (> 5 sessions.wk^-1^, > 10 h.wk^-1^, > 200 km.wk^-1^, > 4 years) and regularly competed in A and B-grade cycling races. Based on previous literature [28], the subjects were classified as Performance Level 3.

To determine statistically significant changes in RMR, a sample of n = 8 athletes would be required, based on a smallest worthwhile change in RMR of 8% [29], a within-subject SD of 4.3% [30], and Type I and Type II errors of 5% and 20% respectively. Due to the highly applied and demanding nature of the study, it was not possible to pair match an independent control group. We acknowledge this as a limitation to the study.

### Training load

The study period was six weeks in total, consisting of a “Baseline” week (100% of regular training load; monitored for the four weeks immediately prior to the study beginning), a “Build” week (~120% of Baseline load), two “Loading” weeks (~140 and 150% of Baseline load, respectively) and finally two “Recovery” weeks (~80% of Baseline load, see Fig 1. Weekly training was prescribed individually through online software (Training Peaks, Boulder, CO), based on Training Stress Score (TSS). TSS is a training load index similar to the heart-rate based TRIMP method; taking into account the duration and intensity of the activity using power output whereby 100 TSS points is equivalent to one hour of exercise at an individual’s functional threshold power [FTP, the power output at which 4 mmol.L^-1^ blood lactate (BLa) concentration was reached via the power-versus-lactate curve, or lactate threshold 2 [8, 31, 32]). Participant’s baseline TSS was calculated to reflect the average of their four weeks training prior to the study beginning. All sessions were monitored and adjusted where required to reach the target TSS each week.

### Preliminary testing

In the two weeks prior to the study beginning, participants completed an incremental cycling test to exhaustion using an electromagnetically braked cycle ergometer (Lode Excalibur Sport, Groningen, Netherlands) to assess V̇O_2max_ and MAP, as has been described previously [33–35]. Individual training zones and FTP were subsequently calculated based on power output, heart rate (HR) and BLa values obtained for each incremental stage using in-house software [Automatic Data Analysis for Progressive Tests (ADAPT) v6.7, Canberra, Australia].

### Resting metabolic rate

RMR was assessed on eleven mornings across the six-week period (Fig 1) using the criterion Douglas Bag method of indirect calorimetry, which has been described previously [30]. All athletes were overnight rested and fasted, and abstained from physical activity for at least eight hours prior to all measurements, which were each completed at the same time of day (± 1 h). Typical error (TE) for the Douglas Bag method of RMR measurement in our hands is 286.8 kJ, or 4.3% [90% confidence limits (CL): 3.1–7.2%] within days, and 455.3 kJ or 6.6% (90% CL: 4.8–11.1%) between days.

### Body composition

Body composition was assessed immediately following three of the RMR measurements (Baseline, end of Loading 2, end of Recovery 2; Fig 1) via Dual-Energy X-Ray Densitometry (Lunar iDXA; GE Healthcare Asia-Pacific). Each DXA scan provided an assessment of fat mass, lean mass and bone mineral content (BMC). Fat-free mass (FFM) was calculated as lean mass plus BMC. Radiation safety approval was provided by the Radiation Safety Committee at the John James Hospital, Canberra.

### Energy intake

Dietary intake was recorded either by paper diary record or iPhone application (Easy Diet Diary, Xyris Software Pty Ltd, Australia) for the three days immediately prior to each RMR measurement (Fig 1), and analysed for total energy intake and macronutrient consumption by an accredited practising dietitian using nutrient analysis software (FoodWorks Professional v7.0.3016, Xyris Software Pty Ltd, Australia).

### Appetite

Subjective feelings of appetite were assessed prior to breakfast following each RMR measurement via 1–10 Likert visual analogue scale (VAS, Fig 1), adapted from [36] (S1 Fig).

### Heart rate variability

HRV was assessed during the 25-minute rest period of each RMR measurement, for eleven measurements in total (Fig 1). Upon arrival to the laboratory, participants were fitted with a HR strap (Firstbeat Technologies Ltd, Jyväskylä, Finland). Upon resting supine for five minutes, a ten-minute recording was taken, which was divided into five minutes of rest followed by a five-minute measurement of inter-beat intervals. The inter-beat intervals were analysed using open source analysis software [Kubios HRV Software version 2.0; Biosignal Analysis and Medical Imaging Group, Department of Physics, University of Kuopio, Finland [37]] for time-domain analysis of the mean square root differences of the standard deviation (RMSSD) and its log (LnRMSSD).

### Monitored laboratory sessions and cycling performance

Following an initial familiarization on Day 1, 12 monitored laboratory sessions were performed across the six-week period (Fig 1), inclusive of a standardised warm-up, assessment of cycling performance, and a high-intensity interval training (HIIT) session (option 1, 2 or 3) with varied work-rest ratios (Table 1). Participants were blinded to external feedback cues, and instructed to complete all efforts with maximal exertion. Peak power output was recorded immediately following the 15 s sprint. The power output data for the 5 s sprints were discarded due to concerns over the precision of the ergometer’s power output measurement and reliability of the participants’ effort. Mean power output, time to completion and Rating of Perceived Exertion (RPE, 6–20 Borg Scale [38]) were recorded immediately following the 4000 m TT, with BLa measured from capillary sample one minute later. HR was blinded, but monitored continuously throughout (Firstbeat Technologies Ltd, Jyväskylä, Finland). All sessions were performed using calibrated cycle ergometers (Wattbike Pro, Wattbike, Nottingham, UK). Each participant was assigned to the same individual bike for the entire study to ensure measurement error was minimised. Laboratory sessions were completed at the same time of day (± 1 h), with a minimum of two days between each session.

**Table 1:** Outline of the monitored laboratory sessions and assessment of cycling performance.

**A) Standardised Warm up**			
	**Elapsed Time**	**Description**	
Warm Up	00:00.00–06:00.00	6 minutes @ 60% MAP	
	06:00.00–12:00.00	6 minutes @ 70% MAP	
	12:00.00–15:00.00	3 minutes @ 80% MAP	
	15:00.00–16:00.00	1 minute @ 90% MAP	
	16:00.00–18:00.00	2 minutes @ 70% MAP	
	18:00.00–19:00.00	1 minute easy	
Warm up Effort 1	19:00.00–19:05.00	5 s warm up sprint @ 80% RPE	
	19:05.00–20:00.00	55 s recovery	
Warm up Effort 2	20:00.00–20:05.00	5 s warm up sprint @ 90% RPE	
	20:05.00–23:00.00	2 minutes 55 s recovery	
**B) Cycling Performance**			
Effort 1	23:00.00–23:05.00	5 s maximal sprint	
	23:05.00–24:00.00	55 s recovery	
Effort 2	24:00.00–24:05.00	5 s maximal sprint	
	24:05.00–26:00.00	1 minute 55 s recovery	
Effort 3	26:00.00–26:15.00	**15 s maximal sprint (performance test)**	
	26:15.00–32:00.00	5 minutes 45 s recovery	
Effort 4		**4000 m maximal TT (performance test)**	
	00:00.00–06:00.00	6 minutes recovery	
**C) HIIT Session**			
**Option**	**Effort**	**Recovery between sets**	**Repetition**
**1**	4 x (15 s on/45 s off)	3 minutes, 45 s	Repeat x 3
	12 x (5 s on/15 s off)	3 minutes, 15 s	
	6 x (10 s on/30 s off)	3 minutes	
**2**	6 x (10 s on/10 s off)	3 minutes, 10 s	Repeat x 3
	4 x (15 s on/30 s off)	3 minutes	
	10 x (5 s on/15 s off)	2 minutes, 25 s	
	3 x (20 s on/40 s off)	3 minutes	
**3**	4 x (20 s on/40 s off)	3 minutes	Repeat x 3
	4 x (15 s on/45 s off)	3 minutes, 25 s	
	6 x (10 s on/10 s off)	2 minutes, 10 s	
	5 x (5 s on/15 s off)	3 minutes	

A) Standardised warm-up, B) assessment of cycling performance, and C) one of three high-intensity interval training (HIIT) session options.

### On-road cycling

On alternate days to the laboratory sessions (Fig 1), participants completed two on-road rides in their own time, with a minimum of five hours between each: 1) long duration, aerobic-based session and 2) a series of hill repeats at FTP in order to induce fatigue. Training zones were based on V̇O_2max_ test results, as previously described. Power output data (Stages left arm crank: Colorado, USA; Garmin Vector: Kansas City, USA; SRM Training System: Jülich, Germany) and HR data (Garmin: Kansas City, USA) for each cyclist were uploaded to Training Peaks upon completion. Each individual’s power meter recording was standardised during 4 x on-road trials using a known distance and elevation (2.8 km, 812 m; Black Mountain, Canberra, Australia, Fig 1). For each trial, the total mass of the rider and bike were recorded, followed by the time to complete one repetition of the known course. Predicted power output was then calculated using a validated regression based on speed, mass and time to complete [39]. The difference between the predicted power and the device-recorded power was then compared to ensure consistency in the power meter recordings across time. Power comparison data was not utilised for any other purpose than assessing for drift in the predicted-actual power relationship.

### Biochemical markers (PRE-POST ergometer)

On eight occasions during the monitored laboratory sessions (Fig 1), venous blood samples (1 x 8.5 ml serum separator tube) were obtained via venipuncture from an antecubital forearm vein by qualified phlebotomists. Samples were taken before and after a standardised exercise, i.e. at rest (PRE) and immediately following (POST) the standardised warm-up, in an attempt to mitigate the large variability in the assessment of leptin and free thyroid hormone (triiodothyronine, fT3). External analysis was conducted via immunoassay (Cardinal Bioresearch, Queensland, Australia): Leptin was assayed using a DuoSet^®^ ELISA kit (R&D Systems Inc, Minneapolis, USA), and fT3 on the Siemens ADVIA Centaur automated instrument (Siemens Healthcare Diagnostics Ltd, NY, USA) as per manufacturer’s recommendations. Raw data were then assessed as the percentage change between PRE and POST, per session.

### Mood questionnaires

Two mood questionnaires, the Multicomponent Training Distress Scale (MTDS) [40] and the Recovery Stress Questionnaire for Athletes (RESTQ-52 Sport) [41], were administered at the same time of day (between 0900 and 1100) according to authors’ instructions on fourteen occasions throughout the six-week period to assess training-related mood disturbance (Fig 1).

### Data analysis

The present study design involved repeated measures of multiple variables at specific time points, and a number of proposed inter-variable relationships. A multivariate structural equation model (SEM) was initially employed, however the complexity of the study design and irregularity of measurement points meant that the SEM did not achieve convergence. A linear mixed modelling approach was thus utilised, with independent regressions defined based on the previously predicted SEM relationships. These models allowed us to investigate the time evolution of the dependent variables, associations with other variables (covariates), as well as modelling the substantial amount of heterogeneity amongst subjects and varying baseline levels. All analyses were carried out using the lme4 package [39] in R [40]. The technical specifications of the models are: 1) inclusion of a random intercept for participants, 2) Restricted Maximum Likelihood (REML) estimation, and 3) significance testing of the fixed effects using Type II F tests with Kenward-Roger degrees of freedom approximation. The selection of independent variables included in the models was initially based on a visual assessment of descriptive plots assessing the relationship between the dependent and independent variables. Only those variables that presented the strongest relationship with the dependent variable were included as fixed effects in the linear mixed models. This procedure was adopted to avoid issues with multi-collinearity (e.g. including similar variables that highly correlate) and to avoid over-testing, thus minimizing inflated Type I errors. After fitting an initial full model, a backward model selection procedure was carried out to remove non-significant variables, which helped in the interpretation of the models. Each of the models included evolution over time as a fixed effect (i.e. Training Block), regardless of whether there were any visible changes over time in the visual assessment.

Linear mixed model data are available in Supporting Information Tables 1 to 7 (S1–S7 Tables), and presented as the F-statistic and p-value, with significance set at 0.05. 95% bootstrapping confidence intervals (95% CI) are also presented for those effects that reached statistical significance. Raw data are available in Supporting Information Tables 8 to 18 (S8–S18 Tables), and presented as individual values for each time point, and group mean ± SD.

## Results

### Training load

**Fig 2:**
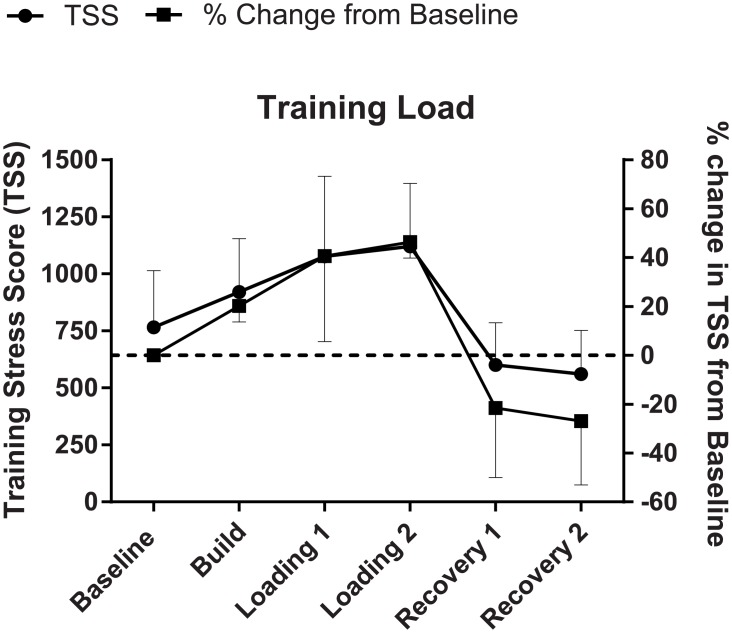
Training load. Data are presented as (mean ± SD) for the actual TSS achieved by the participants on the left y-axis, and the corresponding Δ% in TSS from Baseline on the right y-axis.

Group TSS scores (mean ± SD) for each week throughout the training period were: Baseline = 766 ± 249, Build = 921 ± 234, Loading 1 = 1077 ± 351, Loading 2 = 1121 ± 277, Recovery 1 = 601 ± 186 and Recovery 2 = 560 ± 192; which corresponded to percentage loadings (compared to Baseline) of 120%, 141%, 147%, 79% and 73%, respectively (Fig 2).

### Linear mixed models

#### Resting metabolic rate

Absolute RMR and relative RMR were significantly related to the training block (p < 0.05; Table 2), with reductions observed from Baseline to Loading 2, before returning toward Baseline levels in Recovery 2.

**Table 2:** Linear mixed model data for the resting metabolic rate (RMR) model.

	Training Block	Training Stress Score (TSS)	Total energy intake (mJ.day^-1^)	HRV (LnRMSSD)
**Absolute RMR (kJ.day**^**-1**^**)**	F_(5, 123.36)_ = 12.0947, p = < 0.001***	F_(1, 127.4)_ = 5.3509, p = 0.02** (-)	F_(1, 107.06)_ = 0.7349, p = 0.39	F_(1, 105.45)_ = 0.0035, p = 0.95
**Relative RMR (kJ.kg.FFM**^**-1**^**)**	F_(2, 23.93)_ = 6.824, p = < 0.001**	F_(1, 28.786)_ = 5.4759, p = 0.03* (-)	F_(1, 30.824)_ = 6.2472, p = 0.02* (+)	-

FFM = fat-free mass; HRV = heart rate variability

Data are presented as the F-statistic and p-value, and a +/- symbol to denote a positive or negative linear association over time, where relevant. Where a significant linear relationship is observed,

* denotes p < 0.05,

** denotes p < 0.01,

*** denotes p < 0.001.

#### Body composition

Body mass significantly decreased from Baseline to Loading 2 [95% CI = -1.395; -0.162], and remained low thereafter [95% CI = -1.439; -0.123] (S1 Table).

#### Energy intake

Total energy intake, fat and protein were not significantly related to the training block (p > 0.05). However, CHO consumption increased from Baseline to Loading 2 [95% CI = 21.011; 132.436], and returned toward baseline levels by Recovery 2 [95% CI = -97.030; 50.099] (S2 Table).

#### Appetite

Pre-breakfast sensations of ‘how much the participants felt they could eat’ were decreased between Baseline and Loading 2 [95% CI = -1.816; -0.595], and returned towards baseline levels by Recovery 2 [95% CI = -1.013; 0.372] (S3 Table).

#### Biochemical markers

Leptin and fT3 were not significantly related to the training block, TSS or absolute RMR (p > 0.05; S4 Table).

#### Heart rate variability

A significant positive association was observed between HRV and fT3 levels [F_(1, 22.122)_ = 4.5974, p = 0.04]. An interaction effect on HRV was also observed between relative RMR and the training block [F_(2, 22.608)_ = 6.5212, p = 0.005], whereby the higher the relative RMR, the higher the HRV [95% CI = -0.171; 4.385] (S5 Table).

#### Cycling performance

Peak power output for the 15 s sprint decreased in 12 participants by the end of Loading 2, and returned toward baseline levels by Recovery 2 (S6 Table). Mean power output for the 4000 m TT decreased in 9 participants by the end of Loading 2, and returned toward baseline levels by Recovery 2 [95% CI: -2.294; 93.578]. An interaction effect on mean power output was also observed between TSS and RESTQ-52 Total Stress [F_(5, 118.51)_ = 2.4486, p = 0.04], whereby the higher the stress and TSS, the lower the power output [95% CI = -0.097; -0.032]. Peak HR and RPE during the 4000 m TT decreased from Baseline to Loading 2 [HR: 95% CI = -6.555; -0.608; RPE: -1.545; -0.097], and returned toward baseline levels by Recovery 2 [HR: 95% CI = -2.292; 4.583; RPE: -0.613; 1.176] (S6 Table).

#### Mood questionnaires

Increases in both MTDS Total Mood Disturbance (TMD) and RESTQ-52 Total Stress were significantly associated with the training block. Responses increased from Baseline to Loading 2 [TMD: 95% CI = 1.701; 4.562; RESTQ: 95% CI = 0.171; 0.929], and returned toward baseline levels by Recovery 2 [TMD: 95% CI = -2.091; 1.436; RESTQ: 95% CI = -0.334; 0.519] (S7 Table).

### Time course of change

**Fig 3:**
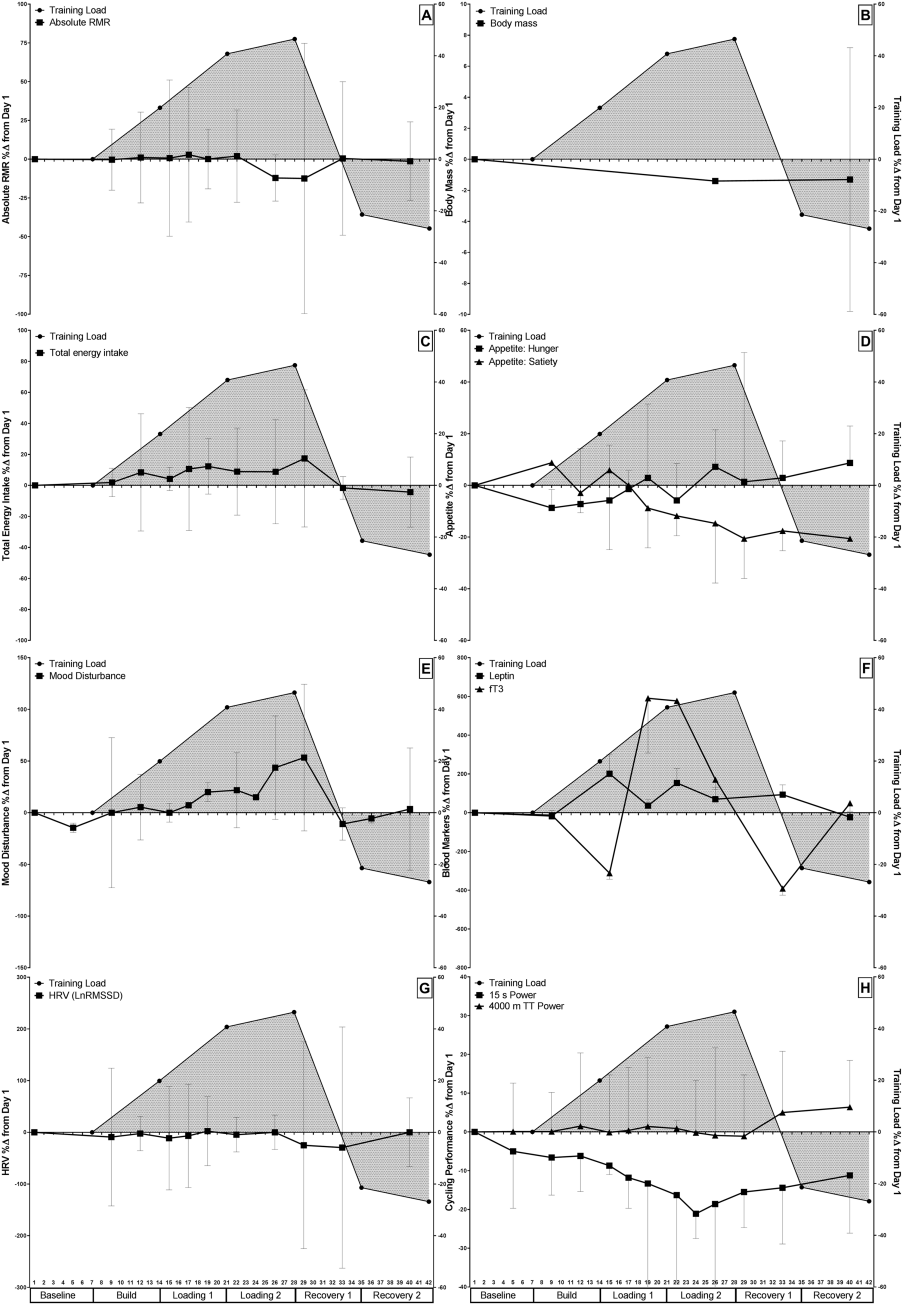
Percentage change in measured variables from baseline in relation to training load across the study duration for A) RMR, B) Body mass, C) Total energy intake, D) Appetite, E) Mood disturbance, F) Biochemical markers leptin and fT3, G) Heart rate variability (LnRMSSD), and H) Cycling performance. The left y-axis depicts Δ% in each of the measured variables, with Δ% in training load on the right y-axis, shaded beneath the curve.

Raw data comparisons for each variable across the study period as a percentage change from Day 1 are presented in Fig 3.

## Discussion

### Main findings

The present period of intensified training elicited a state of overreaching in trained male cyclists, and significantly decreased both absolute and relative RMR, body mass, fat mass and HRV, with concomitant increases in mood disturbance, and declines in anaerobic performance, aerobic performance and associated peak HR; all of which improved following a period of recovery. It is likely that the increased energetic demands of training, coupled with insufficient energy intake, are contributing factors to these results; supporting recent evidence from elite rowers that significant decreases in RMR, body composition and performance can occur with heavy training loads when energy intake does not keep up with a greater energy output [20, 24]. The present data do not support the notion that RMR might be a useful marker to monitor training adaptation. Instead, we advocate the proactive monitoring of validated markers of training distress, including subjective wellness, energy intake, power output, body mass and HRV to attenuate fatigue and the potential for a decline in RMR; promoting athlete health, wellbeing and training ability.

### RMR, energy availability and intensified training

Relative RMR decreased in the present participants from ~122 to 107 kJ.kg.FFM.day^-1^ (~29 to 26 cal.kg.FFM.day^-1^) at the end of the intensified training weeks, supporting a likely decrease in EA as a result of the training load, and an increased risk of physiological dysfunction. This notion is supported by the negative linear relationship between both absolute and relative RMR and training load, whereby the greater the training load, the lower the RMR. Much of the previous literature on lowered EA responses to exercise has focused on female athletes who have also demonstrated symptoms of the formerly-known ‘Female Athlete Triad’ including menstrual dysfunction, disordered eating and impaired bone health [22, 42–44]. The novel data in the present study demonstrate that male athletes can also suffer a low relative RMR and potentially a low EA, which is supported by previous data from our group [20, 24]. Our data also agrees with recent work advocating that male athletes may be susceptible to similar adverse health effects associated with energy restriction as females [45], and confirms the recent notion of ‘Relative Energy Deficiency in Sport’ as a condition applicable to all athletes [23]. These data suggest that there is potential benefit in monitoring RMR within the daily training environment. However, we acknowledge that the measurement of RMR requires specialist equipment and so might only be undertaken where a more extensive investigation of an athlete’s training maladaptation is warranted. By understanding an athlete’s ‘normal’ RMR, and their energy demands at rest, practitioners would be better able to recognize the individual threshold at which values begin to deviate in conjunction with training load. Such knowledge, along with the proactive monitoring of energy intake and body mass, may help to ensure that athletes do not suffer energy restriction from a mismatch between energy intake and output during heavy training, and promote a more optimal EA. Importantly, by maintaining a more optimal RMR and EA, athletes are more likely to have sufficient energy for training as well as crucial physiological functions including bone health, growth and repair, cardiovascular, gastrointestinal and haematological function; ultimately promoting athletic performance [23].

### Evidence that overreaching occurred

#### Performance decline

Training distress in the present cohort was demonstrated by small but significant reductions in both aerobic (4000 m TT, -1.1%) and anaerobic (15 s, -21.1%) performance by the end of the loading weeks, coupled with a decrease in peak HR values. These data agree with other studies reporting performance decrements [16–18, 46–48], however a handful of studies have observed either no decline [49], or even improvements in TT performance in highly-trained and elite cyclists following an overload training period [50, 51]. Such discrepancies may relate to the degree of overload imposed and the training status of the participants; with more highly trained participants being more resilient to increased training volume and intensity. It must also be acknowledged that whilst fatigue is more than likely the driving factor for the observed decreases in peak HR values, a simple explanation could be that these lowered values are directly related to the lowered peak power output from the performance trials. Further investigation is required to ascertain the mechanisms for such changes.

Interestingly, as shown in Fig 3H, the decline in 15 s peak power output occurred prior to the decline in mean power output for the 4000 m TT, and was of a greater magnitude. As we did not indirectly measure muscle activation by integrated electromyography activity nor undertake specific measurements examining changes in neuromuscular function we can only speculate if this decline in anaerobic performance was due to i) peripheral fatigue ii) central fatigue, or iii) a combination of both. We can also only speculate whether participants made a conscious decision to increasingly reduce their effort on this task during the intensified training weeks to conserve energy/themselves for the 4000 m TT. That said, the earlier decline in 15 s peak power output data in the current investigation might indicate that predominantly anaerobic efforts are a more sensitive marker of training distress than a short-term endurance effort such as a 4000 m TT. Rietjens and Kuipers [52] have proposed that a decline in reaction time to a finger pre-cuing test was strongly suggestive of central fatigue preceding peripheral fatigue. In that study, the training load was significantly increased from baseline for two weeks, however no changes in hormonal responses, body composition or physiological assessments (power output, HR, BLa) were observed, which may imply that their participants did not suffer sufficient training-induced distress to stimulate both central and peripheral fatigue. The present data suggests that regular monitoring of power output during both aerobic and anaerobic efforts may aid in the assessment of training-related distress within the daily training environment.

#### Mood disturbance

Present perceptions of stress and recovery were consistent with increased training volume, and participants demonstrated a worsened mood state through the loading weeks. The perceptual responses provide additional confirmation that the training prescription was sufficient to induce a state of overreaching. These findings are not unique, but rather support recent research in elite rowers from the present group [20] and others [1, 16, 17, 19, 46, 49, 53–55]. Interestingly, RPE for the 4000m TT decreased through the loading weeks, which may suggest that, even though participants were instructed to complete a maximal effort, they were unable or less motivated to do so as a result of their fatigue state. RPE is also well-correlated with HR during steady-state and high-intensity cycle training [8], and so the reduction in RPE might be related to the lowered maximal HR values observed. De Koning et al [56] has postulated that RPE in a closed-loop trial is dependent on the magnitude and rate of homeostatic disturbance, as well as the knowledge of duration or distance remaining. It is plausible that participants experienced a greater homeostatic disturbance earlier in the 4000m TT during the loading weeks and subconsciously adjusted their pacing, which led to a subtle reduction in power output, heart rate and RPE. However, it should also be noted that post-exercise RPE scores are also prone to variability as physiological feedback is diminishing as soon as exercise is terminated and so there can be significant measurement error [57].

A number of statistical associations were also observed between mood disturbances, perceived recovery and HRV, providing a potential link between training load, mood responses and autonomic nervous system activity. Being some of the earliest to change, these data further reinforce the importance of subjective assessments (like RPE) as some of the easiest and more reliable markers to monitor athlete wellbeing and training adaptation, particularly within ecological situations such as training camps [1, 46, 58, 59].

### Possible mechanisms for the observed changes in RMR

#### Body composition

An individual’s FFM is the greatest determinant of RMR, thus a greater amount of FFM results in a higher energy requirement due to a greater proportion of metabolically active tissue [60, 61]. Previous research has largely demonstrated increases in RMR following exercise, possibly related to increases in FFM [62, 63], increased metabolic demand in response to exercise-induced muscle damage [64–68], and excess post-exercise O_2_ consumption (EPOC), which may elevate energy expenditure for up to 24 hours following training [69, 70]. In the present study, we suggest that the small but significant changes in FFM between Baseline and the end of the loading period (-1.3%) are likely to have only partially affected RMR to the extent observed (-12.1%). In addition, participants would have demonstrated some muscle damage and EPOC during the intensified training periods but they did not demonstrate an increase in RMR. A possible explanation for these contradictory findings might be due to the timing of training on the day prior to the RMR measurement, however, in our study, training activity was standardised, and so we are confident our results were not affected in this way. We propose that the decreases in both absolute and relative RMR were due to a compensatory response to the intensified training load or insufficient energy intake, or both.

In addition, participants’ body mass and fat mass decreased by the end of the loading weeks, suggesting an energy imbalance. Taken together with the finding of a reduction in both absolute and relative RMR, these data support earlier studies which have suggested energy conservation under intensified training circumstances [20, 71]. One contrasting study found no change in body mass or fat mass in competitive cyclists undertaking two weeks of intensified training [18], however the study estimated body composition from skinfold measurements, which typically have lower test-retest reliability than the DXA method used in the present study, and so might account for the disparity in the findings. Nonetheless, our findings emphasize the critical nature of maintaining energy intake, independent of feelings of appetite (which might be relatively insensitive), in order to maintain body mass and RMR; each of which are strongly linked [72]. This notion is particularly important for athletes who cannot afford to lose lean mass, risking a decline in performance from a decrease in muscular strength and power capabilities.

#### Energy intake and appetite

Supplementary CHO ingestion throughout a training cycle has been reported to assist in alleviating the symptoms of overreaching [73, 74], and may mitigate the stress hormone response to exercise [75]. If total energy intake is insufficient, however, acute ingestion of CHO immediately before and after a training session may not provide an attenuation of fatigue-induced decreases in maximal power output or immunological disturbance [76]. The present cohort attempted to increase their CHO intake by the end of the loading weeks; however such compensation appears not to have been sufficient to attenuate a reduction in RMR or fatigue. It is plausible that changes in participants’ appetite responses were delayed in relation to the changes in energy output, and so an energy imbalance occurred. However, we acknowledge that individual appetite responses were highly variable, and so these findings must be interpreted with caution.

Leptin is a hormone secreted by the adipose tissue, and is reported to regulate neuroendocrine function, appetite perception and energy homeostasis through a series of complex interactions within the hypothalamus, the mesolimbic dopamine system and hindbrain [77–81]. High leptin levels are associated with increased satiety and energy expenditure, whilst low leptin levels, as seen in the present cohort, are consistent with low levels of body fat and chronic energy restriction [77, 81–85]. In particular, leptin has been suggested as a marker of training stress in male rowers [86], and is widely reported to decrease following heavy training periods [71, 87, 88]. In contrast to previous research, leptin levels in the present study tended to increase through the loading weeks, indicating greater satiety; however the responses were highly variable between individuals and so not statistically significant. Pre-breakfast perceptions of ‘how much the participants felt they could eat’ were lower in the loading weeks, further supporting an increase in satiety or decrease in hunger. Anecdotal reports from athletes within the Australian Institute of Sport cite a loss of appetite with heavy training, but these reflections, and our data, are not consistent with the literature. Another explanation of our findings might relate to dietary intake. In overweight and obese populations, overfeeding is reported to increase circulating levels of leptin [81]. More applicable to the present context, perhaps, is that leptin levels are highly correlated with carbohydrate intake [89], and can be influenced by circulating insulin and pro-inflammatory cytokines such as tumor necrosis factor and interleukin-6 [81], so it is possible that the observed trend of increased carbohydrate intake during intensified training had some effect. Perhaps another confounding factor in the observed leptin response was the timing of the blood sampling, which was undertaken prior to and immediately following a physical activity task, and may have been influenced by participants’ acute energy intake (such as glucose-rich sweets) prior to the blood sampling, as well as their feeding across the day. Despite this, the present data suggest that, in a practical sense, it is crucial for athletes to maintain sufficient energy intake to support their training load. It is possible that athletes should be instructed to eat in relation to the training undertaken, rather than appetite, to fuel optimal performance and recovery.

#### Thyroid hormone

Free triiodothyronine (fT3) has been proposed as a key regulator of metabolic rate and overall energy expenditure by modulating a number of regulatory pathways in skeletal muscle and other tissues [90–92]. Increases in circulating thyroid hormones are broadly associated with an increase in RMR, with the opposite trend occurring in response to lowered hormone levels [89]. Total T3 tended to decrease in response to chronic energy restriction and high-energy expenditure in a military setting [93]; and in females, T3 is lower in association with an increased severity of exercise-associated menstrual disturbances, reflective of energy conservation [85]. In the present study, the percentage change in fT3 demonstrated varied responses throughout the loading and recovery weeks, which did not result in statistical significance. Nonetheless, the substantial changes illustrated in Fig 3F might indicate an altered thyroid and hypothalamic–pituitary–thyroid (HPT)-axis activity as a result of the intervention, which may have practical implications for energy production and thermogenesis, nutrient metabolism, and the regular functioning of the cardiovascular system [94]. We were unable to measure these axes directly, however, and so this notion remains speculative and requires further investigation.

#### Heart rate variability

The observed reduction in LnRMSSD might be attributed to accumulated fatigue as a result of the training load, and may reflect the decreased ability of the ANS to respond to exercise training, stress and illness [95]. Reductions in LnRMSSD may further indicate parasympathetic hyperactivity (or saturation) and reduced sympathetic tone [96] if accompanied by increases in inter-beat intervals [97], which has been reported in response to periods of intensive training in elite and well-trained endurance athletes [97–100]. We propose that alterations in ANS activity might have influenced metabolic activity, as evidenced by the similar pattern of RMR and HRV responses, and the statistical association between fT3 and HRV. Fig 3 illustrates a decrease in RMR immediately prior to a decrease in HRV, so it is possible that an increase in parasympathetic activity, with ensuing reduction in sympathetic activity, may influence (or be influenced by) changes in RMR. Further research is needed to fully understand this potential association.

### Limitations

The present investigation was applied in nature, and whilst scientific rigour was paramount, there remain some limitations that must be acknowledged. Firstly, we acknowledge that our findings need to be interpreted with caution given that individuals, when training intensively, can exhibit highly variable responses, and also the statistically significant changes lay close to both the technical error of measurement and normal day-to-day variability. The study design consisted of multiple measurements across a number of time points, which resulted in difficulty in applying a statistical model; the power of which would have been improved with both a greater number of participants, as well as simultaneous measurements. The combination of biological and measurement error further adds complexity, and as such we have focused on the broad trends observed between variables. We also acknowledge the lack of an independent pair-matched control group, however the difficulty in retaining participants for the course of the six weeks meant it was not possible to recruit a separate cohort for comparison. Whilst this means that it is difficult to conclude with certainty that the changes observed are truly due to the training intervention applied, we are confident that by monitoring the participants for four weeks prior to the study beginning, we were able to gauge an accurate representation of their routine training. We are thus confident that the physiological changes observed during the study period can indeed be attributed to the increased training load. We also acknowledge the possibility that some of the unexpected responses to intensive training may be due to the trained status of our participants, who, given their routine volume of training might have been better able to adapt to the ‘predictable’ stress of the training imposed. As such, a number of different central responses might have been produced which we were not able to predict and subsequently assess. Finally, we recognize that the participants were free-living, trained cyclists, but not elite athletes. As such, they were subject to stressors outside of our control including work and study commitments, family duties, and lifestyle factors which may have added to the imposed training load.

### Practical application

The present data suggest that during periods of intensified training, practitioners should employ a series of monitoring tools—early, and often—to avoid detrimental levels of training-related distress and ensure sufficient energy intake to support the greater energetic demands. In the daily training environment, athletes should specifically be encouraged to increase their energy intake in relation to training load, rather than appetite, to support a more optimal EA. The proactive monitoring of subjective wellness, energy intake, power output, body mass and HRV during intensified training may further support athlete health, wellbeing and training ability before a detrimental decline in RMR, and likely EA, becomes apparent. Importantly, a more optimal RMR and EA will, in turn, ensure sufficient energy is available for training, recovery and adaptation, and ultimately, athletic performance.

## Conclusion

Athletes often undertake periods of intensified training in order to improve performance following a period of recovery. The present study demonstrates, however that exercising with an increased training load, without sufficient energy intake, can risk significant reductions in both absolute and relative RMR, body mass, HRV and performance, and increased mood disturbance. Such physiological disturbance and maladaptation to training may be problematic in athletes who cannot afford to lose mass, or those undertaking intense training prior to competition. We propose that a cascade of changes in metabolic, neural and hormonal mechanisms results from the body’s attempt to conserve energy and maintain homeostasis when energy demands are increased. The proactive monitoring of subjective wellness, energy intake, power output, body mass and HRV during intensified training periods may alleviate fatigue and attenuate any decreases in RMR, and subsequently provide more optimal conditions for a positive training adaptation.

## Supporting information

S1 Fig Subjective feelings of appetite assessment via 1–10 Likert visual analogue scale. (JPG)

S1 Table Linear mixed model data for the body composition model. Data are presented as the F-statistic and p-value, and a +/- symbol to denote a positive or negative linear association over time, where relevant. Where a significant linear relationship is observed, * denotes p < 0.05, ** denotes p < 0.01, *** denotes p < 0.001.
(DOCX)

S2 Table Linear mixed model data for the energy intake model. Data are presented as the F-statistic and p-value, and a +/- symbol to denote a positive or negative linear association over time, where relevant. Where a significant linear relationship is observed, * denotes p < 0.05, ** denotes p < 0.01, *** denotes p < 0.001.
(DOCX)

S3 Table Linear mixed model data for the appetite model. Data are presented as the F-statistic and p-value, and a +/- symbol to denote a positive or negative linear association over time, where relevant. Where a significant linear relationship is observed, * denotes p < 0.05, ** denotes p < 0.01, *** denotes p < 0.001.
(DOCX)

S4 Table Linear mixed model data for the biochemical markers model. Data are presented as the F-statistic and p-value, and a +/- symbol to denote a positive or negative linear association over time, where relevant. Where a significant linear relationship is observed, * denotes p < 0.05, ** denotes p < 0.01, *** denotes p < 0.001.
(DOCX)

S5 Table Linear mixed model data for the heart rate variability model. Data are presented as the F-statistic and p-value, and a +/- symbol to denote a positive or negative linear association over time, where relevant. Where a significant linear relationship is observed, * denotes p < 0.05, ** denotes p < 0.01, *** denotes p < 0.001.
(DOCX)

S6 Table Linear mixed model data for the cycling performance model. Data are presented as the F-statistic and p-value, and a +/- symbol to denote a positive or negative linear association over time, where relevant for the Modified Power Profile sprints and 4000m TT. Where a significant linear relationship is observed, * denotes p < 0.05, ** denotes p < 0.01, *** denotes p < 0.001. From the initial full model, variables considered non-significant following a backward model selection procedure and subsequently removed are denoted by #.
(DOCX)

S7 Table Linear mixed model data for the mood questionnaire tesponses model. Data are presented as the F-statistic and p-value, and a +/- symbol to denote a positive or negative linear association over time, where relevant for a) the MTDS and b) RESTQ-52 Sport. Where a significant linear relationship is observed,* denotes p < 0.05, ** denotes p < 0.01, *** denotes p < 0.001.
(DOCX)

S8 Table Raw data: Absolute RMR. Data are presented as individual values for each time point, and group mean ± SD.
(DOCX)

S9 Table Raw data: Relative RMR. Data are presented as individual values for each time point, and group mean ± SD.
(DOCX)

S10 Table Raw data: Minute ventilation [VE_(STPD)_]. Data are presented as individual values for each time point, and group mean ± SD.
(DOCX)

S11 Table Raw data: Body composition. Data are presented as individual values for each time point, and group mean ± SD.
(DOCX)

S12a-d Tables Raw data: Energy intake. Data are presented as individual values for each time point, and group mean ± SD.
(DOCX)

S13a-d Tables Raw data: Appetite. Data are presented as individual values for each time point, and group mean ± SD.
(DOCX)

S14a-b Tables Raw data: Biochemical markers PRE-POST ergometer warm-up. Data are presented as individual values for each time point, and group mean ± SD.
(DOCX)

S15a-b Tables Raw data: Heart rate variability. Data are presented as individual values for each time point, and group mean ± SD.
(DOCX)

S16a-e Tables Raw data: Cycling performance. Data are presented as individual values for each time point, and group mean ± SD.
(DOCX)

S17 Table Raw data: Mood questionnaires—Multicomponent training distress scale. Data are presented as individual values for each time point, and group mean ± SD.
(DOCX)

S18 Table Raw data: Mood questionnaires—RESTQ-52 sport. Data are presented as individual values for each time point, and group mean ± SD.
(DOCX)

## References

[1] ten Haaf T, van Staveren S, Oudenhoven E, Piacentini MF, Meeusen R, Roelands B, et al Subjective fatigue and readiness to train may predict functional overreaching after only 3 days of cycling. International Journal of Sports Physiology and Performance. 2017;12(Suppl 2):S2-87–S2-94. 10.1123/ijspp.2016-0404 27834554

[2] Aubry A, Hausswirth C, Louis J, Coutts A, Le Meur Y. Functional overreaching: the key to peak performance during the taper? Medicine and Science in Sport and Exercise. 2014;46(9):1769–77. doi: 10.1249/MSS.0000000000000301 25134000 10.1249/MSS.0000000000000301

[3] Kreider R, Fry A, O’Toole M. Overtraining in sport: terms, definitions, and prevalence In: Kreider R, Fry A, O’Toole M, editors. Overtraining in Sport. vii-ix Champaigne, IL: Human Kinetics; 1998.

[4] Meeusen R, Duclos M, Gleeson M, Rietjens G, Steinacker J, Urhausen A. Prevention, diagnosis and treatment of the overtraining syndrome. European Journal of Sport Science. 2006;6(1):1–14. 10.1249/MSS.0b013e318279a10a 23247672

[5] Meeusen R, Duclos M, Foster C, Fry A, Gleeson M, Nieman D, et al Prevention, diagnosis, and treatment of the overtraining syndrome: joint consensus statement of the European College of Sport Science and the American College of Sports Medicine. Medicine and Science in Sport and Exercise. 2013;45(1):186–205. doi: 10.1249/MSS.0b013e318279a10a 23247672 10.1249/MSS.0b013e318279a10a

[6] Urhausen A, Gabriel H, Kindermann W. Blood hormones as markers of training stress and overtraining. Sports Med. 1995;20(4):251–76. 8584849 10.2165/00007256-199520040-00004

[7] Halson S, Jeukendrup A. Does overtraining exist? Sports Med. 2004;34(14):967–81. 15571428 10.2165/00007256-200434140-00003

[8] Halson S. Monitoring training load to understand fatigue in athletes. Sports Med. 2014;44(Suppl 2):139–47. 10.1007/s40279-014-0253-z PMC4213373 25200666

[9] Petibois C, Cazorla G, Poortmans J, Déléris G. Biochemical aspects of overtraining in endurance sports. Sports Med. 2002;32(13):867–78. 12392446 10.2165/00007256-200232130-00005

[10] Wyatt F, Donaldson A, Brown E. The overtraining syndrome: A meta-analytic review. Journal of Exercise Physiology Online. 2013;16(2):12–23.

[11] Urhausen A, Kindermann W. Diagnosis of overtraining. Sports Med. 2002;32(2):95–102. 11817995 10.2165/00007256-200232020-00002

[12] Meeusen R, Piacentini MF, Busschaert B, Buyse L, De Schutter G, Stray-Gundersen J. Hormonal responses in athletes: the use of a two bout exercise protocol to detect subtle differences in (over)training status. Eur J Appl Physiol. 2004;91(2):140–6. 14523562 10.1007/s00421-003-0940-1

[13] Foster C, Lehmann M. Overtraining syndrome In: Guten G, editor. Running Injuries. Philadelphia: Saunders; 1997 p. 173–88.

[14] Barron J, Noakes T, Levy W, Smith C, Millar R. Hypothalamic dysfunction in overtrained athletes. The Journal of Clinical Endocrinology & Metabolism. 1985;60(4):803–6. 2982908 10.1210/jcem-60-4-803

[15] Hausswirth C, Louis J, Aubry A, Bonnet G, Duffield R, Le Meur Y. Evidence of disturbed sleep and increased illness in overreached endurance athletes. Medicine and Science in Sport and Exercise. 2014;46(5):1036–45. doi: 10.1249/MSS.0000000000000177 24091995 10.1249/MSS.0000000000000177

[16] Killer S, Svendsen I, Jeukendrup A, Gleeson M. Evidence of disturbed sleep and mood state in well-trained athletes during short-term intensified training with and without a high carbohydrate nutritional intervention. J Sports Sci. 2015;(Epub ahead of print). 10.1080/02640414.2015.1085589 26406911

[17] Halson S, Bridge M, Meeusen R, Busschaert B, Gleeson M, Jones D, et al Time course of performance changes and fatigue markers during intensified training in trained cyclists. J Appl Physiol. 2002;93(3):947–56. doi: 10.1152/japplphysiol.01164.2001 12183490 10.1152/japplphysiol.01164.2001

[18] Jeukendrup A, Hesselink M, Snyder A, Kuipers H, Keizer H. Physiological changes in male competitive cyclists after two weeks of intensified training. Int J Sports Med. 1992;13(7):534–41. doi: 10.1055/s-2007-1021312 1459749 10.1055/s-2007-1021312

[19] Kenttä G, Hassmén P, Raglin J. Mood state monitoring of training and recovery in elite kayakers. European Journal of Sport Science. 2006;6(4):245–53.

[20] Woods A, Garvican-Lewis L, Lundy B, Rice A, Thompson K. New approaches to determine fatigue in elite athletes during intensified training: Resting metabolic rate and pacing profile. PLoS ONE. 2017;12(3):e0173807 doi: 10.1371/journal.pone.0173807 28296943 10.1371/journal.pone.0173807 PMC5351856

[21] Speakman J, Selman C. Physical activity and resting metabolic rate. Proc Nutr Soc. 2003;62(03):621–34. 14692598 10.1079/PNS2003282

[22] Ihle R, Loucks A. Dose-response relationships between energy availability and bone turnover in young exercising women. J Bone Miner Res. 2004;19(8):1231–40. doi: 10.1359/JBMR.040410 15231009 10.1359/JBMR.040410

[23] Mountjoy M, Sundgot-Borgen J, Burke L, Carter S, Constantini N, Lebrun C, et al The IOC consensus statement: beyond the Female Athlete Triad—Relative Energy Deficiency in Sport (RED-S). Br J Sports Med. 2014;48(7):491–7. doi: 10.1136/bjsports-2014-093502 24620037 10.1136/bjsports-2014-093502

[24] Woods A, Garvican-Lewis L, Rice A, Thompson K. 12 days of altitude exposure at 1800 m does not increase resting metabolic rate in elite rowers. Appl Physiol Nutr Metab. 2017;Accepted 16th February. 10.1139/apnm-2016-0693 28278387

[25] Woods A, Sharma A, Garvican-Lewis L, Saunders P, Rice A, Thompson K. Four weeks of classical altitude training increases resting metabolic rate in highly trained middle-distance runners. International Journal of Sports Nutrition and Exercise Metabolism. 2017;27(1):83–90. 10.1123/ijsnem.2016-0116 27459673

[26] Keesey R, Powley T. Body energy homeostasis. Appetite. 2008;51(3):442–5. doi: 10.1016/j.appet.2008.06.009 18647629 10.1016/j.appet.2008.06.009 PMC2605663

[27] Blundell J, Finlayson G, Gibbons C, Caudwell P, Hopkins M. The biology of appetite control: Do resting metabolic rate and fat-free mass drive energy intake? Physiol Behav. 2015;152(Part B):473–8. Epub 31st May 2015. 26037633 10.1016/j.physbeh.2015.05.031

[28] De Pauw K, Roelands B, Cheung S, De Geus B, Rietjens G, Meeusen R. Guidelines to classify subject groups in sport-science research. International Journal of Sports Physiology and Performance. 2013;8(2):111–22. 23428482 10.1123/ijspp.8.2.111

[29] Roffey D, Byrne N, Hills A. Day-to-day variance in measurement of resting metabolic rate using ventilated-hood and mouthpiece & nose-clip indirect calorimetry systems. Journal of Parenteral and Enteral Nutrition. 2006;30(5):426–32. 16931612 10.1177/0148607106030005426

[30] Woods A, Garvican-Lewis L, Rice A, Thompson K. The ventilation-corrected ParvoMedics TrueOne 2400 provides a valid and reliable assessment of resting metabolic rate (RMR) in athletes compared with the Douglas Bag method. International Journal of Sports Nutrition and Exercise Metabolism. 2016;26(5):454–63. 10.1123/ijsnem.2015-0315 26841437

[31] TrainingPeaks. What is TSS? 2012 [cited 2016 29th August]. http://home.trainingpeaks.com/blog/article/what-is-tss.

[32] Tanner R, Gore C. Physiological tests for elite athletes: Human Kinetics; 2013.

[33] Garvican L, Pottgiesser T, Martin D, Schumacher Y, Barras M, Gore C. The contribution of haemoglobin mass to increases in cycling performance induced by simulated LHTL. Eur J Appl Physiol. 2011;111(6):1089–101. doi: 10.1007/s00421-010-1732-z 21113616 10.1007/s00421-010-1732-z

[34] Saunders P, Telford R, Pyne D, Cunningham R, Gore C, Hahn A, et al Improved running economy in elite runners after 20 days of simulated moderate-altitude exposure. J Appl Physiol. 2004;96(3):931–7. doi: 10.1152/japplphysiol.00725.2003 14607850 10.1152/japplphysiol.00725.2003

[35] Kuipers H, Verstappen F, Keizer H, Geurten P, Van Kranenburg G. Variability of aerobic performance in the laboratory and its physiologic correlates. Int J Sports Med. 1985;6(04):197–201. 4044103 10.1055/s-2008-1025839

[36] Lithander F, Strik C, McGill A, MacGibbon A, McArdle B, Poppitt S. No effect of an oleoylethanolamide-related phospholipid on satiety and energy intake: a randomised controlled trial of phosphatidylethanolamine. Lipids Health Dis. 2008;7(41). 10.1186/1476-511X-7-41 PMC2600636 18957134

[37] Tarvainen M, Niskanen J. Kubio user guide. Biosignal Analysis and Medical Imaging Group, 2008.

[38] Borg G. Psychophysical scaling with applications in physical work and the perception of exertion. Scand J Work Environ Health. 1990;16(1):55–8. 2345867 10.5271/sjweh.1815

[39] Martin D, Ebert T, Quod M, Lee H, Stephens B, Schumacher Y. Alpe D’Huez: Direct quantification of the uphill cycling power output-speed relationship. 12th Annual Congress of the ECSS; Jyväskylä, Finland2007.

[40] Main L, Grove J. A multi-component assessment model for monitoring training distress among athletes. European Journal of Sport Science. 2009;9(4):195–202.

[41] Kellmann M, Kallus K. Recovery-stress questionnaire for athletes: User manual: Human Kinetics; 2001.

[42] Melin A, Tornberg A, Skouby S, Moller S, Sundgot-Borgen J, Faber J, et al Energy availability and the female athlete triad in elite endurance athletes. Scand J Med Sci Sports. 2014;25(1):610–22. Epub 04/06/2014. 24888644 10.1111/sms.12261

[43] De Souza M, West S, Jamal S, Hawker G, Gundberg C, Williams N. The presence of both an energy deficiency and estrogen deficiency exacerbate alterations of bone metabolism in exercising women. Bone. 2008;43:140–8. doi: 10.1016/j.bone.2008.03.013 18486582 10.1016/j.bone.2008.03.013

[44] Koehler K, Williams N, Mallinson R, Southmayd E, Allaway H, De Souza M. Low resting metabolic rate in exercise-associated amenorrhea is not due to a reduced proportion of highly metabolically active tissue compartments. American Journal of Physiology—Endocrinology and Metabolism. 2016;Epub ahead of print. 10.1152/ajpendo.00110.2016 27382033

[45] Tenforde A, Barrack M, Nattiv A, Fredericson M. Parallels with the female athlete triad in male athletes. Sports Med. 2016;46(2):171–82. doi: 10.1007/s40279-015-0411-y 26497148 10.1007/s40279-015-0411-y

[46] Piacentini MF, Witard O, Tonoli C, Jackman S, Turner J, Kies A, et al Effect of intensive training on mood with no effect on brain-derived neurotrophic factor. International Journal of Sports Physiology & Performance. 2016;11(6):824–30. 26658294 10.1123/ijspp.2015-0279

[47] Halson S, Lancaster G, Jeukendrup A, Gleeson M. Immunological responses to overreaching in cyclists. Medicine and Science in Sport and Exercise. 2003;35(5):854–61. doi: 10.1249/01.MSS.0000064964.80040.E9 12750597 10.1249/01.MSS.0000064964.80040.E9

[48] Decroix L, Piacentini MF, Rietjens G, Meeusen R. Monitoring physical and cognitive overload during a training camp in professional female cyclists. International Journal of Sports Physiology & Performance. 2016;11(7):933–9. 26816388 10.1123/ijspp.2015-0570

[49] Slivka D, Hailes W, Cuddy J, Ruby B. Effects of 21 days of intensified training on markers of overtraining. The Journal of Strength & Conditioning Research. 2010;24(10):2604–12. 20733522 10.1519/JSC.0b013e3181e8a4eb

[50] Berger BG, Motl RW, Butki BD, Martin DT, Wilkinson JG, Owen DR. Mood and cycling performance in response to three weeks of high-intensity, short-duration overtraining, and a two-week taper. The Sport Psychologist. 1999;13:444–57.

[51] Clark B, Costa V, O’Brien B, Guglielmo L, Paton C. Effects of a seven day overload-period of high-intensity training on performance and physiology of competitive cyclists. PLoS ONE. 2014;9(12):e115308 doi: 10.1371/journal.pone.0115308 25521824 10.1371/journal.pone.0115308 PMC4270748

[52] Rietjens G, Kuipers H, Adam J, Saris W, Breda E, Hamont D, et al Physiological, biochemical and psychological markers of strenuous training-induced fatigue. Int J Sports Med. 2005;26(1):16–26. doi: 10.1055/s-2004-817914 15643530 10.1055/s-2004-817914

[53] Jürimäe J, Mäestu J, Purge P, Jürimäe T. Changes in stress and recovery after heavy training in rowers. J Sci Med Sport. 2004;7(3):335–9. 15518298 10.1016/s1440-2440(04)80028-8

[54] Main L, Warmington S, Korn E, Gastin P. Utility of the multi-component training distress scale to monitor swimmers during periods of training overload. Res Sports Med. 2016;24(3):254–65. 10.1080/15438627.2016.1202828 27368060

[55] Otter R, Brink M, van der Does H, Lemmink K. Monitoring perceived stress and recovery in relation to cycling performance in female athletes. Int J Sports Med. 2016;37(1):12–8. doi: 10.1055/s-0035-1555779 26509384 10.1055/s-0035-1555779

[56] de Koning J, Bobbert M, Foster C. Determination of optimal pacing strategy in track cycling with an energy flow model. J Sci Med Sport. 1999;2(3):266–77. 10668763 10.1016/s1440-2440(99)80178-9

[57] Shei R, Thompson K, Chapman R, Raglin J, Mickleborough T. Using deception to establish a reproducible improvement in 4-km cycling time trial performance. Int J Sports Med. 2016;37(5):341–6. doi: 10.1055/s-0035-1565139 26855435 10.1055/s-0035-1565139

[58] Saw A, Main L, Gastin P. Monitoring the athlete training response: subjective self-reported measures trump commonly used objective measures: a systematic review. Br J Sports Med. 2015;Epub ahead of print. 10.1136/bjsports-2015-094758 PMC4789708 26423706

[59] Comotto S, Bottoni A, Moci E, Piacentini MF. Analysis of session-RPE and profile of mood states during a triathlon training camp. The Journal of Sports Medicine and Physical Fitness. 2015;55(4):361–7. 25289712

[60] Poehlman E, Melby C, Badylak S. Resting metabolic rate and postprandial thermogenesis in highly trained and untrained males. The American Journal of Clinical Nutrition. 1988;47(5):793–8. 3284328 10.1093/ajcn/47.5.793

[61] Laforgia J, Van Der Ploeg G, Withers R, Gunn S, Brooks A, Chatterton B. Impact of indexing resting metabolic rate against fat-free mass determined by different body composition models. Eur J Clin Nutr. 2004;58(8):1132–41. doi: 10.1038/sj.ejcn.1601941 15054426 10.1038/sj.ejcn.1601941

[62] Donahoo W, Levine J, Melanson E. Variability in energy expenditure and its components. Curr Opin Clin Nutr Metab Care. 2004;7(6):599–605. 15534426 10.1097/00075197-200411000-00003

[63] Westerterp K, Meijer G, Schoffelen P, Janssen E. Body mass, body composition and sleeping metabolic rate before, during and after endurance training. Eur J Appl Physiol. 1994;69(3):203–8. 10.1007/BF01094789 8001530

[64] Dolezal B, Potteiger J, Jacobsen D, Benedict S. Muscle damage and resting metabolic rate after acute resistance exercise with an eccentric overload. Medicine and Science in Sport and Exercise. 2000;32(7):1202–7. 10912882 10.1097/00005768-200007000-00003

[65] Scharhag-Rosenberger F, Morsch A, Wegmann M, Ruppenthal S, Kaestner L, Meyer T, et al Irisin does not mediate resistance training-induced alterations in RMR. Medicine and Science in Sport and Exercise. 2014;46(9):1736–43. doi: 10.1249/MSS.0000000000000286 24566753 10.1249/MSS.0000000000000286

[66] Burt D, Lamb K, Nicholas C, Twist C. Effects of exercise-induced muscle damage on resting metabolic rate, sub-maximal running and post-exercise oxygen consumption. European Journal of Sport Science. 2014;14(4):337–44. doi: 10.1080/17461391.2013.783628 23566074 10.1080/17461391.2013.783628

[67] Meuret J. A comparison of effects between post exercise resting metabolic rate after thirty minutes of intermittent treadmill and resistance exercise. Electronic Theses, Treatises and Dissertations [Internet]. 2007:[Paper 2461 p.]. http://diginole.lib.fsu.edu/etd/2461.

[68] Sirithienthad P. Comparison of the effects of post exercise basal metabolic rate among continuous aerobic, intermittent aerobic, and resistance exercise. Tallahassee, Florida Florida State University; 2006.

[69] Knab A, Shanely R, Corbin K, Jin F, Sha W, Nieman D. A 45-minute vigorous exercise bout increases metabolic rate for 14 hours. Medicine and Science in Sport and Exercise. 2011;43(9):1643–8. doi: 10.1249/MSS.0b013e3182118891 21311363 10.1249/MSS.0b013e3182118891

[70] Larsen I, Welde B, Martins C, Tjønna A. High- and moderate-intensity aerobic exercise and excess post-exercise oxygen consumption in men with metabolic syndrome. Scand J Med Sci Sports. 2014;24(3):e174–e9. doi: 10.1111/sms.12132 24118097 10.1111/sms.12132

[71] Schaal K, Tiollier E, Le Meur Y, Casazza G, Hausswirth C. Elite synchronized swimmers display decreased energy availability during intensified training. Scand J Med Sci Sports. 2016;Epub ahead of print. Epub 1st July. 10.1111/sms.12716 27367601

[72] Hopkins M, Finlayson G, Duarte C, Whybrow S, Ritz P, Horgan G, et al Modelling the associations between fat-free mass, resting metabolic rate and energy intake in the context of total energy balance. Int J Obes. 2016;40(2):312–8. 10.1038/ijo.2015.155 26278004

[73] Halson S, Lancaster G, Achten J, Gleeson M, Jeukendrup A. Effects of carbohydrate supplementation on performance and carbohydrate oxidation after intensified cycling training. J Appl Physiol. 2004;97(4):1245–53. doi: 10.1152/japplphysiol.01368.2003 15155717 10.1152/japplphysiol.01368.2003

[74] Achten J, Halson S, Moseley L, Rayson M, Casey A, Jeukendrup A. Higher dietary carbohydrate content during intensified running training results in better maintenance of performance and mood state. J Appl Physiol. 2004;96(4):1331–40. doi: 10.1152/japplphysiol.00973.2003 14660506 10.1152/japplphysiol.00973.2003

[75] Costa R, Jones G, Lamb K, Coleman R, Williams J. The effects of a high carbohydrate diet on cortisol and salivary immunoglobulin A (s-IgA) during a period of increase exercise workload amongst Olympic and Ironman triathletes. Int J Sports Med. 2005;26(10):880–5. doi: 10.1055/s-2005-837467 16320174 10.1055/s-2005-837467

[76] Svendsen I, Killer S, Carter J, Randell R, Jeukendrup A, Gleeson M. Impact of intensified training and carbohydrate supplementation on immunity and markers of overreaching in highly trained cyclists. Eur J Appl Physiol. 2016;116(5):867–77. doi: 10.1007/s00421-016-3340-z 26908041 10.1007/s00421-016-3340-z PMC4834106

[77] Popovic V, Duntas L. Leptin TRH and ghrelin: influence on energy homeostasis at rest and during exercise. Horm Metab Res. 2005;37(9):533–7. doi: 10.1055/s-2005-870418 16175489 10.1055/s-2005-870418

[78] Mäestu J, Jürimäe J, Jürimäe T. Monitoring of performance and training in rowing. Sports Med. 2005;35(7):597–617. 16026173 10.2165/00007256-200535070-00005

[79] McMurray R, Hackney A. Interactions of metabolic hormones, adipose tissue and exercise. Sports Med. 2005;35(5):393–412. 15896089 10.2165/00007256-200535050-00003

[80] Desgorces F, Chennaoui M, Gomez-Merino D, Drogou C, Guezennec C. Leptin response to acute prolonged exercise after training in rowers. Eur J Appl Physiol. 2004;91(5–6):677–81. doi: 10.1007/s00421-003-1030-0 14704800 10.1007/s00421-003-1030-0

[81] Park H, Ahima R. Physiology of leptin: energy homeostasis, neuroendocrine function and metabolism. Metabolism. 2015;64(1):24–34. doi: 10.1016/j.metabol.2014.08.004 25199978 10.1016/j.metabol.2014.08.004 PMC4267898

[82] Karl J, Smith T, Wilson M, Bukhari A, Pasiakos S, McClung H, et al Altered metabolic homeostasis is associated with appetite regulation during and following 48-h of severe energy deprivation in adults. Metabolism—Clinical and Experimental. 2016;65(4):416–27. doi: 10.1016/j.metabol.2015.11.001 26975533 10.1016/j.metabol.2015.11.001

[83] Koehler K, Hoerner N, Gibbs J, Zinner C, Braun H, De Souza M, et al Low energy availability in exercising men is associated with reduced leptin and insulin but not with changes in other metabolic hormones. J Sports Sci. 2016;34(20):1921–9. doi: 10.1080/02640414.2016.1142109 26852783 10.1080/02640414.2016.1142109

[84] Kyriakidis M, Caetano L, Anastasiadou N, Karasu T, Lashen H. Functional hypothalamic amenorrhoea: leptin treatment, dietary intervention and counselling as alternatives to traditional practice–systematic review. European Journal of Obstetrics & Gynecology and Reproductive Biology. 2016;198:131–7. 26849039 10.1016/j.ejogrb.2016.01.018

[85] Gibbs J, Mallinson R, De Souza M. Hormonal and reproductive changes associated with physical activity and exercise In: Vaamonde D, du Plessis SS, Agarwal A, editors. Exercise and Human Reproduction: Induced Fertility Disorders and Possible Therapies. New York, NY: Springer 2016 p. 187–207.

[86] Jürimäe J, Mäestu J, Jürimäe T. Leptin as a marker of training stress in highly trained male rowers? Eur J Appl Physiol. 2003;90(5–6):533–8. doi: 10.1007/s00421-003-0879-2 12905045 10.1007/s00421-003-0879-2

[87] Rämson R, Jürimäe J, Jürimäe T, Mäestu J. The influence of increased training volume on cytokines and ghrelin concentration in college level male rowers. Eur J Appl Physiol. 2008;104(5):839–46. doi: 10.1007/s00421-008-0839-y 18665387 10.1007/s00421-008-0839-y

[88] Goto K, Shioda K, Uchida S. Effect of 2 days of intensive resistance training on appetite-related hormone and anabolic hormone responses. Clin Physiol Funct Imaging. 2013;33(2):131–6. doi: 10.1111/cpf.12005 23383691 10.1111/cpf.12005

[89] Trexler E, Smith-Ryan A, Norton L. Metabolic adaptation to weight loss: implications for the athlete. J Int Soc Sports Nutr. 2014;11(1):7 doi: 10.1186/1550-2783-11-7 24571926 10.1186/1550-2783-11-7 PMC3943438

[90] Bianco A, Maia A, da Silva W, Christoffolete M. Adaptive activation of thyroid hormone and energy expenditure. Biosci Rep. 2005;25(3–4):191–208. doi: 10.1007/s10540-005-2885-6 16283553 10.1007/s10540-005-2885-6

[91] Johansen K, Hansen J, Skovsted L. The preferential role of triiodothyronine in the regulation of basal metabolic rate in hyper- and hypothyroidism. Acta Med Scand. 1978;204(1–6):357–9. 717056 10.1111/j.0954-6820.1978.tb08454.x

[92] Kim B. Thyroid hormone as a determinant of energy expenditure and the basal metabolic rate. Thyroid. 2008;18(2):141–4. doi: 10.1089/thy.2007.0266 18279014 10.1089/thy.2007.0266

[93] Henning P, Scofield D, Spiering B, Staab J, Matheny R, Smith M, et al Recovery of endocrine and inflammatory mediators following an extended energy deficit. The Journal of Clinical Endocrinology & Metabolism. 2014;99(3):956–64. 24423293 10.1210/jc.2013-3046

[94] Ortiga-Carvalho T, Chiamolera M, Pazos-Moura C, Wondisford FE. Hypothalamus-Pituitary-Thyroid Axis. Comprehensive Physiology. 2016;6(3):1387–428. doi: 10.1002/cphy.c150027 27347897 10.1002/cphy.c150027

[95] Da Silva V, De Oliveira N, Silveira H, Mello R, Deslandes A. Heart rate variability indexes as a marker of chronic adaptation in athletes: A systematic review. Ann Noninvasive Electrocardiol. 2015;20(2):108–18. doi: 10.1111/anec.12237 25424360 10.1111/anec.12237 PMC6931675

[96] Fronczek R, Overeem S, Reijntjes R, Lammers G, van Dijk J, Pijl H. Increased heart rate variability but normal resting metabolic rate in hypocretin/orexin-deficient human narcolepsy. J Clin Sleep Med. 2008;4(3):248–54. 18595438 PMC2546458

[97] Plews D, Laursen P, Buchheit M. Day-to-day heart rate variability (HRV) recordings in World Champion rowers: Appreciating unique athlete characteristics. International Journal of Sports Physiology & Performance. 2016;(Epub ahead of print). 10.1123/ijspp.2016-0343 27736257

[98] Bellenger C, Karavirta L, Thomson R, Robertson E, Davison K, Buckley J. Contextualising parasympathetic hyperactivity in functionally overreached athletes with perceptions of training tolerance. International Journal of Sports Physiology & Performance. 2016;11(7):685–92. 26640275 10.1123/ijspp.2015-0495

[99] Le Meur Y, Pichon A, Schaal K, Schmitt L, Louis J, Gueneron J, et al Evidence of parasympathetic hyperactivity in functionally overreached athletes. Medicine and Science in Sport and Exercise. 2013;45(11):2061–71. doi: 10.1249/MSS.0b013e3182980125 24136138 10.1249/MSS.0b013e3182980125

[100] Pichot V, Busso T, Roche F, Garet M, Costes F, Duverney D, et al Autonomic adaptations to intensive and overload training periods: a laboratory study. Medicine and Science in Sport and Exercise. 2002;34(10):1660–6. doi: 10.1249/01.MSS.0000035993.08625.31 12370569 10.1097/00005768-200210000-00019

