# Supplementary material for: The effects of intensified training on resting metabolic rate (RMR), body composition and performance in trained cyclists
Source: PLoS One. 2018 Feb 14;13(2):e0191644. doi: 10.1371/journal.pone.0191644 (PMC5812577; doi:10.1371/journal.pone.0191644)
Supplement: S1 Table — Data are presented as the F-statistic and p-value, and a +/- symbol to denote a positive or negative linear association over time, where relevant. Where a significant linear relationship is observed, * denotes p < 0.05, ** denotes p < 0.01, *** denotes p < 0.001. (DOCX) [file pone.0191644.s002.docx]

**S1 Table:**

|  | **Training Block** | **Training Stress Score (TSS)** | **Total energy intake (mJ.day^-1^)** | **Appetite: Hunger** | **Appetite: Fullness** | **Appetite: Satiety** | **Appetite: Eat now** | **Training Block*TSS** |
| --- | --- | --- | --- | --- | --- | --- | --- | --- |
| **Body mass (kg)** | F_(2, 19.242)_ = 4.3362,  p = 0.03* | F_(1, 9.5037)_ = 0.3332,  P = 0.5772 | F_(1, 19.2)_ = 7.2183,  p = 0.01* (+) | F_(1, 10.7414)_ = 1.4162,  p = 0.26 | F_(1, 11.5047)_ = 0.9596,  p = 0.35 | F_(1, 9.9297)_ = 2.4007,  p = 0.15 | F_(1, 9.362)_ = 1.9781,  p = 0.19 | - |
| **Fat mass (kg)** | F_(2, 20.35)_ = 56.2494,  p=<0.001*** | F_(1, 20.583)_ = 9.6979,  p = 0.005** | F_(1, 14.245)_ = 1.4055,  p = 0.26 | F_(1, 15.254)_ = 0.5271,  p = 0.48 | F_(1, 17.828_ = 0.3384,  p = 0.57 | F_(1, 14.072)_ = 1.132,  p = 0.31 | F_(1, 13.17)_ = 0.0135,  p = 0.91 | F_(2, 20.176)_ = 5.5038,  p = 0.01* |
| **Fat-free mass (kg)** | F_(2, 14.661)_ = 0.2719,  p = 0.77 | F_(1, 15.082)_ = 1.7195,  p = 0.21 | F_(1, 15.32)_ = 0.0469,  p = 0.83 | F_(1, 16.344)_ = 0.221,  p = 0.64 | F_(1, 19.024_ = 0.3155,  p = 0.58 | F_(1, 15.383)_ = 0.512,  p = 0.49 | F_(1, 14.636)_ = 0.6418,  p = 0.44 | - |

*TSS = Training stress score; CHO = carbohydrate; Appetite: Eat Now = response to “How much do you think you could eat now?”*
