# Supplementary material for: The effects of intensified training on resting metabolic rate (RMR), body composition and performance in trained cyclists
Source: PLoS One. 2018 Feb 14;13(2):e0191644. doi: 10.1371/journal.pone.0191644 (PMC5812577; doi:10.1371/journal.pone.0191644)
Supplement: S2 Table — Data are presented as the F-statistic and p-value, and a +/- symbol to denote a positive or negative linear association over time, where relevant. Where a significant linear relationship is observed, * denotes p < 0.05, ** denotes p < 0.01, *** denotes p < 0.001. (DOCX) [file pone.0191644.s003.docx]

**S2 Table:**

|  | **Training Block** | **Body Mass (kg)** | **Fat mass (kg)** | **Fat-free mass (kg)** | **Appetite: Hunger** | **Appetite: Fullness** | **Appetite: Satiety** | **Appetite: Eat now** |
| --- | --- | --- | --- | --- | --- | --- | --- | --- |
| **Total energy intake**  **(mJ.day^-1^)** | F_(2, 23.346)_ = 3.2149,  p = 0.06 | F_(1, 10.966)_ = 9.6844,  p = 0.0099** (+) | F_(1, 17.347)_ = 11.3443,  p = 0.004** (-) | F_(1, 23.394)_ = 3.8304,  p = 0.06 | F_(1, 21.337)_ =  11.03,  p = 0.003** (+) | F_(1, 25.923)_ = 0.1024,  p = 0.75 | F_(1, 32.939)_ = 7.3092,  p = 0.01* (+) | F_(1, 24.996)_ = 0.2787,  p = 0.60 |
| **CHO (g)** | F_(5, 125)_ =  3.4769,  p = 0.006* | F_(1, 27.78)_ = 0.3572,  p = 0.55 | F_(1, 24.952)_ =  2.0718,  p = 0.16 | F_(1, 26.508)_ = 0.0445,  p = 0.83 | F_(1, 27.836)_ =  3.553  p = 0.07 | F_(1, 23.015)_ = 1.9401,  p = 0.18 | F_(1, 20.809)_ = 1.4824,  p = 0.24 | F_(1, 19.544)_ = 0.2181,  p = 0.65 |
| **Fat (g)** | F_(2, 23.006)_ = 0.6866,  p = 0.51 | F_(1, 32.71)_ =  6.1619  p = 0.02* (+) | F_(1, 32.999)_ =  7.7408,  p = 0.009** (-) | F_(1, 32.825)_ = 4.1651,  p = 0.049* (-) | F_(1, 27.839)_ = 1.5483,  p = 0.22 | F_(1, 23.612)_ =  1.106,  p = 0.30 | F_(1, 28.851)_ = 4.8087,  p = 0.04* (+) | F_(1, 20.499)_ = 0.1334,  p = 0.72 |
| **Protein (g)** | F_(2, 24.079)_ = 1.1365  p = 0.34 | F_(1, 10.48)_ = 18.023,  p = 0.002** (+) | F_(1, 12.409)_ =  24.856,  p = 0.0003*** (-) | F_(1, 13.627)_ = 1.3181,  p = 0.27 | F_(1, 13.477)_ = 3.5212,  p = 0.08 | F_(1, 27.857)_ = 0.1379,  p = 0.71 | F_(1, 24.554)_ = 0.8829,  p = 0.36 | F_(1, 27.267)_ = 0.0358,  p = 0.85 |

*CHO = carbohydrate; Appetite: Eat Now = response to “How much do you think you could eat now?”*
