# Supplementary material for: The effects of intensified training on resting metabolic rate (RMR), body composition and performance in trained cyclists
Source: PLoS One. 2018 Feb 14;13(2):e0191644. doi: 10.1371/journal.pone.0191644 (PMC5812577; doi:10.1371/journal.pone.0191644)
Supplement: S3 Table — Data are presented as the F-statistic and p-value, and a +/- symbol to denote a positive or negative linear association over time, where relevant. Where a significant linear relationship is observed, * denotes p < 0.05, ** denotes p < 0.01, *** denotes p < 0.001. (DOCX) [file pone.0191644.s004.docx]

**S3 Table:**

|  | **Training Block** | **Absolute RMR**  **(kJ.day^-1^)** | **Leptin**  **(% change)** | **RESTQ-52:**  **Total Stress** | **RESTQ-52:**  **Total Recovery** |
| --- | --- | --- | --- | --- | --- |
| **How hungry do you feel?** | F_(2, 20.905)_ =  2.892,  p = 0.08 | F_(1, 21.886)_ =  0.581,  p = 0.45 | F_(1, 27.553)_ =  6.6828,  p = 0.02* (+) | - | F_(1, 22.228)_ =  0,  p = 1.00 |
| **How full do you feel?** | F_(2, 24.247)_ =  0.7659,  p = 0.48 | - | - | F_(1, 19.275)_ = 0.6977,  p = 0.41 | - |
| **How satisfied do you feel?** | F_(2, 25.187)_ =  1.7228,  p = 0.20 | - | - | - | F_(1, 33.189)_ =  5.2792,  p = 0.03* (-) |
| **How much do you think you could eat now?** | F_(5, 122.49)_ =  3.9196,  p = 0.002** | F_(1, 108.39)_ =  17.5478,  p = <0.001*** (-) | - | - | - |
