# Supplementary material for: The effects of intensified training on resting metabolic rate (RMR), body composition and performance in trained cyclists
Source: PLoS One. 2018 Feb 14;13(2):e0191644. doi: 10.1371/journal.pone.0191644 (PMC5812577; doi:10.1371/journal.pone.0191644)
Supplement: S4 Table — Data are presented as the F-statistic and p-value, and a +/- symbol to denote a positive or negative linear association over time, where relevant. Where a significant linear relationship is observed, * denotes p < 0.05, ** denotes p < 0.01, *** denotes p < 0.001. (DOCX) [file pone.0191644.s005.docx]

**S4 Table:**

|  | **Training Block** | **Training Stress Score (TSS)** | **Absolute RMR (kJ.day^-1^)** |
| --- | --- | --- | --- |
| **Leptin**  **(% change)** | F_(5, 83.769)_ =  0.4916,  p = 0.78 | F_(1, 27.734)_ =  2.5999,  p = 0.12 | F_(1, 19.486)_ =  0.1382,  p = 0.71 |
| **fT3**  **(% change)** | F_(5, 83.526)_ =  0.0945,  p = 0.99 | F_(1, 38.307)_ =  0.0681,  p = 0.80 | F_(1, 22.597)_ =  0.2622,  p = 0.61 |

*fT3 = free thyroid hormone (triiodothyronine); TSS = Training stress score*
