# Supplementary material for: The effects of intensified training on resting metabolic rate (RMR), body composition and performance in trained cyclists
Source: PLoS One. 2018 Feb 14;13(2):e0191644. doi: 10.1371/journal.pone.0191644 (PMC5812577; doi:10.1371/journal.pone.0191644)
Supplement: S5 Table — Data are presented as the F-statistic and p-value, and a +/- symbol to denote a positive or negative linear association over time, where relevant. Where a significant linear relationship is observed, * denotes p < 0.05, ** denotes p < 0.01, *** denotes p < 0.001. (DOCX) [file pone.0191644.s006.docx]

**S5 Table:**

|  | **Training Block** | **Training Stress Score (TSS)** | **Absolute RMR**  **(kJ.day^-1^)** | **Relative RMR (cal.kg.FFM^-1^)** | **Leptin**  **(% change)** | **fT3**  **(% change)** | **Training Block* Relative RMR** |
| --- | --- | --- | --- | --- | --- | --- | --- |
| **HRV (LnRMSSD)** | F_(2, 21.081)_ =  0.2841,  p = 0.76 | F_(1, 15.507)_ =  0.2997,  p = 0.59 | F_(1, 7.0536)_ =  0.6627,  p = 0.44 | F_(1, 14.709)_ =  1.678,  p = 0.69 | F_(1, 18.8326)_ =  0.5991,  p = 0.45 | F_(1, 22.122)_ = 4.5974,  p = 0.04*(+) | F_(2, 22.608)_ = 6.5212,  p = 0.005** |

*HRV = heart rate variability; TSS = Training stress score; fT3 = free thyroid hormone (triiodothyronine)*
