# Supplementary material for: The effects of intensified training on resting metabolic rate (RMR), body composition and performance in trained cyclists
Source: PLoS One. 2018 Feb 14;13(2):e0191644. doi: 10.1371/journal.pone.0191644 (PMC5812577; doi:10.1371/journal.pone.0191644)
Supplement: S6 Table — Data are presented as the F-statistic and p-value, and a +/- symbol to denote a positive or negative linear association over time, where relevant for the Modified Power Profile sprints and 4000m TT. Where a significant linear relationship is observed, * denotes p < 0.05, ** denotes p < 0.01, *** denotes p < 0.001. From the initial full model, variables considered non-significant following a backward model selection procedure and subsequently removed are denoted by #. (DOCX) [file pone.0191644.s007.docx]

**S6 Table:**

|  | **Training Block** | **Training Stress Score (TSS)** | **Absolute RMR**  **(kJ.day^-1^)** | **HRV**  **(LnRMSSD)** | **RESTQ-52: Total Stress** | **Training Block*TSS** | **Training Block* RESTQ-52 Total Stress** |
| --- | --- | --- | --- | --- | --- | --- | --- |
| **15 s power output (W)** | F_(5, 121.87)_ =  8.2622,  p=<0.001*** | F_(1, 128.63)_ = 9.1628,  p = 0.003** (-) | F_(1, 76.658)_ = 0.7536,  p = 0.39 | F_(1, 83.032_ = 0.0745,  p = 0.79 | F_(1, 131.9)_ = 3.6235,  p = 0.06 | **-** | F_(5, 120.94)_ = 2.6092,  p = 0.03* |
| **4000 m TT power output (W)** | F_(5, 118.26)_ =  2.766,  p = 0.02* | F_(1, 127.4)_ =  6.2739,  p = 0.01* | F_(1, 88.395)_ = 3.5634,  p = 0.06 | F_(1, 93.458)_ = 2.0428,  p = 0.16 | F_(1, 123.12)_ = 1.268,  p = 0.26# | F_(5, 117.77)_ = 3.6222,  p = 0.004** | F_(5, 118.51)_ = 2.4486,  p = 0.04* |
| **4000 m TT HR (bpm)** | F_(5, 137.02)_ =  2.6307,  p = 0.03* | F_(1, 98.688)_ = 0.3027,  p = 0.58 | F_(1, 98.36)_ = 01.6674,  p = 0.20 | F_(1, 91.276)_ = 0.1946,  p = 0.66 | F_(1, 145.89)_ = 7.5712,  p = 0.007** (-) | **-** | **-** |
| **4000 m TT BLa**  **(mmol.L^-1^)** | F_(4, 97.456)_ =  1.5632,  p = 0.19 | F_(1, 103.979)_ = 0.0337,  p = 0.85 | F_(1, 98.521)_ = 6.0387,  p = 0.02* (+) | F_(1, 95.228)_ = 0.4891,  p = 0.49 | **-** | **-** | **-** |
| **4000 m TT RPE** | F_(5, 137.01)_ =  3.1083,  p = 0.01* | F_(1, 103.938)_ = 1.2784,  p = 0.26 | F_(1, 72.403)_ = 0.101,  p = 0.75 | F_(1, 94.868_ = 0.8565,  p = 0.36 | **-** | **-** | **-** |

*BLa = blood lactate concentration; RPE = Rating of Perceived Exertion (6-20 Borg Scale); HR = maximum heart rate*
