# Supplementary material for: The effects of intensified training on resting metabolic rate (RMR), body composition and performance in trained cyclists
Source: PLoS One. 2018 Feb 14;13(2):e0191644. doi: 10.1371/journal.pone.0191644 (PMC5812577; doi:10.1371/journal.pone.0191644)
Supplement: S7 Table — Data are presented as the F-statistic and p-value, and a +/- symbol to denote a positive or negative linear association over time, where relevant for a) the MTDS and b) RESTQ-52 Sport. Where a significant linear relationship is observed,* denotes p < 0.05, ** denotes p < 0.01, *** denotes p < 0.001. (DOCX) [file pone.0191644.s008.docx]

**S7 Table:**

|  | **Training Block** | **Training Stress Score (TSS)** | | **HRV (LnRMSSD)** | | **Training Block*HRV** | | **Training Block*TSS** | |  |
| --- | --- | --- | --- | --- | --- | --- | --- | --- | --- | --- |
| **MTDS:**  **Total Mood Disturbance** | F_(5, 110.61)_ =  8.1159,  p = <0.001*** | F_(1, 120.87)_ = 4.2109,  p = 0.04* (-) | | F_(1, 111.95)_ = 5.0604,  p = 0.03* (+) | | - | | - | |  |
| **RESTQ-52 Sport: Total Stress** | F_(5, 164)_ =  20.194,  p = <0.001*** | | F_(1, 114.512)_ =  1.0924,  p = 0.30 | | F_(1, 107.529)_ =  0.5328,  p = 0.47 | | - | | - | |
| **RESTQ-52 Sport: Total Recovery** | F_(5, 105.09)_ =  3.2111,  p = <0.001*** | | F_(1, 110.86)_ =  0.6484,  p = 0.42 | | F_(1, 105.78)_ =  1.9524,  p = 0.17 | | F_(5, 105.92)_ = 2.3886,  p = 0.04* | | - | |

*TSS = Training stress score; HRV = heart rate variability*
