# Supplementary material for: The effects of intensified training on resting metabolic rate (RMR), body composition and performance in trained cyclists
Source: PLoS One. 2018 Feb 14;13(2):e0191644. doi: 10.1371/journal.pone.0191644 (PMC5812577; doi:10.1371/journal.pone.0191644)
Supplement: S8 Table — Data are presented as individual values for each time point, and group mean ± SD. (DOCX) [file pone.0191644.s009.docx]

|  | **Absolute RMR (kJ.day^-1)^** | | | | | | | | | | |
| --- | --- | --- | --- | --- | --- | --- | --- | --- | --- | --- | --- |
| **Training Block** | **Baseline** | **Build** | | **Loading 1** | | | **Loading 2** | | | **Recovery 1** | **Recovery 2** |
| **Participant** | **Day 1** | **Day 9** | **Day 12** | **Day 15** | **Day 17** | **Day 19** | **Day 22** | **Day 26** | **Day 29** | **Day 33** | **Day 40** |
| 1 | 7571 | 7498 | 7538 | 7763 | 8057 | 8095 | 7743 | 6717 | 6267 | 7578 | 7693 |
| 2 | 7749 | 7621 | 7999 | 7820 | 8203 | 7903 | 8252 | 6764 | 6530 | 8368 | 7774 |
| 3 | 9527 | 9548 | 10252 | 10532 | 10302 | 10294 | 9575 | 9053 | 11076 | 10115 | 9533 |
| 4 | 7742 | 7397 | 7359 | 6821 | 7342 | 7203 | 7054 | 6217 | 5921 | 6940 | 7074 |
| 5 | 9360 | 9169 | 8812 | 9042 | 9024 | 9112 | 9293 | 7772 | 7254 | 9110 | 8957 |
| 6 | 9636 | 10335 | 10508 | 10559 | 11064 |  | 10188 | 8257 | 8891 | 10859 | 9939 |
| 7 | 8207 | 8096 | 8358 | 8804 | 9179 | 9114 | 10064 | 7510 | 7904 | 8483 | 9341 |
| 8 | 8257 | 8151 | 8515 | 8115 | 8327 | 8830 | 8342 | 7237 | 7512 | 8433 | 8364 |
| 9 | 7300 | 7185 | 7317 | 7020 | 7847 | 7243 | 7691 | 6036 | 5859 | 7083 | 7227 |
| 10 | 7828 | 8235 | 8202 | 8102 | 8108 | 7737 | 7790 | 7665 | 6967 | 7379 | 7408 |
| 11 | 8630 | 8153 | 8258 | 8388 | 8420 | 8562 | 8031 | 7164 | 6760 | 7994 | 7401 |
| 12 | 8087 | 7957 | 7827 | 7600 | 7224 | 7542 | 7804 | 6645 | 6288 | 7887 | 7733 |
| 13 | 8511 | 8712 | 8659 | 8610 | 8470 | 8554 | 8738 | 8290 | 7708 | 8672 | 8580 |
| **Mean** | **8339** | **8312** | **8431** | **8398** | **8582** | **8349** | **8505** | **7333** | **7303** | **8377** | **8233** |
| **SD** | **762** | **913** | **986** | **1147** | **1093** | **909** | **990** | **876** | **1427** | **1139** | **956** |

**S8 Table:**
