# Supplementary material for: The effects of intensified training on resting metabolic rate (RMR), body composition and performance in trained cyclists
Source: PLoS One. 2018 Feb 14;13(2):e0191644. doi: 10.1371/journal.pone.0191644 (PMC5812577; doi:10.1371/journal.pone.0191644)
Supplement: S9 Table — Data are presented as individual values for each time point, and group mean ± SD. (DOCX) [file pone.0191644.s010.docx]

| **Relative RMR (cal.kg.FFM.day^-1^)** | | | | **Relative RMR (kJ. kg.FFM.day^-1^)** | | |
| --- | --- | --- | --- | --- | --- | --- |
| **Training Block** | **Baseline** | **Loading 2** | **Recovery 2** | **Baseline** | **Loading 2** | **Recovery 2** |
| **Participant** | **Day 1** | **Day 26** | **Day 40** | **Day 1** | **Day 26** | **Day 40** |
| 1 | 25.28 | 22.64 | 25.28 | 105.7 | 94.6 | 105.7 |
| 2 | 28.93 | 25.47 | 28.65 | 120.9 | 106.5 | 119.7 |
| 3 | 28.52 | 26.46 | 28.62 | 119.2 | 110.6 | 119.6 |
| 4 | 32.30 | 25.53 | 29.31 | 135.0 | 106.7 | 122.5 |
| 5 | 32.05 | 26.48 | 30.75 | 133.9 | 110.7 | 128.6 |
| 6 | 32.44 | 29.70 | 33.07 | 135.6 | 124.2 | 138.3 |
| 7 | 26.61 | 24.80 | 30.72 | 111.2 | 103.7 | 128.4 |
| 8 | 29.57 | 25.82 | 29.75 | 123.6 | 107.9 | 124.4 |
| 9 | 26.13 | 21.01 | 24.99 | 109.2 | 87.8 | 104.5 |
| 10 | 27.02 | 26.20 | 25.04 | 113.0 | 109.5 | 104.7 |
| 11 | 29.90 | 24.91 | 26.15 | 125.0 | 104.1 | 109.3 |
| 12 | 29.28 | 24.33 | 27.56 | 122.4 | 101.7 | 115.2 |
| 13 | 31.95 | 30.74 | 30.91 | 133.6 | 128.5 | 129.2 |
| **Mean** | **29.2** | **25.7** | **28.5** | **122.2** | **107.4** | **119.2** |
| **SD** | **2.5** | **2.5** | **2.6** | **10.3** | **10.6** | **10.8** |

**S9 Table:**
