# Supplementary material for: The effects of intensified training on resting metabolic rate (RMR), body composition and performance in trained cyclists
Source: PLoS One. 2018 Feb 14;13(2):e0191644. doi: 10.1371/journal.pone.0191644 (PMC5812577; doi:10.1371/journal.pone.0191644)
Supplement: S10 Table — Data are presented as individual values for each time point, and group mean ± SD. (DOCX) [file pone.0191644.s011.docx]

|  | **Minute ventilation [VE_(STPD)_]** | | | | | | | | | | |
| --- | --- | --- | --- | --- | --- | --- | --- | --- | --- | --- | --- |
| **Training Block** | **Baseline** | **Build** | | **Loading 1** | | | **Loading 2** | | | **Recovery 1** | **Recovery 2** |
| **Participant** | **Day 1** | **Day 9** | **Day 12** | **Day 15** | **Day 17** | **Day 19** | **Day 22** | **Day 26** | **Day 29** | **Day 33** | **Day 40** |
| 1 | 5.51 | 5.55 | 5.36 | 4.99 | 5.52 | 5.69 | 5.67 | 4.95 | 4.95 | 6.01 | 6.09 |
| 2 | 5.35 | 5.45 | 5.57 | 5.46 | 5.43 | 5.49 | 5.70 | 5.02 | 4.90 | 6.29 | 6.25 |
| 3 | 6.94 | 7.63 | 7.53 | 7.69 | 7.72 | 7.47 | 7.20 | 6.63 | 8.13 | 7.42 | 7.15 |
| 4 | 5.29 | 5.04 | 4.95 | 4.74 | 5.11 | 5.01 | 4.85 | 4.43 | 4.51 | 4.99 | 4.80 |
| 5 | 6.06 | 5.84 | 5.60 | 5.44 | 5.68 | 5.49 | 5.16 | 5.31 | 4.77 | 5.97 | 5.97 |
| 6 | 7.03 | 7.62 | 7.24 | 7.07 | 7.96 | 4.59 | 8.32 | 6.23 | 7.19 | 8.66 | 7.78 |
| 7 | 6.13 | 5.53 | 5.74 | 6.45 | 6.48 | 6.79 | 7.05 | 5.22 | 5.71 | 6.38 | 6.86 |
| 8 | 6.42 | 6.41 | 6.68 | 6.00 | 6.34 | 6.94 | 6.42 | 5.79 | 5.88 | 5.98 | 6.40 |
| 9 | 5.42 | 5.56 | 5.45 | 5.32 | 6.43 | 6.26 | 5.93 | 5.29 | 4.95 | 5.88 | 5.55 |
| 10 | 6.22 | 5.98 | 6.21 | 6.13 | 6.02 | 5.88 | 6.50 | 6.65 | 6.02 | 6.66 | 6.80 |
| 11 | 7.22 | 6.89 | 7.08 | 5.99 | 7.15 | 7.33 | 6.91 | 6.16 | 6.03 | 6.85 | 6.38 |
| 12 | 5.40 | 5.43 | 5.72 | 5.26 | 4.66 | 5.46 | 5.38 | 4.63 | 4.08 | 5.38 | 4.94 |
| 13 | 5.67 | 6.34 | 6.24 | 5.99 | 5.80 | 6.16 | 6.14 | 6.00 | 6.05 | 6.61 | 6.40 |
| **Mean** | **6.05** | **6.10** | **6.11** | **5.89** | **6.18** | **6.04** | **6.25** | **5.56** | **5.63** | **6.39** | **6.26** |
| **SD** | **0.68** | **0.84** | **0.81** | **0.83** | **0.98** | **0.89** | **0.96** | **0.73** | **1.12** | **0.93** | **0.83** |

**S10 Table:**
