# Supplementary material for: The effects of intensified training on resting metabolic rate (RMR), body composition and performance in trained cyclists
Source: PLoS One. 2018 Feb 14;13(2):e0191644. doi: 10.1371/journal.pone.0191644 (PMC5812577; doi:10.1371/journal.pone.0191644)
Supplement: S11 Table — Data are presented as individual values for each time point, and group mean ± SD. (DOCX) [file pone.0191644.s012.docx]

**S11 Table:**

|  | **Body Mass (kg)** | | | **Fat mass (kg)** | | | **Fat-free mass (kg)** | | |
| --- | --- | --- | --- | --- | --- | --- | --- | --- | --- |
| **Training Block** | **Baseline** | **Loading 2** | **Recovery 2** | **Baseline** | **Loading 2** | **Recovery 2** | **Baseline** | **Loading 2** | **Recovery 2** |
| **Participant** | **Day 1** | **Day 26** | **Day 40** | **Day 1** | **Day 26** | **Day 40** | **Day 1** | **Day 26** | **Day 40** |
| 1 | 82.8 | 79.5 | 80.5 | 12.1 | 9.5 | 8.5 | 71.6 | 71.0 | 72.8 |
| 2 | 74.1 | 72.8 | 74.2 | 7.0 | 6.0 | 5.7 | 67.8 | 63.5 | 64.9 |
| 3 | 94.5 | 94.5 | 92.1 | 11.1 | 8.2 | 8.7 | 83.9 | 81.9 | 79.7 |
| 4 | 66 | 66 | 65.9 | 9.4 | 8.7 | 8.9 | 57.3 | 58.3 | 57.7 |
| 5 | 80 | 78.2 | 77.6 | 10.6 | 9.1 | 8.9 | 69.9 | 70.2 | 69.7 |
| 6 | 87.8 | 86.5 | 86.4 | 17.4 | 14.6 | 15.0 | 71.1 | 71.6 | 71.9 |
| 7 | 85.8 | 82.7 | 82.4 | 12.9 | 11.0 | 10.6 | 73.8 | 72.4 | 72.8 |
| 8 | 84.2 | 84.7 | 84.7 | 14.0 | 12.8 | 13.3 | 71.0 | 67.1 | 67.3 |
| 9 | 82 | 81.9 | 81.2 | 11.9 | 9.5 | 8.2 | 70.8 | 68.7 | 69.2 |
| 10 | 77.1 | 76.9 | 76.7 | 8.7 | 7.8 | 6.9 | 69.3 | 70.0 | 70.8 |
| 11 | 74.7 | 73.5 | 72.2 | 6.8 | 5.9 | 5.6 | 69.0 | 68.8 | 67.7 |
| 12 | 74.3 | 72 | 74.2 | 9.1 | 7.7 | 8.0 | 66.1 | 65.3 | 67.1 |
| 13 | 73.5 | 73.3 | 74.8 | 10.7 | 9.8 | 9.4 | 63.7 | 64.5 | 66.4 |
| **Mean** | **79.8** | **78.7** | **78.7** | **10.9** | **9.3** | **9.1** | **69.6** | **68.7** | **69.1** |
| **SD** | **7.5** | **7.5** | **6.9** | **2.9** | **2.5** | **2.7** | **6.0** | **5.6** | **5.1** |
