# Supplementary material for: The effects of intensified training on resting metabolic rate (RMR), body composition and performance in trained cyclists
Source: PLoS One. 2018 Feb 14;13(2):e0191644. doi: 10.1371/journal.pone.0191644 (PMC5812577; doi:10.1371/journal.pone.0191644)
Supplement: S12a-d Tables — Data are presented as individual values for each time point, and group mean ± SD. (DOCX) [file pone.0191644.s013.docx]

|  | **Total Energy Intake (mJ.day^-1^)** | | | | | | | | | | |
| --- | --- | --- | --- | --- | --- | --- | --- | --- | --- | --- | --- |
| **Training Block** | **Baseline** | **Build** | | **Loading 1** | | | **Loading 2** | | | **Recovery 1** | **Recovery 2** |
| **Participant** | **Day 1** | **Day 9** | **Day 12** | **Day 15** | **Day 17** | **Day 19** | **Day 22** | **Day 26** | **Day 29** | **Day 33** | **Day 40** |
| 1 | 15255.4 | 17857.7 | 16256.1 | 17904.0 | 15769.6 | 18551.2 | 18132.8 | 19451.0 | 18156.1 | 20415.5 | 17262.1 |
| 2 | 25777.5 | 17206.7 | 29461.9 | 17021.0 | 16947.4 | 18953.5 | 16609.9 | 20424.1 | 19775.2 | 19569.7 | 16271.3 |
| 3 | 14975.8 | 14343.1 | 14053.1 | 18038.4 | 18054.9 | 25614.6 | 25687.8 | 24232.3 | 24631.6 | 15146.2 | 21178.0 |
| 4 | 11779.9 | 10141.0 | 10643.7 | 11725.1 | 11098.1 | 15286.7 | 12984.2 | 13832.9 | 16641.9 | 11859.2 | 10404.7 |
| 5 | 13957.3 | 11322.0 | 13873.6 | 12864.8 | 12753.2 | 11186.7 | 15304.9 | 13559.3 | 16054.8 | 13395.2 | 11085.2 |
| 6 | 16092.3 | 15764.5 | 12758.1 | 18627.5 | 15400.6 | 16145.8 | 15053.4 | 12904.4 | 13197.4 | 13787.3 | 12236.7 |
| 7 | 12054.4 | 13790.0 | 11682.7 | 13081.4 | 14054.6 | 11982.7 | 11612.8 | 8547.7 | 12289.7 | 9517.8 | 9186.0 |
| 8 | 14619.9 | 12657.8 | 20278.9 | 10626.7 | 29627.7 | 17854.9 | 11507.6 | 13006.6 | 11184.8 | 11362.2 | 10528.0 |
| 9 | 14446.7 | 18711.6 | 19166.1 | 20930.1 | 25182.8 | 25098.5 | 21936.1 | 24034.5 | 25113.8 | 18901.8 | 24258.6 |
| 10 | 14714.0 | 19755.1 | 16108.4 | 18546.4 | 14742.3 | 16801.7 | 20598.7 | 18326.5 | 20234.7 | 15304.5 | 12906.0 |
| 11 | 19336.6 | 20587.6 | 20969.6 | 20222.5 | 18851.8 | 18856.8 | 21949.0 | 21075.2 | 28281.7 | 18019.2 | 16331.3 |
| 12 | 15576.8 | 18541.8 | 18471.3 | 16812.9 | 15971.0 | 15758.6 | 17285.9 | 17109.1 | 15692.7 | 16966.8 | 16580.8 |
| 13 | 13027.1 | 14733.2 | 14745.0 | 13778.5 | 14625.5 | 14305.2 | 10831.7 | 12849.4 | 15350.2 | 14153.8 | 14640.4 |
| **Mean** | 15508.7 | 15800.9 | 16805.3 | 16167.6 | 17160.0 | 17415.1 | 16884.2 | 16873.3 | 18200.3 | 15261.5 | 14836.1 |
| **SD** | 3625.7 | 3291.7 | 4997.8 | 3355.0 | 5066.5 | 4276.2 | 4642.7 | 4838.7 | 5228.4 | 3361.0 | 4443.5 |

**S12a Table:**

**S12b Table:**

|  | **Carbohydrate (g)** | | | | | | | | | | |
| --- | --- | --- | --- | --- | --- | --- | --- | --- | --- | --- | --- |
| **Training Block** | **Baseline** | **Build** | | **Loading 1** | | | **Loading 2** | | | **Recovery 1** | **Recovery 2** |
| **Participant** | **Day 1** | **Day 9** | **Day 12** | **Day 15** | **Day 17** | **Day 19** | **Day 22** | **Day 26** | **Day 29** | **Day 33** | **Day 40** |
| 1 | 484.4 | 535.9 | 625.3 | 630.4 | 570.4 | 563.8 | 602.2 | 613.4 | 532.4 | 561.5 | 528.8 |
| 2 | 617.8 | 481.5 | 947.1 | 525.4 | 561.3 | 536.1 | 494.3 | 518.0 | 602.9 | 579.8 | 453.0 |
| 3 | 397.2 | 382.8 | 361.1 | 461.6 | 423.2 | 768.0 | 734.1 | 691.0 | 695.9 | 509.2 | 637.5 |
| 4 | 354.8 | 261.8 | 297.4 | 326.0 | 346.8 | 398.5 | 379.5 | 348.4 | 493.0 | 342.8 | 326.7 |
| 5 | 392.4 | 336.7 | 442.1 | 364.3 | 375.8 | 349.1 | 507.8 | 461.5 | 535.2 | 390.2 | 359.8 |
| 6 | 404.0 | 361.6 | 353.7 | 522.9 | 377.3 | 486.0 | 305.6 | 501.4 | 332.1 | 274.3 | 355.6 |
| 7 | 220.4 | 320.0 | 193.0 | 240.1 | 308.5 | 306.0 | 286.6 | 185.5 | 235.4 | 212.9 | 222.8 |
| 8 | 374.4 | 304.0 | 642.8 | 255.4 | 677.4 | 309.2 | 357.2 | 339.2 | 318.0 | 262.4 | 248.1 |
| 9 | 98.7 | 146.9 | 162.3 | 174.3 | 185.0 | 264.5 | 194.2 | 152.2 | 307.4 | 156.6 | 179.9 |
| 10 | 465.1 | 655.7 | 443.7 | 574.7 | 408.0 | 466.9 | 687.5 | 533.6 | 700.2 | 423.9 | 353.8 |
| 11 | 534.9 | 574.6 | 575.3 | 550.3 | 537.5 | 539.8 | 633.8 | 568.7 | 892.5 | 491.4 | 455.2 |
| 12 | 536.1 | 535.2 | 564.3 | 505.1 | 489.7 | 476.5 | 572.0 | 557.6 | 552.3 | 595.6 | 429.3 |
| 13 | 315.3 | 364.7 | 410.4 | 369.8 | 351.9 | 440.0 | 332.8 | 371.0 | 432.0 | 365.1 | 347.0 |
| **Mean** | 399.7 | 404.7 | 463.0 | 423.1 | 431.8 | 454.2 | 468.3 | 449.4 | 510.0 | 397.4 | 376.7 |
| **SD** | 138.0 | 142.8 | 210.7 | 144.4 | 131.4 | 135.2 | 170.8 | 161.3 | 187.1 | 144.6 | 125.7 |

**S12c Table:**

|  | **Fat (g)** | | | | | | | | | | |
| --- | --- | --- | --- | --- | --- | --- | --- | --- | --- | --- | --- |
| **Training Block** | **Baseline** | **Build** | | **Loading 1** | | | **Loading 2** | | | **Recovery 1** | **Recovery 2** |
| **Participant** | **Day 1** | **Day 9** | **Day 12** | **Day 15** | **Day 17** | **Day 19** | **Day 22** | **Day 26** | **Day 29** | **Day 33** | **Day 40** |
| 1 | 95.8 | 120.8 | 65.6 | 113.3 | 78.8 | 124.0 | 123.9 | 127.1 | 120.0 | 169.7 | 131.7 |
| 2 | 278.6 | 180.3 | 215.0 | 142.3 | 115.1 | 181.7 | 148.0 | 215.4 | 174.7 | 149.2 | 152.5 |
| 3 | 152.7 | 130.7 | 123.4 | 138.8 | 149.1 | 168.6 | 199.1 | 157.1 | 195.5 | 99.4 | 164.7 |
| 4 | 87.5 | 74.7 | 90.2 | 104.1 | 81.7 | 153.5 | 104.4 | 157.4 | 139.3 | 94.4 | 80.7 |
| 5 | 103.1 | 73.3 | 88.7 | 107.6 | 103.0 | 80.0 | 107.2 | 84.6 | 98.6 | 99.2 | 71.6 |
| 6 | 154.2 | 172.4 | 110.5 | 196.9 | 161.4 | 138.1 | 158.2 | 74.4 | 129.7 | 166.6 | 106.1 |
| 7 | 146.6 | 140.2 | 137.7 | 139.6 | 134.9 | 104.7 | 103.4 | 92.3 | 150.3 | 93.5 | 88.3 |
| 8 | 146.0 | 123.3 | 130.3 | 98.6 | 297.5 | 245.8 | 90.2 | 120.1 | 94.5 | 105.2 | 92.3 |
| 9 | 276.1 | 380.4 | 360.6 | 399.6 | 472.6 | 439.4 | 417.2 | 468.9 | 422.7 | 344.1 | 426.4 |
| 10 | 121.2 | 137.5 | 127.9 | 151.8 | 130.2 | 143.7 | 150.0 | 147.6 | 126.8 | 126.4 | 101.5 |
| 11 | 164.8 | 175.3 | 186.2 | 168.5 | 148.2 | 149.9 | 186.6 | 169.7 | 197.6 | 151.3 | 137.8 |
| 12 | 108.3 | 171.0 | 153.5 | 142.9 | 130.6 | 130.4 | 134.1 | 133.7 | 105.6 | 115.9 | 185.0 |
| 13 | 141.7 | 152.0 | 130.1 | 123.3 | 142.3 | 118.9 | 94.8 | 107.9 | 135.8 | 116.6 | 166.9 |
| **Mean** | 152.0 | 156.3 | 147.7 | 155.9 | 165.0 | 167.6 | 155.2 | 158.2 | 160.9 | 140.9 | 146.6 |
| **SD** | 60.8 | 75.7 | 75.3 | 78.2 | 107.0 | 91.0 | 85.9 | 101.0 | 85.5 | 66.8 | 91.7 |

**S12d Table:**

|  | **Protein (g)** | | | | | | | | | | |
| --- | --- | --- | --- | --- | --- | --- | --- | --- | --- | --- | --- |
| **Training Block** | **Baseline** | **Build** | | **Loading 1** | | | **Loading 2** | | | **Recovery 1** | **Recovery 2** |
| **Participant** | **Day 1** | **Day 9** | **Day 12** | **Day 15** | **Day 17** | **Day 19** | **Day 22** | **Day 26** | **Day 29** | **Day 33** | **Day 40** |
| 1 | 180.5 | 228.9 | 168.6 | 153.4 | 167.9 | 233.9 | 180.9 | 233.5 | 213.5 | 240.6 | 183.4 |
| 2 | 256.0 | 123.5 | 167.7 | 148.8 | 154.2 | 161.7 | 139.8 | 194.8 | 155.6 | 162.1 | 151.0 |
| 3 | 138.3 | 142.2 | 183.0 | 285.2 | 290.9 | 314.9 | 272.5 | 369.7 | 311.3 | 144.2 | 230.2 |
| 4 | 126.5 | 144.7 | 126.1 | 132.8 | 122.3 | 156.8 | 152.5 | 119.8 | 176.3 | 142.6 | 104.5 |
| 5 | 162.9 | 121.5 | 145.5 | 123.7 | 120.7 | 117.9 | 122.1 | 115.5 | 153.0 | 136.5 | 91.8 |
| 6 | 152.3 | 160.6 | 144.1 | 128.9 | 168.7 | 150.6 | 182.3 | 98.0 | 130.4 | 147.8 | 101.6 |
| 7 | 158.6 | 174.3 | 181.3 | 202.4 | 203.7 | 162.9 | 153.9 | 103.6 | 153.0 | 133.4 | 112.9 |
| 8 | 155.5 | 163.0 | 245.8 | 136.2 | 384.9 | 179.8 | 110.7 | 146.3 | 118.9 | 163.3 | 150.3 |
| 9 | 123.8 | 131.5 | 157.8 | 164.6 | 237.9 | 228.8 | 160.8 | 210.9 | 217.1 | 180.5 | 279.7 |
| 10 | 129.2 | 188.9 | 209.3 | 167.4 | 163.5 | 200.5 | 185.9 | 207.3 | 175.6 | 190.6 | 176.0 |
| 11 | 220.6 | 226.4 | 217.8 | 235.5 | 224.9 | 217.0 | 225.2 | 268.2 | 308.4 | 208.2 | 184.3 |
| 12 | 145.2 | 177.6 | 184.0 | 161.5 | 157.8 | 160.5 | 146.7 | 153.8 | 133.2 | 146.1 | 133.7 |
| 13 | 121.8 | 146.5 | 146.2 | 150.1 | 177.9 | 128.0 | 89.1 | 131.2 | 146.9 | 194.8 | 132.0 |
| **Mean** | 159.3 | 163.8 | 175.2 | 168.5 | 198.1 | 185.6 | 163.3 | 181.0 | 184.1 | 168.5 | 156.3 |
| **SD** | 39.7 | 35.0 | 33.7 | 46.7 | 73.3 | 53.1 | 48.3 | 78.0 | 63.0 | 32.4 | 54.4 |
