# Supplementary material for: The effects of intensified training on resting metabolic rate (RMR), body composition and performance in trained cyclists
Source: PLoS One. 2018 Feb 14;13(2):e0191644. doi: 10.1371/journal.pone.0191644 (PMC5812577; doi:10.1371/journal.pone.0191644)
Supplement: S13a-d Tables — Data are presented as individual values for each time point, and group mean ± SD. (DOCX) [file pone.0191644.s014.docx]

|  | **How hungry do you feel?** | | | | | | | | | | |
| --- | --- | --- | --- | --- | --- | --- | --- | --- | --- | --- | --- |
| **Training Block** | **Baseline** | **Build** | | **Loading 1** | | | **Loading 2** | | | **Recovery 1** | **Recovery 2** |
| **Participant** | **Day 1** | **Day 9** | **Day 12** | **Day 15** | **Day 17** | **Day 19** | **Day 22** | **Day 26** | **Day 29** | **Day 33** | **Day 40** |
| 1 | 7 | 5 | 7 | 8 | 8 | 8 | 7 | 9 | 10 | 8 | 9 |
| 2 |  | 7 | 4 | 7 | 7 | 6 | 6 | 8 | 6 | 8 | 7 |
| 3 | 8 | 7 | 9 | 9 | 9 | 8 | 8 | 9 | 3 | 8 | 9 |
| 4 | 7 | 7 | 7 | 6 | 8 | 7 | 7 | 7 | 8 | 7 | 7 |
| 5 | 6 | 6 | 6 | 6 | 7 | 7 | 7 | 7 | 8 | 8 | 8 |
| 6 | 7 | 7 | 5 | 6 | 5 | 6 | 5 | 6 | 6 | 5 | 5 |
| 7 | 3 | 3 | 4 | 2 | 4 | 5 | 3 | 3 | 3 | 3 | 4 |
| 8 | 8 | 8 | 8 | 8 | 8 | 8 | 8 | 9 | 8 | 8 | 9 |
| 9 | 6 | 7 | 7 | 7 | 7 | 7 | 8 | 7 | 8 | 7 | 8 |
| 10 | 8 | 4 | 8 | 6 | 8 | 8 | 4 | 8 | 8 | 8 | 8 |
| 11 | 8 | 7 | 5 | 7 | 7 | 8 | 8 | 8 | 7 | 9 | 9 |
| 12 | 8 | 8 | 8 | 6 | 5 | 8 | 7 | 8 | 9 | 6 | 7 |
| 13 | 7 | 6 | 5 | 7 | 6 | 6 | 6 | 7 | 7 | 7 | 7 |
| **Mean** | 6.9 | 6.3 | 6.4 | 6.5 | 6.8 | 7.1 | 6.5 | 7.4 | 7.0 | 7.1 | 7.5 |
| **SD** | 1.4 | 1.5 | 1.7 | 1.7 | 1.5 | 1.0 | 1.6 | 1.6 | 2.1 | 1.6 | 1.6 |

**S13a Table:**

**S13b Table:**

|  | **How full do you feel?** | | | | | | | | | | |
| --- | --- | --- | --- | --- | --- | --- | --- | --- | --- | --- | --- |
| **Training Block** | **Baseline** | **Build** | | **Loading 1** | | | **Loading 2** | | | **Recovery 1** | **Recovery 2** |
| **Participant** | **Day 1** | **Day 9** | **Day 12** | **Day 15** | **Day 17** | **Day 19** | **Day 22** | **Day 26** | **Day 29** | **Day 33** | **Day 40** |
| 1 | 3 | 4 | 2 | 3 | 3 | 3 | 3 | 2 | 1 | 1 | 2 |
| 2 |  | 3 | 6 | 3 | 4 | 4 | 4 | 3 | 3 | 2 | 2 |
| 3 | 1 | 1 | 1 | 1 | 1 | 1 | 1 | 1 | 1 | 3 | 1 |
| 4 | 4 | 5 | 3 | 4 | 3 | 4 | 4 | 4 | 2 | 4 | 4 |
| 5 | 4 | 3 | 5 | 4 | 4 | 4 | 4 | 4 | 3 | 3 | 3 |
| 6 | 4 | 5 | 3 | 4 | 4 | 5 | 5 | 1 | 6 | 5 | 5 |
| 7 | 1 | 4 | 2 | 3 | 2 | 2 | 3 | 3 | 3 | 3 | 2 |
| 8 | 1 | 1 | 1 | 1 | 1 | 1 | 1 | 1 | 1 | 1 | 1 |
| 9 | 4 | 4 | 3 | 5 | 4 | 4 | 3 | 4 | 3 | 2 | 2 |
| 10 | 1 | 4 | 3 | 4 | 4 | 3 | 4 | 3 | 2 | 3 | 3 |
| 11 | 1 | 1 | 3 | 3 | 1 | 2 | 3 | 2 | 4 | 2 | 1 |
| 12 | 1 | 2 | 2 | 3 | 4 | 3 | 3 | 3 | 2 | 5 | 7 |
| 13 | 2 | 4 | 4 | 4 | 5 | 5 | 5 | 4 | 4 | 4 | 4 |
| **Mean** | 2.3 | 3.2 | 2.9 | 3.2 | 3.1 | 3.2 | 3.3 | 2.7 | 2.7 | 2.9 | 2.8 |
| **SD** | 1.4 | 1.5 | 1.4 | 1.2 | 1.4 | 1.3 | 1.3 | 1.2 | 1.4 | 1.3 | 1.8 |

**S13c Table:**

|  | **How satisfied do you feel?** | | | | | | | | | | |
| --- | --- | --- | --- | --- | --- | --- | --- | --- | --- | --- | --- |
| **Training Block** | **Baseline** | **Build** | | **Loading 1** | | | **Loading 2** | | | **Recovery 1** | **Recovery 2** |
| **Participant** | **Day 1** | **Day 9** | **Day 12** | **Day 15** | **Day 17** | **Day 19** | **Day 22** | **Day 26** | **Day 29** | **Day 33** | **Day 40** |
| 1 | 3 | 4 | 2 | 2 | 2 | 3 | 3 | 2 | 1 | 1 | 2 |
| 2 |  | 3 | 4 | 4 | 4 | 4 | 4 | 3 | 3 | 2 | 2 |
| 3 | 2 | 3 | 3 | 1 | 1 | 2 | 1 | 2 | 2 | 3 | 2 |
| 4 | 4 | 4 | 3 | 4 | 4 | 4 | 4 | 3 | 2 | 3 | 4 |
| 5 | 4 | 4 | 4 | 4 | 4 | 3 | 3 | 4 | 2 | 3 | 3 |
| 6 | 5 | 5 | 6 | 7 | 5 | 4 | 5 | 4 | 7 | 5 | 5 |
| 7 | 5 | 6 | 5 | 4 | 3 | 4 | 3 | 4 | 3 | 4 | 3 |
| 8 | 3 | 1 | 1 | 1 | 1 | 1 | 1 | 1 | 1 | 1 | 1 |
| 9 | 4 | 4 | 4 | 4 | 5 | 3 | 3 | 4 | 3 | 2 | 2 |
| 10 | 1 | 4 | 2 | 6 | 4 | 2 | 3 | 3 | 2 | 3 | 2 |
| 11 | 3 | 4 | 3 | 3 | 3 | 2 | 2 | 2 | 3 | 1 | 1 |
| 12 | 2 | 2 | 2 | 3 | 4 | 3 | 3 | 2 | 2 | 5 | 4 |
| 13 | 5 | 4 | 4 | 4 | 4 | 5 | 4 | 4 | 4 | 4 | 4 |
| **Mean** | 3.4 | 3.7 | 3.3 | 3.6 | 3.4 | 3.1 | 3.0 | 2.9 | 2.7 | 2.8 | 2.7 |
| **SD** | 1.3 | 1.3 | 1.4 | 1.7 | 1.3 | 1.1 | 1.2 | 1.0 | 1.5 | 1.4 | 1.3 |

**S13d Table:**

|  | **How much do you think you could eat now?** | | | | | | | | | | |
| --- | --- | --- | --- | --- | --- | --- | --- | --- | --- | --- | --- |
| **Training Block** | **Baseline** | **Build** | | **Loading 1** | | | **Loading 2** | | | **Recovery 1** | **Recovery 2** |
| **Participant** | **Day 1** | **Day 9** | **Day 12** | **Day 15** | **Day 17** | **Day 19** | **Day 22** | **Day 26** | **Day 29** | **Day 33** | **Day 40** |
| 1 | 10 | 7 | 9 | 8 | 8 | 8 | 9 | 10 | 10 | 9 | 9 |
| 2 |  | 8 | 7 | 8 | 9 | 8 | 8 | 9 | 8 | 8 | 9 |
| 3 | 10 | 10 | 10 | 10 | 10 | 10 | 10 | 10 | 3 | 10 | 10 |
| 4 | 8 | 7 | 7 | 7 | 8 | 7 | 7 | 7 | 8 | 7 | 7 |
| 5 | 8 | 6 | 7 | 7 | 7 | 7 | 7 | 7 | 8 | 8 | 8 |
| 6 | 6 | 7 | 4 | 5 | 5 | 5 | 4 | 6 | 7 | 5 | 5 |
| 7 | 8 | 7 | 7 | 7 | 8 | 8 | 6 | 7 | 7 | 6 | 7 |
| 8 | 8 | 8 | 8 | 9 | 8 | 8 | 8 | 9 | 8 | 8 | 8 |
| 9 | 8 | 6 | 7 | 7 | 7 | 8 | 8 | 7 | 8 | 8 | 8 |
| 10 | 10 | 7 | 7 | 6 | 7 | 8 | 7 | 2 | 7 | 8 | 9 |
| 11 | 7 | 7 | 6 | 6 | 8 | 7 | 8 | 9 | 7 | 9 | 10 |
| 12 | 9 | 9 | 9 | 7 | 6 | 8 | 8 | 9 | 9 | 8 | 9 |
| 13 | 8 | 7 | 7 | 7 | 7 | 6 | 5 | 7 | 7 | 7 | 7 |
| **Mean** | 8.3 | 7.4 | 7.3 | 7.2 | 7.5 | 7.5 | 7.3 | 7.6 | 7.5 | 7.8 | 8.2 |
| **SD** | 1.2 | 1.1 | 1.5 | 1.3 | 1.3 | 1.2 | 1.6 | 2.1 | 1.6 | 1.3 | 1.4 |
