# Supplementary material for: The effects of intensified training on resting metabolic rate (RMR), body composition and performance in trained cyclists
Source: PLoS One. 2018 Feb 14;13(2):e0191644. doi: 10.1371/journal.pone.0191644 (PMC5812577; doi:10.1371/journal.pone.0191644)
Supplement: S14a-b Tables — Data are presented as individual values for each time point, and group mean ± SD. (DOCX) [file pone.0191644.s015.docx]

**S14a Table:**

|  | **Leptin PRE and POST warm-up (pg.mL^-1^)** | | | | | | | | | | | | | | | | | | | |  |
| --- | --- | --- | --- | --- | --- | --- | --- | --- | --- | --- | --- | --- | --- | --- | --- | --- | --- | --- | --- | --- | --- |
| **Training Block** | **Baseline** | | **Build** | | | **Loading 1** | | | | | **Loading 2** | | | | **Recovery 1** | | | | **Recovery 2** | |  |
| **Participant** | **Day 1** | | | **Day 9** | | | **Day 15** | | **Day 19** | | **Day 22** | | **Day 26** | | | **Day 33** | | **Day 40** | | |  |
|  | **PRE** | **POST** | | **PRE** | **POST** | | **PRE** | **POST** | **PRE** | **POST** | **PRE** | **POST** | **PRE** | **POST** | | **PRE** | **POST** | **PRE** | | **POST** | |
| 1 | 1378 | 1739 | | 457 | 551 | | 308 | 571 | 485 | 498 | 337 | 378 | 174 | 165 | | 439 | 567 | 288 | | 308 | |
| 2 | 1054 | 895 | | 474 | 478 | | 459 | 396 | 195 | 231 | 157 | 181 | 136 | 146 | | 198 | 240 | 421 | | 460 | |
| 3 | 900 | 1019 | | 495 | 502 | | 225 | 260 | 146 | 192 | 296 | 269 |  |  | | 365 |  | 559 | |  | |
| 4 | 734 | 806 | | 1233 | 1317 | | 564 | 593 | 816 | 817 | 899 | 924 | 696 | 819 | | 1044 | 1205 | 1142 | | 1353 | |
| 5 | 569 | 510 | | 426 | 483 | | 276 | 296 | 156 | 242 | 352 | 392 | 179 | 319 | | 194 |  | 430 | | 484 | |
| 6 | 1582 | 2278 | | 1180 | 1711 | | 510 | 652 | 897 | 1070 | 1431 | 1554 | 1300 | 1154 | | 1307 | 1225 | 2317 | | 2093 | |
| 7 | 1196 | 1202 | | 560 | 687 | | 753 | 722 | 606 | 632 | 578 | 575 | 978 | 903 | | 1349 | 1325 | 823 | | 681 | |
| 8 | 1556 | 1591 | | 1266 | 1572 | | 688 | 882 | 832 | 899 | 1185 | 1607 | 1112 | 1265 | | 1958 | 1775 | 1132 | | 1393 | |
| 9 | 357 | 343 | | 212 | 241 | | 375 | 365 | 209 | 194 | 308 | 359 | 288 | 223 | | 334 | 386 | 270 | | 277 | |
| 10 | 495 | 588 | | 246 | 157 | | 87 | 131 | 193 | 129 | 88 | 179 | 82 | 108 | | 167 | 183 | 130 | | 162 | |
| 11 | 466 | 571 | | 470 | 395 | | 229 | 273 | 146 | 195 | 261 | 271 | 265 | 314 | | 149 | 274 | 150 | | 170 | |
| 12 | 550 | 532 | | 270 | 321 | | 200 | 398 | 134 |  | 208 | 274 | 126 | 178 | | 234 | 296 | 548 | | 486 | |
| 13 | 637 |  | | 383 | 302 | | 320 | 395 | 487 | 536 | 158 | 244 | 393 | 455 | | 525 | 533 | 497 | | 542 | |
| **Mean** | **883** | **1006** | | **590** | **670** | | **384** | **456** | **408** | **470** | **481** | **554** | **477** | **504** | | **636** | **728** | **670** | | **701** | |
| **SD** | **428.8** | **592.5** | | **377.2** | **517.2** | | **199.0** | **213.0** | **294.9** | **323.4** | **425.3** | **496.2** | **431.0** | **417.6** | | **583.9** | **551.0** | **592.7** | | **597.3** | |

**S14b Table:**

|  | **fT3 PRE and POST warm-up (pmol.L^-1^)** | | | | | | | | | | | | | | | | | | | | |
| --- | --- | --- | --- | --- | --- | --- | --- | --- | --- | --- | --- | --- | --- | --- | --- | --- | --- | --- | --- | --- | --- |
| **Training Block** | **Baseline** | | | **Build** | | **Loading 1** | | | | | **Loading 2** | | | | **Recovery 1** | | | | **Recovery 2** | | |
| **Participant** | **Day 1** | | **Day 9** | | | | **Day 15** | | **Day 19** | | **Day 22** | | **Day 26** | | | **Day 33** | | **Day 40** | | |  |
|  | **PRE** | **POST** | **PRE** | | **POST** | | **PRE** | **POST** | **PRE** | **POST** | **PRE** | **POST** | **PRE** | **POST** | | **PRE** | **POST** | **PRE** | | **POST** |  |
| 1 | 5.15 | 5.04 | 4.77 | | 4.54 | | 4.69 | 4.69 | 4.09 | 4.10 | 4.21 | 4.11 | 4.28 | 4.21 | | 4.22 | 4.10 | 4.59 | | 4.43 |  |
| 2 | 5.79 | 5.56 | 5.39 | | 5.23 | | 5.69 | 6.20 | 5.32 | 5.44 | 4.40 | 5.00 | 5.37 | 5.43 | | 5.43 | 5.78 | 5.03 | | 4.91 |  |
| 3 | 4.81 | 4.58 | 5.19 | | 5.71 | | 4.18 | 4.12 | 4.43 | 4.66 | 4.24 | 4.29 |  |  | | 4.42 |  | 4.92 | |  |  |
| 4 | 5.99 | 6.95 | 5.74 | | 5.50 | | 5.88 | 5.55 | 5.81 | 5.71 | 5.12 | 5.20 | 5.73 | 5.83 | | 5.86 | 5.68 | 4.99 | | 5.11 |  |
| 5 | 6.27 | 6.56 | 5.47 | | 5.65 | | 5.89 | 5.64 | 5.49 | 3.44 | 5.11 | 5.23 | 5.07 | 5.26 | | 5.45 |  | 5.90 | | 5.95 |  |
| 6 | 5.00 | 4.79 | 5.09 | | 4.90 | | 5.32 | 5.12 | 4.99 | 4.92 | 4.59 | 4.71 | 5.05 | 5.16 | | 4.95 | 4.90 | 5.26 | | 4.88 |  |
| 7 | 5.22 | 5.17 | 5.19 | | 5.47 | | 4.55 | 4.83 | 4.43 | 4.36 | 5.06 | 5.49 | 4.32 | 4.37 | | 5.19 | 5.47 | 4.64 | | 4.80 |  |
| 8 | 5.22 | 5.40 | 5.04 | | 4.88 | | 4.73 | 4.77 | 4.85 | 4.95 | 4.88 | 4.92 | 4.70 | 4.70 | | 5.20 | 5.03 | 5.10 | | 5.34 |  |
| 9 | 3.49 | 3.67 | 3.26 | | 3.32 | | 3.45 | 3.38 | 3.31 | 5.54 | 3.03 | 3.40 | 2.82 | 3.04 | | 2.91 | 2.80 | 3.07 | | 3.07 |  |
| 10 | 4.75 | 4.65 | 4.21 | | 4.02 | | 4.28 | 4.15 | 4.17 | 4.12 | 4.10 | 3.95 | 4.00 | 3.93 | | 3.90 | 3.91 | 3.91 | | 3.90 |  |
| 11 | 4.85 | 4.75 | 4.35 | | 4.59 | | 4.36 | 4.27 | 4.22 | 4.30 | 4.16 | 3.99 | 4.13 | 4.03 | | 4.15 | 4.17 | 3.97 | | 4.17 |  |
| 12 | 5.01 | 4.79 | 4.33 | | 4.43 | | 4.41 | 4.38 | 4.71 |  | 4.09 | 4.09 | 4.45 | 4.37 | | 4.68 | 4.45 | 5.10 | | 5.20 |  |
| 13 |  |  | 5.37 | | 5.34 | | 5.30 | 5.10 | 4.93 | 4.75 | 4.40 | 4.46 | 4.47 | 4.58 | | 4.91 | 4.61 | 4.78 | | 4.87 |  |
| **Mean** | **5.13** | **5.16** | **4.88** | | **4.89** | | **4.83** | **4.78** | **4.67** | **4.69** | **4.41** | **4.53** | **4.53** | **4.58** | | **4.71** | **4.63** | **4.71** | | **4.72** |  |
| **SD** | **0.71** | **0.89** | **0.68** | | **0.70** | | **0.74** | **0.75** | **0.67** | **0.67** | **0.57** | **0.62** | **0.75** | **0.76** | | **0.79** | **0.88** | **0.72** | | **0.75** |  |
