# Supplementary material for: The effects of intensified training on resting metabolic rate (RMR), body composition and performance in trained cyclists
Source: PLoS One. 2018 Feb 14;13(2):e0191644. doi: 10.1371/journal.pone.0191644 (PMC5812577; doi:10.1371/journal.pone.0191644)
Supplement: S15a-b Tables — Data are presented as individual values for each time point, and group mean ± SD. (DOCX) [file pone.0191644.s016.docx]

|  | **RMSSD (ms)** | | | | | | | | | | |
| --- | --- | --- | --- | --- | --- | --- | --- | --- | --- | --- | --- |
| **Training Block** | **Baseline** | **Build** | | **Loading 1** | | | **Loading 2** | | | **Recovery 1** | **Recovery 2** |
| **Participant** | **Day 1** | **Day 9** | **Day 12** | **Day 15** | **Day 17** | **Day 19** | **Day 22** | **Day 26** | **Day 29** | **Day 33** | **Day 40** |
| 1 | 84.6 | 39.2 | 70.5 | 77.7 | 40.3 |  |  | 123.5 | 42.37 | 10.2 | 20.8 |
| 2 | 95.4 | 62.5 | 82.4 | 56.2 | 55.1 | 104.7 | 82.3 | 109.3 | 56.3 | 10.4 | 49.5 |
| 3 | 93.7 | 27.9 | 62.8 | 86.5 |  | 170.9 |  | 109.5 | 53.2 | 50.11 | 94.7 |
| 4 | 122.7 | 64.6 | 91.6 | 96.2 | 57.6 | 66 | 52.6 | 118.6 | 69.5 | 73.4 | 103.4 |
| 5 | 82 | 16 | 82.1 | 92.8 |  | 52.5 | 104.7 | 110.7 | 72.4 | 95.1 | 91.6 |
| 6 | 101.9 | 26.6 | 69.8 | 24.9 | 54 | 48.1 | 43.5 | 35.46 | 61.5 | 80.1 | 114.9 |
| 7 | 95.2 | 47.2 | 84.3 | 85.3 |  | 150.7 | 45.4 | 115 | 23.1 | 5.6 | 107.5 |
| 8 | 65.8 | 29.3 | 79.8 | 81.4 | 43.7 | 114.6 | 50.2 | 120.2 |  | 20.3 | 88.9 |
| 9 | 87.1 | 51.9 | 98.8 | 27.3 | 83.7 | 66.3 | 57.4 |  |  | 8.1 |  |
| 10 | 70.6 | 109 | 77.1 | 26.2 | 28.2 |  | 61.8 | 64.4 | 10.8 | 27.8 | 91.2 |
| 11 | 76.3 | 74.4 | 100.9 | 37.2 | 26.4 |  | 75.2 | 56.3 | 8.6 | 22.9 | 80.2 |
| 12 | 29.9 | 125.4 | 71 | 22.5 | 113.3 | 176.9 | 132.2 | 61.2 | 8.7 | 19.2 |  |
| 13 | 67.9 | 153.7 | 49.33 | 27.7 | 202.2 | 64.4 |  | 67.7 | 7.7 | 7.7 | 148 |
| **Mean** | **82.5** | **63.7** | **78.5** | **57.1** | **70.5** | **101.5** | **70.5** | **91.0** | **37.7** | **33.1** | **90.1** |
| **SD** | **22.2** | **42.0** | **14.3** | **30.0** | **53.1** | **49.7** | **28.8** | **31.2** | **26.3** | **31.0** | **33.2** |

**S15a Table:**

**S15b Table:**

|  | **LnRMSSD** | | | | | | | | | | |
| --- | --- | --- | --- | --- | --- | --- | --- | --- | --- | --- | --- |
| **Training Block** | **Baseline** | **Build** | | **Loading 1** | | | **Loading 2** | | | **Recovery 1** | **Recovery 2** |
| **Participant** | **Day 1** | **Day 9** | **Day 12** | **Day 15** | **Day 17** | **Day 19** | **Day 22** | **Day 26** | **Day 29** | **Day 33** | **Day 40** |
| 1 | 4.438 | 3.669 | 4.256 | 4.353 | 3.696 |  |  | 4.816 | 3.746 | 2.322 | 3.035 |
| 2 | 4.558 | 4.135 | 4.412 | 4.029 | 4.009 | 4.651 | 4.410 | 4.694 | 4.031 | 2.342 | 3.902 |
| 3 | 4.540 | 3.329 | 4.140 | 4.460 |  | 5.141 |  | 4.696 | 3.974 | 3.914 | 4.551 |
| 4 | 4.810 | 4.168 | 4.517 | 4.566 | 4.054 | 4.190 | 3.963 | 4.776 | 4.241 | 4.296 | 4.639 |
| 5 | 4.407 | 2.773 | 4.408 | 4.530 |  | 3.961 | 4.651 | 4.707 | 4.282 | 4.555 | 4.517 |
| 6 | 4.624 | 3.281 | 4.246 | 3.215 | 3.989 | 3.873 | 3.773 | 3.568 | 4.119 | 4.383 | 4.744 |
| 7 | 4.556 | 3.854 | 4.434 | 4.446 |  | 5.015 | 3.816 | 4.745 | 3.140 | 1.723 | 4.677 |
| 8 | 4.187 | 3.378 | 4.380 | 4.399 | 3.777 | 4.741 | 3.916 | 4.789 |  | 3.011 | 4.488 |
| 9 | 4.467 | 3.949 | 4.593 | 3.307 | 4.427 | 4.194 | 4.050 |  |  | 2.092 |  |
| 10 | 4.257 | 4.691 | 4.345 | 3.266 | 3.339 |  | 4.124 | 4.165 | 2.380 | 3.325 | 4.513 |
| 11 | 4.335 | 4.309 | 4.614 | 3.616 | 3.273 |  | 4.320 | 4.031 | 2.152 | 3.131 | 4.385 |
| 12 | 3.398 | 4.832 | 4.263 | 3.114 | 4.730 | 5.176 | 4.884 | 4.114 | 2.163 | 2.955 |  |
| 13 | 4.218 | 5.035 | 3.899 | 3.321 | 5.309 | 4.165 |  | 4.215 | 2.041 | 2.041 | 4.997 |
| **Mean** | **4.369** | **3.954** | **4.347** | **3.894** | **4.060** | **4.511** | **4.191** | **4.443** | **3.297** | **3.084** | **4.404** |
| **SD** | **0.340** | **0.668** | **0.193** | **0.590** | **0.624** | **0.494** | **0.369** | **0.408** | **0.937** | **0.964** | **0.527** |
