# Supplementary material for: The effects of intensified training on resting metabolic rate (RMR), body composition and performance in trained cyclists
Source: PLoS One. 2018 Feb 14;13(2):e0191644. doi: 10.1371/journal.pone.0191644 (PMC5812577; doi:10.1371/journal.pone.0191644)
Supplement: S16a-e Tables — Data are presented as individual values for each time point, and group mean ± SD. (DOCX) [file pone.0191644.s017.docx]

|  | **15s power output (W)** | | | | | | | | | | | | | | |
| --- | --- | --- | --- | --- | --- | --- | --- | --- | --- | --- | --- | --- | --- | --- | --- |
| **Training Block** | **Baseline** | | | **Build** | | | **Loading 1** | | | **Loading 2** | | | | **Rec 1** | **Rec 2** |
| **Participant** | **Day 1** | **Day 5** | **Day 9** | | **Day 12** | **Day 15** | | **Day 17** | **Day 19** | **Day 22** | **Day 24** | **Day 26** | **Day 29** | **Day 33** | **Day 40** |
| 1 |  | 699.3 | 781.0 | | 729.1 | 627.9 | | 639.0 | 660.0 | 628.6 | 580.3 |  | 650.0 | 626.6 | 612.7 |
| 2 |  | 933.3 | 781.3 | | 877.7 | 846.7 | | 801.6 | 769.3 | 808.9 | 694.2 | 772.3 | 740.3 | 819.7 | 906.6 |
| 3 | 968.3 | 699.3 |  | | 822.1 | 786.9 | | 822.1 | 810.7 | 738.9 | 720.1 | 610.7 |  | 626.9 | 676.1 |
| 4 | 761.6 | 773.2 | 832.1 | | 782.5 | 747.7 | | 737.5 | 734.9 | 762.6 | 714.7 |  | 721.3 | 770.2 | 759.1 |
| 5 | 641.3 | 688.7 | 779.1 | | 791.5 | 750.0 | | 721.3 | 647.3 | 663.2 | 626.5 | 637.5 | 700.1 | 666.7 | 736.7 |
| 6 | 939.4 | 1065.2 | 997.3 | | 1031.9 | 1024.9 | | 975.6 | 890.2 | 899.6 | 942.1 | 943.7 | 983.8 | 900.3 | 924.2 |
| 7 | 827.6 | 738.6 | 595.4 | | 673.1 | 705.3 | | 601.8 | 780.1 | 612.2 | 769.3 | 613.6 | 749.5 | 778.2 | 772.5 |
| 8 |  | 1009.6 | 853.1 | | 841.6 | 932.6 | | 724.6 | 748.3 | 662.5 | 553.0 |  | 643.1 | 662.8 | 756.0 |
| 9 | 827.7 | 729.8 | 753.5 | | 763.3 | 736.3 | | 708.1 | 700.3 | 629.5 | 540.8 |  | 685.4 |  | 743.9 |
| 10 | 818.5 | 762.8 | 752.0 | |  | 723.9 | | 757.2 | 649.7 | 663.2 | 645.8 |  | 631.7 | 717.6 | 745.7 |
| 11 | 934.6 | 881.7 | 895.6 | | 871.4 | 841.3 | | 814.7 | 849.5 | 773.7 | 740.3 | 525.6 | 804.5 | 823.4 | 860.3 |
| 12 |  | 723.4 | 716.2 | | 672.8 | 630.2 | | 639.0 | 641.7 | 685.7 | 512.1 | 759.6 | 619.5 | 643.2 | 647.1 |
| 13 | 964.0 | 843.3 | 827.4 | | 754.8 | 780.2 | | 843.2 | 738.9 | 763.8 | 718.9 |  | 724.6 |  | 711.1 |
| **Mean** | **853.7** | **811.4** | **797.0** | | **801.0** | **779.5** | | **752.7** | **740.1** | **714.8** | **673.7** | **694.7** | **721.2** | **730.5** | **757.8** |
| **SD** | **109.2** | **125.2** | **98.6** | | **99.2** | **111.7** | | **100.6** | **79.8** | **84.6** | **116.2** | **140.1** | **99.2** | **93.4** | **92.9** |

**S16a Table:**

**S16b Table:**

|  | **4000m TT power output (W)** | | | | | | | | | | | | | | |
| --- | --- | --- | --- | --- | --- | --- | --- | --- | --- | --- | --- | --- | --- | --- | --- |
| **Training Block** | **Baseline** | | | **Build** | | | **Loading 1** | | | **Loading 2** | | | | **Rec 1** | **Rec 2** |
| **Participant** | **Day 1** | **Day 5** | **Day 9** | | **Day 12** | **Day 15** | | **Day 17** | **Day 19** | **Day 22** | **Day 24** | **Day 26** | **Day 29** | **Day 33** | **Day 40** |
| 1 | 375.18 | 290.81 | 354.96 | | 355.25 | 336.28 | | 332.52 | 371.94 | 341.36 | 340.10 |  | 355.40 | 370.17 | 382.39 |
| 2 |  | 382.81 | 342.56 | | 377.95 | 342.59 | | 391.57 | 374.61 | 401.44 | 365.14 | 376.31 | 337.42 | 388.84 | 440.82 |
| 3 | 394.11 | 387.52 |  | | 389.98 | 392.14 | | 402.85 | 403.49 | 387.86 | 408.88 | 380.40 |  | 376.20 | 408.51 |
| 4 | 334.78 | 349.73 | 345.70 | | 342.22 | 349.25 | | 345.95 | 357.41 | 346.36 | 335.29 |  | 325.84 | 340.43 | 324.12 |
| 5 | 339.53 | 351.97 | 346.24 | | 328.91 | 339.95 | | 342.33 | 326.86 | 318.98 | 331.12 | 338.66 | 331.44 | 353.13 | 362.54 |
| 6 | 350.70 | 379.26 | 391.60 | | 402.95 | 410.16 | | 402.73 | 398.86 | 406.57 | 408.88 | 413.30 | 404.18 | 422.26 | 415.03 |
| 7 | 345.34 | 359.52 | 338.49 | | 353.49 | 342.24 | | 327.36 | 338.83 | 360.43 | 358.37 | 356.22 | 375.78 | 381.69 | 376.11 |
| 8 | 374.13 | 383.48 | 372.13 | | 386.86 | 362.68 | | 308.45 | 354.27 | 358.15 | 366.57 |  | 390.58 | 405.85 | 409.74 |
| 9 | 373.35 | 365.66 | 383.86 | | 370.96 | 379.83 | | 374.25 | 385.90 | 380.41 | 371.33 |  | 365.20 | 377.78 | 389.95 |
| 10 | 428.94 | 423.98 | 413.15 | | 413.35 | 404.01 | | 426.25 | 398.65 | 412.89 | 412.41 |  | 394.86 | 419.88 | 432.58 |
| 11 | 369.72 | 391.46 | 403.55 | | 387.78 | 373.60 | | 380.72 | 388.15 | 370.21 | 380.08 | 368.46 | 376.79 | 394.85 | 390.30 |
| 12 |  | 321.38 | 341.58 | | 346.56 | 347.64 | | 367.29 | 359.89 | 369.99 | 297.64 | 297.59 | 341.58 | 364.28 | 348.14 |
| 13 | 327.12 | 360.67 | 347.14 | | 358.24 | 358.41 | | 356.53 | 349.38 | 332.23 | 356.91 |  | 331.42 |  | 367.43 |
| **Mean** | **364.8** | **365.2** | **365.1** | | **370.3** | **364.5** | | **366.1** | **369.9** | **368.2** | **364.1** | **361.6** | **360.9** | **382.9** | **388.3** |
| **SD** | **29.7** | **33.4** | **26.7** | | **25.3** | **25.3** | | **34.5** | **24.4** | **29.1** | **33.7** | **36.4** | **27.5** | **25.0** | **33.3** |

**S16c Table:**

|  | **4000m TT heart rate (bpm)** | | | | | | | | | | | | | | |
| --- | --- | --- | --- | --- | --- | --- | --- | --- | --- | --- | --- | --- | --- | --- | --- |
| **Training Block** | **Baseline** | | | **Build** | | | **Loading 1** | | | **Loading 2** | | | | **Rec 1** | **Rec 2** |
| **Participant** | **Day 1** | **Day 5** | **Day 9** | | **Day 12** | **Day 15** | | **Day 17** | **Day 19** | **Day 22** | **Day 24** | **Day 26** | **Day 29** | **Day 33** | **Day 40** |
| 1 | 189 | 173 | 181 | | 180 | 176 | | 173 | 181 | 179 | 176 | 151 | 179 | 181 | 184 |
| 2 | 193 | 191 | 187 | | 189 | 184 | | 187 | 185 | 187 | 181 | 184 | 172 | 185 | 190 |
| 3 | 179 | 179 | 175 | | 172 | 175 | | 174 | 171 | 171 | 171 | 168 |  | 175 | 174 |
| 4 | 179 | 181 | 184 | | 181 | 178 | | 180 | 181 | 178 | 173 | 178 | 168 | 181 | 177 |
| 5 | 173 | 173 | 168 | | 168 | 170 | | 168 | 165 | 164 | 167 | 168 | 165 | 168 | 177 |
| 6 | 181 | 182 | 176 | | 186 | 177 | | 176 | 172 | 179 | 180 | 172 | 180 | 183 | 188 |
| 7 | 168 | 165 | 171 | | 171 | 165 | | 165 | 168 | 170 | 172 | 174 | 171 | 173 | 175 |
| 8 | 191 | 196 | 192 | | 191 | 195 | | 172 | 189 | 197 | 186 | 181 | 187 | 192 | 192 |
| 9 | 180 | 176 | 173 | | 175 | 174 | | 173 | 169 | 171 | 169 | 172 | 166 | 180 | 174 |
| 10 | 189 | 187 | 188 | | 184 | 188 | | 191 | 184 | 184 | 181 | 179 | 178 | 188 | 189 |
| 11 | 165 | 163 | 162 | | 169 | 171 | | 160 | 157 | 155 | 158 | 156 | 153 | 161 | 161 |
| 12 | 177 | 165 | 169 | | 167 | 167 | | 171 | 172 | 172 | 164 | 162 | 168 | 168 | 172 |
| 13 | 184 | 186 | 170 | | 178 | 181 | | 176 | 177 | 173 | 178 | 172 | 171 | 182 | 183 |
| **Mean** | **180.6** | **178.2** | **176.6** | | **177.8** | **177.0** | | **174.3** | **174.7** | **175.4** | **173.5** | **170.5** | **171.5** | **178.2** | **179.7** |
| **SD** | **8.6** | **10.3** | **9.1** | | **8.2** | **8.4** | | **8.3** | **9.1** | **10.6** | **7.8** | **9.6** | **8.8** | **8.8** | **8.9** |

**S16d Table:**

|  | **4000m TT blood lactate (mmol.L^-1^)** | | | | | | | | | | | | | | |
| --- | --- | --- | --- | --- | --- | --- | --- | --- | --- | --- | --- | --- | --- | --- | --- |
| **Training Block** | **Baseline** | | | **Build** | | | **Loading 1** | | | **Loading 2** | | | | **Rec 1** | **Rec 2** |
| **Participant** | **Day 1** | **Day 5** | **Day 9** | | **Day 12** | **Day 15** | | **Day 17** | **Day 19** | **Day 22** | **Day 24** | **Day 26** | **Day 29** | **Day 33** | **Day 40** |
| 1 | 12.4 | 5.4 | 7.6 | |  | 10.3 | | 7 | 8 | 12.8 | 8.2 | 2.9 | 11.4 | 8.3 | 13.1 |
| 2 | 18.8 | 20.2 | 11 | | 17.1 | 12.8 | | 12.9 | 16 | 14.9 | 6.7 | 21.1 | 5 | 14.7 | 20.6 |
| 3 | 12.5 | 19.9 | 19.1 | | 13.8 | 8.5 | | 13 | 14.3 | 12.5 | 9 | 18 |  | 8.6 | 18.8 |
| 4 | 12.2 | 16.8 | 15.7 | | 18.1 | 16.7 | | 13.3 | 17.3 | 19.1 | 12.5 | 14.6 | 10.4 | 12.7 | 14.3 |
| 5 |  | 14.4 | 13.2 | | 13 | 12.3 | | 20 | 11.8 | 13.2 | 16.6 | 11.2 | 6.6 | 14.3 | 16.3 |
| 6 | 19.5 | 19.3 | 11.1 | | 23 | 17 | | 16.3 | 18.6 | 16.8 | 13.2 | 15.9 | 16.7 | 17.1 | 22.3 |
| 7 | 12 | 11.7 | 12.9 | | 12.5 | 11.7 | | 14.1 | 13.8 | 9.4 | 10.7 | 10.8 | 16.6 | 9.7 | 12.4 |
| 8 | 10.1 | 15.2 | 15.6 | | 13.5 | 18.7 | | 4.4 | 13.3 | 14.6 | 9.4 | 6.9 | 10.9 | 20.8 | 13.1 |
| 9 |  | 7.8 | 6.6 | | 7.4 | 11.9 | | 11.9 | 7 | 8.8 | 5.5 | 7.5 | 6.2 | 9.4 | 9.7 |
| 10 | 17 | 13.1 | 11.5 | | 22.3 | 15.4 | | 16.2 | 15.8 | 22.2 | 12.4 | 22 | 15.7 | 14.2 | 16.7 |
| 11 | 9.3 | 9.8 | 9 | | 16.8 | 10.4 | | 7.6 | 18.4 | 12.5 | 10 | 8.6 | 6 | 12.7 | 11.4 |
| 12 | 9.2 | 7.7 | 7.5 | | 8.1 | 7.2 | | 7.1 | 7.6 | 10.2 | 5.8 | 2.5 | 5.4 | 7.8 | 8.6 |
| 13 |  | 16.6 | 9.2 | | 11.8 | 17.7 | | 11.4 | 11 | 10.1 | 12.8 | 13 | 10.4 | 10.4 | 15.6 |
| **Mean** | **13.3** | **13.7** | **11.5** | | **14.8** | **13.1** | | **11.9** | **13.3** | **13.6** | **10.2** | **11.9** | **10.1** | **12.4** | **14.8** |
| **SD** | **3.8** | **4.9** | **3.7** | | **4.9** | **3.7** | | **4.4** | **4.0** | **3.9** | **3.3** | **6.3** | **4.4** | **3.8** | **4.1** |

**S16e Table:**

|  | **4000m TT Rating of Perceived Exertion (Borg 1-20)** | | | | | | | | | | | | | | |
| --- | --- | --- | --- | --- | --- | --- | --- | --- | --- | --- | --- | --- | --- | --- | --- |
| **Training Block** | **Baseline** | | | **Build** | | | **Loading 1** | | | **Loading 2** | | | | **Rec 1** | **Rec 2** |
| **Participant** | **Day 1** | **Day 5** | **Day 9** | | **Day 12** | **Day 15** | | **Day 17** | **Day 19** | **Day 22** | **Day 24** | **Day 26** | **Day 29** | **Day 33** | **Day 40** |
| 1 | 19 | 17 | 15 | | 17 | 13 | | 15 | 15 | 17 | 17 | 11 | 17 | 19 | 19 |
| 2 | 18 | 18 | 17 | | 18 | 18 | | 18 | 18 | 18 | 18 | 18 | 18 | 17 | 20 |
| 3 | 20 | 19 | 18 | | 18 | 19 | | 19 | 19 | 17 | 19 | 18 |  | 19 | 19 |
| 4 |  | 19 | 19 | | 19 | 18 | | 19 | 19 | 18 | 18 | 18 | 19 | 19 | 19 |
| 5 | 20 | 19 | 19 | | 17 | 19 | | 18 | 19 | 18 | 18 | 18 | 18 | 17 | 19 |
| 6 | 19 | 18 | 16 | | 17 | 16 | | 16 | 16 | 17 | 17 | 15 | 17 | 15 | 16 |
| 7 | 14 | 18 | 17 | | 18 | 20 | | 20 | 20 | 18 | 20 | 16 | 18 | 18 | 18 |
| 8 |  | 20 | 19 | | 19 | 19 | | 15 | 18 | 18 | 17 | 17 | 18 | 19 | 19 |
| 9 | 18 | 19 | 19 | | 17 | 17 | | 17 | 18 | 18 | 17 | 19 | 17 | 15 | 19 |
| 10 | 19 | 19 | 18 | | 17 | 16 | | 15 | 15 | 14 | 18 | 17 | 16 | 16 | 19 |
| 11 |  | 18 | 18 | | 20 | 18 | | 19 | 19 | 20 | 19 | 18 | 20 | 20 | 20 |
| 12 | 19 | 19 | 19 | | 18 | 17 | | 17 | 18 | 16 | 19 | 18 | 19 | 19 | 18 |
| 13 | 20 | 19 | 19 | | 19 | 19 | | 20 | 20 | 20 | 20 | 20 | 20 | 20 | 20 |
| **Mean** | **18.6** | **18.6** | **17.9** | | **18.0** | **17.6** | | **17.5** | **18.0** | **17.6** | **18.2** | **17.2** | **18.1** | **17.9** | **18.8** |
| **SD** | **1.8** | **0.8** | **1.3** | | **1.0** | **1.9** | | **1.9** | **1.7** | **1.6** | **1.1** | **2.2** | **1.2** | **1.8** | **1.1** |
