# Supplementary material for: The effects of intensified training on resting metabolic rate (RMR), body composition and performance in trained cyclists
Source: PLoS One. 2018 Feb 14;13(2):e0191644. doi: 10.1371/journal.pone.0191644 (PMC5812577; doi:10.1371/journal.pone.0191644)
Supplement: S17 Table — Data are presented as individual values for each time point, and group mean ± SD. (DOCX) [file pone.0191644.s018.docx]

| **Multicomponent Training Distress Scale** | | | | | | | | | | |
| --- | --- | --- | --- | --- | --- | --- | --- | --- | --- | --- |
| **Participant** | **Training Block** | **Day** | **Depressed mood** | **Vigour** | **Physical signs and symptoms** | **Sleep disturbances** | **Perceived stress** | **Fatigue** | **Total mood disturbance** |  |
|  | **Baseline** | **1** |  | | | | | | |  |
| 1 |  |  | 1.00 | 0.25 | 0.33 | 0.33 | 0.50 | 0.33 | 2.75 |  |
| 2 |  |  | 0.20 | 1.50 | 0.00 | 0.67 | 0.75 | 1.33 | 4.45 |  |
| 3 |  |  | 0.20 | 1.50 | 1.00 | 0.00 | 0.50 | 0.33 | 3.53 |  |
| 4 |  |  | 0.00 | 1.00 | 1.00 | 1.00 | 1.25 | 1.67 | 5.92 |  |
| 5 |  |  | 0.40 | 1.75 | 0.33 | 0.00 | 0.50 | 1.00 | 3.98 |  |
| 6 |  |  | 1.20 | 2.25 | 2.00 | 0.67 | 0.50 | 1.67 | 8.28 |  |
| 7 |  |  | 1.20 | 1.75 | 1.00 | 0.67 | 1.00 | 1.67 | 7.28 |  |
| 8 |  |  | 0.00 | 0.25 | 1.67 | 2.00 | 0.00 | 0.67 | 4.58 |  |
| 9 |  |  | 1.80 | 1.50 | 0.00 | 0.33 | 1.00 | 0.67 | 5.30 |  |
| 10 |  |  | 0.00 | 1.25 | 1.67 | 0.67 | 0.25 | 2.00 | 5.83 |  |
| 11 |  |  | 0.20 | 1.50 | 2.67 | 0.00 | 0.25 | 1.00 | 5.62 |  |
| 12 |  |  | 2.60 | 1.50 | 2.33 | 1.00 | 1.75 | 1.33 | 10.52 |  |
| 13 |  |  | 0.00 | 1.00 | 1.33 | 0.00 | 0.00 | 1.00 | 3.33 |  |
| **Mean** |  | | **0.7** | **1.3** | **1.2** | **0.6** | **0.6** | **1.1** | **5.5** |  |
| **SD** |  |  | **0.8** | **0.6** | **0.9** | **0.6** | **0.5** | **0.5** | **2.2** |  |

**S17 Table:**

| **Participant** | **Training Block** | **Day** | **Depressed mood** | **Vigour** | **Physical signs and symptoms** | **Sleep disturbances** | **Perceived stress** | **Fatigue** | **Total mood disturbance** |
| --- | --- | --- | --- | --- | --- | --- | --- | --- | --- |
|  | **Baseline** | **5** |  | | | | | | |
| 1 |  |  | 0.20 | 1.50 | 0.33 | 1.00 | 0.00 | 1.00 | 4.03 |
| 2 |  |  | 0.00 | 2.00 | 0.00 | 1.00 | 0.00 | 0.67 | 3.67 |
| 3 |  |  | 0.00 | 1.00 | 0.00 | 0.00 | 0.25 | 0.33 | 1.58 |
| 4 |  |  | 0.00 | 1.25 | 2.67 | 1.67 | 0.75 | 1.67 | 8.00 |
| 5 |  |  | 0.40 | 1.75 | 0.67 | 0.67 | 0.25 | 1.00 | 4.73 |
| 6 |  |  | 0.20 | 2.00 | 1.00 | 0.33 | 0.00 | 1.67 | 5.20 |
| 7 |  |  | 1.00 | 1.75 | 0.67 | 0.33 | 1.25 | 1.33 | 6.33 |
| 8 |  |  | 0.00 | 0.25 | 0.00 | 0.33 | 0.00 | 0.00 | 0.58 |
| 9 |  |  | 0.40 | 1.00 | 0.00 | 0.33 | 0.25 | 1.67 | 3.65 |
| 10 |  |  | 1.20 | 2.25 | 0.67 | 0.00 | 0.25 | 0.67 | 5.03 |
| 11 |  |  | 0.00 | 1.50 | 2.00 | 0.00 | 0.25 | 1.67 | 5.42 |
| 12 |  |  | 0.80 | 1.50 | 3.00 | 1.33 | 1.00 | 1.67 | 9.30 |
| 13 |  |  | 0.00 | 1.25 | 1.33 | 0.33 | 0.00 | 0.67 | 3.58 |
| **Mean** |  | | **0.3** | **1.5** | **0.9** | **0.6** | **0.3** | **1.1** | **4.7** |
| **SD** |  |  | **0.4** | **0.5** | **1.0** | **0.5** | **0.4** | **0.6** | **2.3** |

| **Participant** | **Training Block** | **Day** | **Depressed mood** | **Vigour** | **Physical signs and symptoms** | **Sleep disturbances** | **Perceived stress** | **Fatigue** | **Total mood disturbance** |
| --- | --- | --- | --- | --- | --- | --- | --- | --- | --- |
|  | **Build** | **9** |  | | | | | | |
| 1 |  |  | 0.80 | 0.50 | 0.00 | 0.33 | 0.25 | 1.00 | 2.88 |
| 2 |  |  | 0.00 | 1.75 | 0.00 | 0.67 | 0.00 | 0.33 | 2.75 |
| 3 |  |  | 0.00 | 1.25 | 0.67 | 0.00 | 0.00 | 1.00 | 2.92 |
| 4 |  |  | 0.00 | 2.00 | 3.00 | 1.67 | 0.75 | 3.33 | 10.75 |
| 5 |  |  | 0.00 | 1.75 | 0.67 | 0.00 | 0.25 | 0.67 | 3.33 |
| 6 |  |  | 0.60 | 2.25 | 0.67 | 0.33 | 0.25 | 1.00 | 5.10 |
| 7 |  |  | 1.00 | 1.75 | 1.67 | 0.67 | 1.00 | 1.00 | 7.08 |
| 8 |  |  | 0.00 | 0.50 | 0.00 | 0.00 | 0.00 | 1.00 | 1.50 |
| 9 |  |  | 0.80 | 1.25 | 0.33 | 0.33 | 0.00 | 1.67 | 4.38 |
| 10 |  |  | 2.00 | 4.00 | 1.00 | 3.33 | 1.00 | 3.33 | 14.67 |
| 11 |  |  | 0.20 | 1.00 | 0.67 | 0.33 | 0.75 | 1.00 | 3.95 |
| 12 |  |  | 1.20 | 2.00 | 2.33 | 1.00 | 0.75 | 1.33 | 8.62 |
| 13 |  |  | 0.00 | 1.25 | 1.67 | 0.00 | 0.00 | 0.67 | 3.58 |
| **Mean** |  | | **0.5** | **1.6** | **1.0** | **0.7** | **0.4** | **1.3** | **5.5** |
| **SD** |  |  | **0.6** | **0.9** | **0.9** | **0.9** | **0.4** | **0.9** | **3.8** |

| **Participant** | **Training Block** | **Day** | **Depressed mood** | **Vigour** | **Physical signs and symptoms** | **Sleep disturbances** | **Perceived stress** | **Fatigue** | **Total mood disturbance** |
| --- | --- | --- | --- | --- | --- | --- | --- | --- | --- |
|  | **Build** | **12** |  | | | | | | |
| 1 |  |  | 0.00 | 1.75 | 1.00 | 2.33 | 0.50 | 2.33 | 7.92 |
| 2 |  |  | 0.00 | 1.75 | 0.67 | 0.67 | 0.00 | 1.00 | 4.08 |
| 3 |  |  | 0.00 | 0.75 | 1.00 | 0.00 | 0.25 | 1.00 | 3.00 |
| 4 |  |  | 0.80 | 2.50 | 2.00 | 0.67 | 0.50 | 1.67 | 8.13 |
| 5 |  |  | 0.60 | 1.75 | 1.33 | 0.00 | 0.25 | 1.00 | 4.93 |
| 6 |  |  | 0.40 | 2.75 | 0.67 | 0.67 | 0.25 | 1.00 | 5.73 |
| 7 |  |  | 1.40 | 2.50 | 1.67 | 2.00 | 1.25 | 1.67 | 10.48 |
| 8 |  |  | 0.00 | 1.50 | 0.33 | 0.00 | 0.00 | 1.67 | 3.50 |
| 9 |  |  | 0.40 | 1.00 | 1.00 | 0.33 | 0.25 | 1.67 | 4.65 |
| 10 |  |  | 0.40 | 2.00 | 1.00 | 0.00 | 0.75 | 1.33 | 5.48 |
| 11 |  |  | 0.00 | 1.25 | 0.00 | 0.00 | 0.50 | 0.00 | 1.75 |
| 12 |  |  | 0.60 | 2.00 | 3.67 | 1.00 | 1.00 | 3.00 | 11.27 |
| 13 |  |  | 0.00 | 2.00 | 1.33 | 0.00 | 0.00 | 1.33 | 4.67 |
| **Mean** |  | | **0.4** | **1.8** | **1.2** | **0.6** | **0.4** | **1.4** | **5.8** |
| **SD** |  |  | **0.4** | **0.6** | **0.9** | **0.8** | **0.4** | **0.7** | **2.9** |

| **Participant** | **Training Block** | **Day** | **Depressed mood** | **Vigour** | **Physical signs and symptoms** | **Sleep disturbances** | **Perceived stress** | **Fatigue** | **Total mood disturbance** |
| --- | --- | --- | --- | --- | --- | --- | --- | --- | --- |
|  | **Loading 1** | **15** |  | | | | | | |
| 1 |  |  | 0.00 | 1.00 | 1.00 | 1.33 | 0.50 | 1.33 | 5.17 |
| 2 |  |  | 0.00 | 2.00 | 0.67 | 0.67 | 0.25 | 1.33 | 4.92 |
| 3 |  |  | 0.00 | 1.00 | 1.67 | 0.33 | 0.00 | 1.00 | 4.00 |
| 4 |  |  | 0.80 | 2.00 | 3.00 | 0.67 | 1.00 | 1.67 | 9.13 |
| 5 |  |  | 0.00 | 1.75 | 1.00 | 0.00 | 0.50 | 1.00 | 4.25 |
| 6 |  |  | 0.80 | 2.75 | 1.00 | 0.33 | 0.25 | 1.33 | 6.47 |
| 7 |  |  | 1.00 | 2.00 | 2.00 | 1.00 | 1.50 | 1.33 | 8.83 |
| 8 |  |  | 0.00 | 1.75 | 1.00 | 1.00 | 0.00 | 1.33 | 5.08 |
| 9 |  |  | 0.80 | 1.25 | 0.67 | 0.00 | 0.00 | 1.00 | 3.72 |
| 10 |  |  | 0.80 | 1.00 | 0.67 | 1.33 | 0.50 | 1.00 | 5.30 |
| 11 |  |  | 0.00 | 1.50 | 2.00 | 0.00 | 0.50 | 1.00 | 5.00 |
| 12 |  |  | 0.40 | 2.00 | 2.00 | 0.33 | 0.50 | 2.33 | 7.57 |
| 13 |  |  | 0.00 | 1.00 | 1.00 | 0.00 | 0.00 | 0.33 | 2.33 |
| **Mean** |  | | **0.4** | **1.6** | **1.4** | **0.5** | **0.4** | **1.2** | **5.5** |
| **SD** |  |  | **0.4** | **0.5** | **0.7** | **0.5** | **0.4** | **0.5** | **2.0** |

| **Participant** | **Training Block** | **Day** | **Depressed mood** | **Vigour** | **Physical signs and symptoms** | **Sleep disturbances** | **Perceived stress** | **Fatigue** | **Total mood disturbance** |
| --- | --- | --- | --- | --- | --- | --- | --- | --- | --- |
|  | **Loading 1** | **17** |  | | | | | | |
| 1 |  |  | 0.00 | 0.75 | 1.33 | 0.33 | 0.00 | 1.33 | 3.75 |
| 2 |  |  | 0.00 | 2.50 | 0.67 | 0.67 | 0.00 | 1.33 | 5.17 |
| 3 |  |  | 0.00 | 0.50 | 1.67 | 0.00 | 0.00 | 1.00 | 3.17 |
| 4 |  |  | 0.00 | 1.75 | 3.00 | 0.00 | 1.00 | 2.33 | 8.08 |
| 5 |  |  | 1.00 | 2.00 | 1.33 | 0.00 | 0.50 | 1.67 | 6.50 |
| 6 |  |  | 0.60 | 2.25 | 0.67 | 0.00 | 0.25 | 0.67 | 4.43 |
| 7 |  |  | 0.80 | 2.25 | 1.67 | 1.00 | 1.00 | 1.67 | 8.38 |
| 8 |  |  | 0.00 | 2.00 | 1.33 | 1.00 | 0.00 | 1.67 | 6.00 |
| 9 |  |  | 0.80 | 1.25 | 0.67 | 0.33 | 0.25 | 1.00 | 4.30 |
| 10 |  |  | 1.60 | 1.75 | 0.00 | 0.00 | 1.00 | 2.00 | 6.35 |
| 11 |  |  | 0.00 | 1.00 | 0.67 | 0.33 | 0.75 | 0.67 | 3.42 |
| 12 |  |  | 1.80 | 2.50 | 2.00 | 0.33 | 1.25 | 3.00 | 10.88 |
| 13 |  |  | 0.00 | 2.00 | 2.00 | 0.33 | 0.00 | 1.33 | 5.67 |
| **Mean** |  | | **0.5** | **1.7** | **1.3** | **0.3** | **0.5** | **1.5** | **5.9** |
| **SD** |  |  | **0.7** | **0.7** | **0.8** | **0.4** | **0.5** | **0.7** | **2.2** |

| **Participant** | **Training Block** | **Day** | **Depressed mood** | **Vigour** | **Physical signs and symptoms** | **Sleep disturbances** | **Perceived stress** | **Fatigue** | **Total mood disturbance** |
| --- | --- | --- | --- | --- | --- | --- | --- | --- | --- |
|  | **Loading 1** | **19** |  | | | | | | |
| 1 |  |  | 0.00 | 0.25 | 2.33 | 2.33 | 0.00 | 1.67 | 6.58 |
| 2 |  |  | 0.00 | 2.50 | 1.00 | 0.67 | 0.00 | 1.33 | 5.50 |
| 3 |  |  | 0.00 | 0.50 | 2.00 | 0.00 | 0.00 | 1.00 | 3.50 |
| 4 |  |  | 0.80 | 1.75 | 3.00 | 0.33 | 1.75 | 2.33 | 9.97 |
| 5 |  |  | 0.00 | 1.75 | 2.00 | 0.00 | 0.00 | 1.33 | 5.08 |
| 6 |  |  | 0.20 | 2.75 | 1.67 | 0.67 | 0.00 | 2.00 | 7.28 |
| 7 |  |  | 1.60 | 2.75 | 2.67 | 1.00 | 1.50 | 2.33 | 11.85 |
| 8 |  |  | 0.00 | 2.25 | 1.33 | 0.00 | 0.00 | 1.33 | 4.92 |
| 9 |  |  | 0.80 | 1.25 | 1.00 | 0.33 | 0.25 | 1.33 | 4.97 |
| 10 |  |  | 0.80 | 2.00 | 0.33 | 1.00 | 0.50 | 2.33 | 6.97 |
| 11 |  |  | 0.00 | 1.50 | 2.00 | 0.00 | 0.50 | 1.33 | 5.33 |
| 12 |  |  | 0.60 | 2.50 | 1.67 | 1.33 | 0.50 | 2.67 | 9.27 |
| 13 |  |  | 0.00 | 2.00 | 1.67 | 0.00 | 0.00 | 1.00 | 4.67 |
| **Mean** |  | | **0.4** | **1.8** | **1.7** | **0.6** | **0.4** | **1.7** | **6.6** |
| **SD** |  |  | **0.5** | **0.8** | **0.7** | **0.7** | **0.6** | **0.6** | **2.4** |

| **Participant** | **Training Block** | **Day** | **Depressed mood** | **Vigour** | **Physical signs and symptoms** | **Sleep disturbances** | **Perceived stress** | **Fatigue** | **Total mood disturbance** |
| --- | --- | --- | --- | --- | --- | --- | --- | --- | --- |
|  | **Loading 2** | **22** |  | | | | | | |
| 1 |  |  | 0.00 | 0.00 | 0.67 | 0.00 | 0.00 | 0.00 | 0.67 |
| 2 |  |  | 0.00 | 2.75 | 1.33 | 0.67 | 0.25 | 1.33 | 6.33 |
| 3 |  |  | 0.00 | 0.50 | 2.00 | 0.00 | 0.00 | 0.67 | 3.17 |
| 4 |  |  | 0.00 | 2.00 | 3.67 | 0.67 | 1.00 | 2.33 | 9.67 |
| 5 |  |  | 0.00 | 1.75 | 2.67 | 0.67 | 0.25 | 1.33 | 6.67 |
| 6 |  |  | 1.40 | 3.50 | 0.67 | 0.00 | 1.00 | 3.00 | 9.57 |
| 7 |  |  | 1.40 | 2.75 | 1.00 | 2.00 | 1.75 | 1.67 | 10.57 |
| 8 |  |  | 0.00 | 2.25 | 1.33 | 0.00 | 0.00 | 1.67 | 5.25 |
| 9 |  |  | 0.60 | 1.75 | 1.33 | 0.00 | 0.00 | 0.67 | 4.35 |
| 10 |  |  | 0.60 | 3.00 | 0.33 | 1.67 | 1.00 | 2.00 | 8.60 |
| 11 |  |  | 0.00 | 2.25 | 2.00 | 0.00 | 0.25 | 1.67 | 6.17 |
| 12 |  |  | 0.60 | 2.25 | 2.33 | 0.33 | 1.00 | 3.67 | 10.18 |
| 13 |  |  | 0.00 | 2.00 | 2.00 | 0.33 | 0.00 | 1.00 | 5.33 |
| **Mean** |  | | **0.4** | **2.1** | **1.6** | **0.5** | **0.5** | **1.6** | **6.7** |
| **SD** |  |  | **0.5** | **1.0** | **0.9** | **0.7** | **0.6** | **1.0** | **3.0** |

| **Participant** | **Training Block** | **Day** | **Depressed mood** | **Vigour** | **Physical signs and symptoms** | **Sleep disturbances** | **Perceived stress** | **Fatigue** | **Total mood disturbance** |
| --- | --- | --- | --- | --- | --- | --- | --- | --- | --- |
|  | **Loading 2** | **24** |  | | | | | | |
| 1 |  |  | 0.00 | 0.75 | 1.33 | 0.67 | 0.25 | 1.33 | 4.33 |
| 2 |  |  | 0.00 | 2.75 | 0.67 | 0.67 | 0.25 | 0.33 | 4.67 |
| 3 |  |  | 0.00 | 1.50 | 2.33 | 0.00 | 0.00 | 1.67 | 5.50 |
| 4 |  |  | 0.60 | 2.00 | 3.00 | 0.00 | 1.25 | 3.00 | 9.85 |
| 5 |  |  | 0.60 | 2.00 | 2.33 | 0.33 | 0.25 | 2.00 | 7.52 |
| 6 |  |  | 0.40 | 2.50 | 1.67 | 0.00 | 0.50 | 1.67 | 6.73 |
| 7 |  |  | 1.00 | 2.50 | 0.67 | 1.67 | 1.50 | 1.33 | 8.67 |
| 8 |  |  | 0.00 | 2.00 | 1.00 | 0.00 | 0.00 | 1.33 | 4.33 |
| 9 |  |  | 1.40 | 1.75 | 1.67 | 0.00 | 0.25 | 1.33 | 6.40 |
| 10 |  |  | 0.00 | 2.25 | 1.33 | 0.33 | 0.50 | 1.67 | 6.08 |
| 11 |  |  | 0.00 | 1.75 | 0.67 | 0.00 | 0.75 | 1.00 | 4.17 |
| 12 |  |  | 0.40 | 2.50 | 3.00 | 0.33 | 0.75 | 3.33 | 10.32 |
| 13 |  |  | 0.00 | 2.00 | 0.67 | 0.00 | 0.00 | 1.00 | 3.67 |
| **Mean** |  | | **0.3** | **2.0** | **1.6** | **0.3** | **0.5** | **1.6** | **6.3** |
| **SD** |  |  | **0.5** | **0.5** | **0.9** | **0.5** | **0.5** | **0.8** | **2.2** |

| **Participant** | **Training Block** | **Day** | **Depressed mood** | **Vigour** | **Physical signs and symptoms** | **Sleep disturbances** | **Perceived stress** | **Fatigue** | **Total mood disturbance** |
| --- | --- | --- | --- | --- | --- | --- | --- | --- | --- |
|  | **Loading 2** | **26** |  | | | | | | |
| 1 |  |  | 0.00 | 1.50 | 1.00 | 3.67 | 0.75 | 1.67 | 8.58 |
| 2 |  |  | 0.00 | 2.75 | 1.33 | 0.67 | 0.25 | 1.67 | 6.67 |
| 3 |  |  | 0.20 | 1.75 | 2.33 | 0.00 | 0.50 | 2.33 | 7.12 |
| 4 |  |  | 0.00 | 2.50 | 3.00 | 0.67 | 1.75 | 3.67 | 11.58 |
| 5 |  |  | 0.20 | 2.25 | 1.33 | 0.00 | 0.25 | 1.67 | 5.70 |
| 6 |  |  | 0.00 | 2.25 | 1.00 | 0.00 | 0.25 | 1.33 | 4.83 |
| 7 |  |  | 1.80 | 2.50 | 0.67 | 2.00 | 1.75 | 1.67 | 10.38 |
| 8 |  |  | 0.00 | 2.75 | 1.33 | 1.33 | 0.00 | 3.00 | 8.42 |
| 9 |  |  | 0.60 | 1.75 | 1.33 | 1.00 | 0.00 | 1.33 | 6.02 |
| 10 |  |  | 0.20 | 2.75 | 1.33 | 0.33 | 0.25 | 1.33 | 6.20 |
| 11 |  |  | 0.00 | 2.75 | 1.33 | 0.00 | 1.00 | 1.33 | 6.42 |
| 12 |  |  | 4.00 | 3.00 | 3.00 | 0.33 | 2.50 | 3.67 | 16.50 |
| 13 |  |  | 0.00 | 1.00 | 1.67 | 0.33 | 0.00 | 1.00 | 4.00 |
| **Mean** |  | | **0.5** | **2.3** | **1.6** | **0.8** | **0.7** | **2.0** | **7.9** |
| **SD** |  |  | **1.2** | **0.6** | **0.7** | **1.1** | **0.8** | **0.9** | **3.3** |

| **Participant** | **Training Block** | **Day** | **Depressed mood** | **Vigour** | **Physical signs and symptoms** | **Sleep disturbances** | **Perceived stress** | **Fatigue** | **Total mood disturbance** |
| --- | --- | --- | --- | --- | --- | --- | --- | --- | --- |
|  | **Loading 2** | **29** |  | | | | | | |
| 1 |  |  | 0.00 | 1.00 | 2.33 | 2.67 | 0.75 | 1.67 | 8.42 |
| 2 |  |  | 0.00 | 2.75 | 2.00 | 0.67 | 0.50 | 2.00 | 7.92 |
| 3 |  |  | 1.80 | 4.00 | 4.00 | 2.67 | 1.00 | 3.00 | 16.47 |
| 4 |  |  | 0.00 | 3.00 | 3.67 | 2.67 | 1.75 | 3.33 | 14.42 |
| 5 |  |  | 0.00 | 2.00 | 2.33 | 1.00 | 0.25 | 1.67 | 7.25 |
| 6 |  |  | 0.00 | 2.25 | 1.00 | 0.33 | 0.00 | 0.00 | 3.58 |
| 7 |  |  | 1.20 | 2.50 | 0.67 | 0.67 | 1.25 | 1.00 | 7.28 |
| 8 |  |  | 0.00 | 2.00 | 1.67 | 0.00 | 0.00 | 2.67 | 6.33 |
| 9 |  |  | 0.00 | 1.25 | 1.00 | 0.67 | 0.00 | 1.00 | 3.92 |
| 10 |  |  | 0.60 | 2.00 | 2.33 | 0.00 | 0.50 | 3.00 | 8.43 |
| 11 |  |  | 0.00 | 4.00 | 1.67 | 0.00 | 0.00 | 2.67 | 8.33 |
| 12 |  |  | 1.00 | 2.00 | 3.33 | 0.67 | 1.00 | 3.67 | 11.67 |
| 13 |  |  | 0.00 | 2.00 | 2.33 | 0.00 | 0.00 | 1.33 | 5.67 |
| **Mean** |  | | **0.4** | **2.4** | **2.2** | **0.9** | **0.5** | **2.1** | **8.4** |
| **SD** |  |  | **0.6** | **0.9** | **1.0** | **1.0** | **0.6** | **1.1** | **3.8** |

| **Participant** | **Training Block** | **Day** | **Depressed mood** | **Vigour** | **Physical signs and symptoms** | **Sleep disturbances** | **Perceived stress** | **Fatigue** | **Total mood disturbance** |
| --- | --- | --- | --- | --- | --- | --- | --- | --- | --- |
|  | **Recovery 1** | **33** |  | | | | | | |
| 1 |  |  | 0.00 | 1.75 | 0.67 | 1.33 | 0.50 | 1.33 | 5.58 |
| 2 |  |  | 0.00 | 2.50 | 1.00 | 0.67 | 0.50 | 1.00 | 5.67 |
| 3 |  |  | 0.00 | 0.75 | 0.33 | 0.00 | 0.00 | 0.67 | 1.75 |
| 4 |  |  | 0.00 | 2.50 | 2.00 | 1.00 | 1.50 | 2.67 | 9.67 |
| 5 |  |  | 0.00 | 1.25 | 1.67 | 0.00 | 0.00 | 1.00 | 3.92 |
| 6 |  |  | 0.00 | 3.50 | 0.67 | 0.67 | 0.50 | 2.00 | 7.33 |
| 7 |  |  | 0.60 | 2.25 | 0.67 | 1.33 | 1.50 | 1.33 | 7.68 |
| 8 |  |  | 0.00 | 1.75 | 0.33 | 0.00 | 0.00 | 0.67 | 2.75 |
| 9 |  |  | 0.00 | 1.00 | 0.00 | 0.33 | 0.00 | 0.67 | 2.00 |
| 10 |  |  | 0.20 | 2.25 | 0.67 | 0.33 | 0.50 | 1.33 | 5.28 |
| 11 |  |  | 0.20 | 1.00 | 0.00 | 0.00 | 0.25 | 0.00 | 1.45 |
| 12 |  |  | 0.20 | 2.00 | 1.33 | 0.33 | 0.50 | 2.33 | 6.70 |
| 13 |  |  | 0.00 | 1.25 | 1.67 | 0.00 | 0.00 | 1.00 | 3.92 |
| **Mean** |  | | **0.1** | **1.8** | **0.8** | **0.5** | **0.4** | **1.2** | **4.9** |
| **SD** |  |  | **0.2** | **0.8** | **0.6** | **0.5** | **0.5** | **0.7** | **2.5** |

| **Participant** | **Training Block** | **Day** | **Depressed mood** | **Vigour** | **Physical signs and symptoms** | **Sleep disturbances** | **Perceived stress** | **Fatigue** | **Total mood disturbance** |
| --- | --- | --- | --- | --- | --- | --- | --- | --- | --- |
|  | **Recovery 2** | **36** |  | | | | | | |
| 1 |  |  | 0.00 | 1.75 | 0.67 | 0.00 | 0.00 | 1.67 | 4.08 |
| 2 |  |  | 0.00 | 1.75 | 0.00 | 0.67 | 0.00 | 0.33 | 2.75 |
| 3 |  |  | 0.00 | 1.00 | 2.33 | 0.00 | 0.25 | 1.67 | 5.25 |
| 4 |  |  | 0.20 | 1.75 | 0.67 | 0.67 | 1.50 | 1.33 | 6.12 |
| 5 |  |  | 0.00 | 1.50 | 1.00 | 0.33 | 0.00 | 1.00 | 3.83 |
| 6 |  |  | 0.40 | 3.50 | 0.67 | 1.00 | 0.75 | 2.00 | 8.32 |
| 7 |  |  | 0.80 | 2.00 | 1.00 | 0.33 | 0.75 | 1.00 | 5.88 |
| 8 |  |  | 0.00 | 1.00 | 1.33 | 0.33 | 0.00 | 1.00 | 3.67 |
| 9 |  |  | 0.00 | 1.00 | 0.67 | 0.67 | 0.00 | 1.33 | 3.67 |
| 10 |  |  | 0.20 | 2.50 | 2.33 | 1.00 | 0.25 | 1.00 | 7.28 |
| 11 |  |  | 0.00 | 1.25 | 1.00 | 0.00 | 0.50 | 0.00 | 2.75 |
| 12 |  |  | 0.40 | 1.75 | 2.00 | 1.33 | 0.75 | 3.33 | 9.57 |
| 13 |  |  | 0.00 | 2.00 | 1.67 | 0.00 | 0.00 | 1.00 | 4.67 |
| **Mean** |  | | **0.2** | **1.8** | **1.2** | **0.5** | **0.4** | **1.3** | **5.2** |
| **SD** |  |  | **0.2** | **0.7** | **0.7** | **0.4** | **0.5** | **0.8** | **2.1** |

| **Participant** | **Training Block** | **Day** | **Depressed mood** | **Vigour** | **Physical signs and symptoms** | **Sleep disturbances** | **Perceived stress** | **Fatigue** | **Total mood disturbance** |
| --- | --- | --- | --- | --- | --- | --- | --- | --- | --- |
|  | **Recovery 2** | **40** |  | | | | | | |
| 1 |  |  | 0.00 | 1.00 | 1.67 | 1.33 | 0.00 | 1.33 | 5.33 |
| 2 |  |  | 0.00 | 1.25 | 0.00 | 0.67 | 0.00 | 0.33 | 2.25 |
| 3 |  |  | 0.00 | 1.00 | 0.67 | 0.00 | 0.25 | 1.33 | 3.25 |
| 4 |  |  | 1.40 | 2.75 | 0.33 | 2.00 | 2.00 | 2.00 | 10.48 |
| 5 |  |  | 0.00 | 1.75 | 1.00 | 0.00 | 0.00 | 0.33 | 3.08 |
| 6 |  |  | 2.40 | 3.75 | 0.00 | 1.67 | 1.50 | 2.67 | 11.98 |
| 7 |  |  | 0.60 | 2.00 | 0.67 | 1.00 | 1.50 | 1.00 | 6.77 |
| 8 |  |  | 0.00 | 1.00 | 0.67 | 0.00 | 0.00 | 0.67 | 2.33 |
| 9 |  |  | 0.00 | 1.00 | 0.67 | 0.33 | 0.00 | 0.67 | 2.67 |
| 10 |  |  | 0.60 | 1.25 | 0.33 | 0.00 | 0.50 | 1.00 | 3.68 |
| 11 |  |  | 0.00 | 1.25 | 0.00 | 3.00 | 1.50 | 0.67 | 6.42 |
| 12 |  |  | 1.20 | 3.00 | 1.33 | 1.67 | 0.50 | 3.67 | 11.37 |
| 13 |  |  | 0.00 | 2.00 | 1.67 | 0.00 | 0.00 | 1.00 | 4.67 |
| **Mean** |  | | **0.5** | **1.8** | **0.7** | **0.9** | **0.6** | **1.3** | **5.7** |
| **SD** |  |  | **0.8** | **0.9** | **0.6** | **1.0** | **0.7** | **1.0** | **3.5** |
