# Supplementary material for: The effects of intensified training on resting metabolic rate (RMR), body composition and performance in trained cyclists
Source: PLoS One. 2018 Feb 14;13(2):e0191644. doi: 10.1371/journal.pone.0191644 (PMC5812577; doi:10.1371/journal.pone.0191644)
Supplement: S18 Table — Data are presented as individual values for each time point, and group mean ± SD. (DOCX) [file pone.0191644.s019.docx]

**S18 Table:**

|  | **RESTQ-52 Sport** | | | | | | | |
| --- | --- | --- | --- | --- | --- | --- | --- | --- |
| **Participant** | **Training Block** | **Day** | **Overall Stress** | **Overall Recovery** | **Sport-specific stress** | **Sport-specific recovery** | **Total stress** | **Total recovery** |
|  | **Baseline** | **1** |  |  |  |  |  |  |
| 1 |  |  | 1.7 | 7.6 | 2.7 | 18.0 | 4.4 | 25.6 |
| 2 |  |  | 1.7 | 6.2 | 3.0 | 16.0 | 4.7 | 22.2 |
| 3 |  |  | 2.0 | 7.8 | 5.7 | 15.5 | 7.7 | 23.3 |
| 4 |  |  | 3.3 | 7.6 | 6.7 | 16.8 | 10.0 | 24.4 |
| 5 |  |  | 1.7 | 7.0 | 7.3 | 14.8 | 9.0 | 21.8 |
| 6 |  |  | 3.7 | 3.8 | 5.7 | 9.3 | 9.4 | 13.1 |
| 7 |  |  | 3.0 | 5.0 | 5.7 | 10.5 | 8.7 | 15.5 |
| 8 |  |  | 1.7 | 7.0 | 3.0 | 12.5 | 4.7 | 19.5 |
| 9 |  |  | 4.7 | 5.0 | 0.7 | 12.5 | 5.4 | 17.5 |
| 10 |  |  | 4.7 | 7.0 | 3.7 | 14.5 | 8.4 | 21.5 |
| 11 |  |  | 2.4 | 5.2 | 8.7 | 13.0 | 11.1 | 18.2 |
| 12 |  |  | 2.3 | 8.6 | 7.7 | 16.5 | 10.0 | 25.1 |
| 13 |  |  | 4.9 | 4.6 | 11.7 | 8.8 | 16.5 | 13.4 |
| **Mean** |  | | **2.9** | **6.3** | **5.5** | **13.7** | **8.5** | **20.1** |
| **SD** |  |  | **1.2** | **1.5** | **3.0** | **2.9** | **3.3** | **4.3** |

| **Participant** | **Training Block** | **Day** | **Overall Stress** | **Overall Recovery** | **Sport-specific stress** | **Sport-specific recovery** | **Total stress** | **Total recovery** |
| --- | --- | --- | --- | --- | --- | --- | --- | --- |
|  | **Baseline** | **5** |  |  |  |  |  |  |
| 1 |  |  | 1.0 | 8.6 | 2.7 | 22.0 | 3.7 | 30.6 |
| 2 |  |  | 1.3 | 8.0 | 3.0 | 18.0 | 4.3 | 26.0 |
| 3 |  |  | 2.1 | 7.8 | 4.3 | 14.3 | 6.5 | 22.1 |
| 4 |  |  | 3.3 | 7.8 | 9.0 | 16.3 | 12.3 | 24.1 |
| 5 |  |  | 2.4 | 7.2 | 5.7 | 16.3 | 8.1 | 23.5 |
| 6 |  |  | 2.1 | 4.4 | 5.3 | 9.5 | 7.5 | 13.9 |
| 7 |  |  | 2.7 | 3.4 | 4.0 | 10.8 | 6.7 | 14.2 |
| 8 |  |  | 1.0 | 6.4 | 2.7 | 17.5 | 3.7 | 23.9 |
| 9 |  |  | 2.6 | 4.4 | 6.0 | 12.3 | 8.6 | 16.7 |
| 10 |  |  | 2.9 | 5.8 | 5.0 | 8.3 | 7.9 | 14.1 |
| 11 |  |  | 2.4 | 7.2 | 7.0 | 14.5 | 9.4 | 21.7 |
| 12 |  |  | 3.4 | 7.2 | 13.3 | 8.3 | 16.8 | 15.5 |
| 13 |  |  | 2.9 | 7.8 | 7.7 | 16.5 | 10.5 | 24.3 |
| **Mean** |  | | **2.3** | **6.6** | **5.8** | **14.2** | **8.1** | **20.8** |
| **SD** |  |  | **0.8** | **1.6** | **3.0** | **4.2** | **3.6** | **5.4** |

| **Participant** | **Training Block** | **Day** | **Overall Stress** | **Overall Recovery** | **Sport-specific stress** | **Sport-specific recovery** | **Total stress** | **Total recovery** |
| --- | --- | --- | --- | --- | --- | --- | --- | --- |
|  | **Build** | **9** |  |  |  |  |  |  |
| 1 |  |  | 1.3 | 9.8 | 0.7 | 20.8 | 2.0 | 30.6 |
| 2 |  |  | 1.3 | 8.2 | 3.0 | 16.3 | 4.3 | 24.5 |
| 3 |  |  | 2.0 | 7.8 | 4.3 | 14.5 | 6.3 | 22.3 |
| 4 |  |  | 3.3 | 7.0 | 10.7 | 9.0 | 14.0 | 16.0 |
| 5 |  |  | 1.6 | 7.4 | 5.0 | 15.0 | 6.6 | 22.4 |
| 6 |  |  | 2.3 | 4.0 | 3.0 | 10.0 | 5.3 | 14.0 |
| 7 |  |  | 1.9 | 5.4 | 7.0 | 9.0 | 8.9 | 14.4 |
| 8 |  |  | 1.3 | 7.8 | 1.7 | 17.5 | 3.0 | 25.3 |
| 9 |  |  | 2.7 | 4.6 | 3.7 | 10.8 | 6.4 | 15.4 |
| 10 |  |  | 2.4 | 4.4 | 6.3 | 6.8 | 8.8 | 11.2 |
| 11 |  |  | 3.4 | 7.2 | 6.7 | 18.0 | 10.1 | 25.2 |
| 12 |  |  | 2.6 | 5.8 | 12.7 | 9.8 | 15.2 | 15.6 |
| 13 |  |  | 3.0 | 8.2 | 7.7 | 17.5 | 10.7 | 25.7 |
| **Mean** |  | | **2.2** | **6.7** | **5.6** | **13.4** | **7.8** | **20.2** |
| **SD** |  |  | **0.8** | **1.8** | **3.4** | **4.4** | **4.0** | **6.0** |

| **Participant** | **Training Block** | **Day** | **Overall Stress** | **Overall Recovery** | **Sport-specific stress** | **Sport-specific recovery** | **Total stress** | **Total recovery** |
| --- | --- | --- | --- | --- | --- | --- | --- | --- |
|  | **Baseline** | **12** |  |  |  |  |  |  |
| 1 |  |  | 1.6 | 8.4 | 3.3 | 22.3 | 4.9 | 30.7 |
| 2 |  |  | 1.7 | 7.8 | 3.3 | 16.5 | 5.0 | 24.3 |
| 3 |  |  | 1.6 | 8.2 | 7.0 | 14.8 | 8.6 | 23.0 |
| 4 |  |  | 3.0 | 5.8 | 7.0 | 11.5 | 10.0 | 17.3 |
| 5 |  |  | 1.7 | 6.6 | 6.3 | 13.0 | 8.0 | 19.6 |
| 6 |  |  | 1.9 | 4.6 | 4.3 | 9.5 | 6.2 | 14.1 |
| 7 |  |  | 3.6 | 4.2 | 8.3 | 9.5 | 11.9 | 13.7 |
| 8 |  |  | 1.7 | 6.0 | 3.7 | 12.5 | 5.4 | 18.5 |
| 9 |  |  | 3.6 | 4.8 | 7.7 | 13.3 | 11.2 | 18.1 |
| 10 |  |  | 4.3 | 4.4 | 5.0 | 8.8 | 9.3 | 13.2 |
| 11 |  |  | 2.6 | 5.8 | 7.0 | 15.3 | 9.6 | 21.1 |
| 12 |  |  | 3.9 | 5.6 | 12.0 | 9.5 | 15.9 | 15.1 |
| 13 |  |  | 2.7 | 8.4 | 7.7 | 18.3 | 10.4 | 26.7 |
| **Mean** |  | | **2.6** | **6.2** | **6.4** | **13.4** | **9.0** | **19.6** |
| **SD** |  |  | **1.0** | **1.6** | **2.4** | **4.0** | **3.1** | **5.3** |

| **Participant** | **Training Block** | **Day** | **Overall Stress** | **Overall Recovery** | **Sport-specific stress** | **Sport-specific recovery** | **Total stress** | **Total recovery** |
| --- | --- | --- | --- | --- | --- | --- | --- | --- |
|  | **Loading 1** | **15** |  |  |  |  |  |  |
| 1 |  |  | 1.1 | 8.4 | 6.7 | 20.8 | 7.8 | 29.2 |
| 2 |  |  | 1.3 | 7.8 | 3.3 | 16.5 | 4.6 | 24.3 |
| 3 |  |  | 1.6 | 8.2 | 4.7 | 15.5 | 6.2 | 23.7 |
| 4 |  |  | 3.4 | 5.8 | 9.3 | 12.8 | 12.8 | 18.6 |
| 5 |  |  | 1.7 | 6.2 | 5.7 | 14.3 | 7.4 | 20.5 |
| 6 |  |  | 2.1 | 3.6 | 5.7 | 9.8 | 7.8 | 13.4 |
| 7 |  |  | 2.4 | 4.6 | 8.7 | 9.3 | 11.1 | 13.9 |
| 8 |  |  | 1.9 | 5.8 | 3.7 | 10.8 | 5.5 | 16.6 |
| 9 |  |  | 3.7 | 4.2 | 6.7 | 12.0 | 10.4 | 16.2 |
| 10 |  |  | 4.4 | 5.4 | 4.0 | 8.0 | 8.4 | 13.4 |
| 11 |  |  | 2.3 | 7.2 | 8.7 | 17.0 | 11.0 | 24.2 |
| 12 |  |  | 3.1 | 4.6 | 14.0 | 7.5 | 17.1 | 12.1 |
| 13 |  |  | 2.9 | 8.6 | 8.0 | 18.0 | 10.9 | 26.6 |
| **Mean** |  | | **2.5** | **6.2** | **6.8** | **13.2** | **9.3** | **19.4** |
| **SD** |  |  | **1.0** | **1.7** | **2.9** | **4.1** | **3.4** | **5.7** |

| **Participant** | **Training Block** | **Day** | **Overall Stress** | **Overall Recovery** | **Sport-specific stress** | **Sport-specific recovery** | **Total stress** | **Total recovery** |
| --- | --- | --- | --- | --- | --- | --- | --- | --- |
|  | **Loading 1** | **17** |  |  |  |  |  |  |
| 1 |  |  | 1.7 | 8.8 | 5.3 | 23.3 | 7.0 | 32.1 |
| 2 |  |  | 2.0 | 7.8 | 3.7 | 16.8 | 5.7 | 24.6 |
| 3 |  |  | 1.7 | 8.6 | 5.7 | 16.0 | 7.4 | 24.6 |
| 4 |  |  | 3.4 | 5.8 | 11.0 | 13.8 | 14.4 | 19.6 |
| 5 |  |  | 2.7 | 4.6 | 7.7 | 11.0 | 10.4 | 15.6 |
| 6 |  |  | 1.7 | 3.8 | 4.3 | 9.5 | 6.0 | 13.3 |
| 7 |  |  | 3.6 | 4.4 | 9.3 | 9.3 | 12.9 | 13.7 |
| 8 |  |  | 1.7 | 5.6 | 4.7 | 8.3 | 6.4 | 13.9 |
| 9 |  |  | 3.4 | 4.8 | 8.3 | 13.8 | 11.8 | 18.6 |
| 10 |  |  | 4.4 | 4.8 | 5.3 | 6.5 | 9.8 | 11.3 |
| 11 |  |  | 2.6 | 6.2 | 9.3 | 16.0 | 11.9 | 22.2 |
| 12 |  |  | 4.6 | 4.2 | 10.7 | 6.5 | 15.2 | 10.7 |
| 13 |  |  | 2.7 | 7.6 | 7.7 | 17.0 | 10.4 | 24.6 |
| **Mean** |  | | **2.8** | **5.9** | **7.2** | **12.9** | **9.9** | **18.8** |
| **SD** |  |  | **1.0** | **1.7** | **2.5** | **4.9** | **3.2** | **6.5** |

| **Participant** | **Training Block** | **Day** | **Overall Stress** | **Overall Recovery** | **Sport-specific stress** | **Sport-specific recovery** | **Total stress** | **Total recovery** |
| --- | --- | --- | --- | --- | --- | --- | --- | --- |
|  | **Loading 1** | **19** |  |  |  |  |  |  |
| 1 |  |  | 1.1 | 10.0 | 5.7 | 23.3 | 6.8 | 33.3 |
| 2 |  |  | 2.0 | 6.8 | 3.0 | 14.8 | 5.0 | 21.6 |
| 3 |  |  | 1.7 | 9.0 | 7.3 | 15.8 | 9.0 | 24.8 |
| 4 |  |  | 3.9 | 6.0 | 10.7 | 11.3 | 14.5 | 17.3 |
| 5 |  |  | 1.9 | 5.4 | 8.7 | 10.5 | 10.5 | 15.9 |
| 6 |  |  | 2.7 | 4.2 | 10.7 | 9.0 | 13.4 | 13.2 |
| 7 |  |  | 4.4 | 3.4 | 10.3 | 8.3 | 14.8 | 11.7 |
| 8 |  |  | 1.7 | 6.2 | 4.7 | 8.5 | 6.4 | 14.7 |
| 9 |  |  | 3.6 | 4.4 | 7.0 | 13.0 | 10.6 | 17.4 |
| 10 |  |  | 4.3 | 4.2 | 5.3 | 7.8 | 9.6 | 12.0 |
| 11 |  |  | 2.1 | 6.6 | 8.3 | 16.5 | 10.5 | 23.1 |
| 12 |  |  | 4.3 | 5.2 | 10.0 | 7.3 | 14.3 | 12.5 |
| 13 |  |  | 3.0 | 7.4 | 8.0 | 16.3 | 11.0 | 23.7 |
| **Mean** |  | | **2.8** | **6.1** | **7.7** | **12.5** | **10.5** | **18.5** |
| **SD** |  |  | **1.2** | **1.9** | **2.5** | **4.7** | **3.2** | **6.4** |

| **Participant** | **Training Block** | **Day** | **Overall Stress** | **Overall Recovery** | **Sport-specific stress** | **Sport-specific recovery** | **Total stress** | **Total recovery** |
| --- | --- | --- | --- | --- | --- | --- | --- | --- |
|  | **Loading 2** | **22** |  |  |  |  |  |  |
| 1 |  |  | 1.0 | 9.8 | 5.3 | 23.8 | 6.3 | 33.6 |
| 2 |  |  | 2.1 | 6.2 | 4.0 | 16.0 | 6.1 | 22.2 |
| 3 |  |  | 1.7 | 8.0 | 7.0 | 18.5 | 8.7 | 26.5 |
| 4 |  |  | 3.4 | 6.6 | 11.7 | 14.8 | 15.1 | 21.4 |
| 5 |  |  | 1.9 | 6.8 | 8.0 | 14.8 | 9.9 | 21.6 |
| 6 |  |  | 4.4 | 3.4 | 10.0 | 8.0 | 14.4 | 11.4 |
| 7 |  |  | 3.7 | 4.0 | 9.7 | 7.0 | 13.4 | 11.0 |
| 8 |  |  | 1.6 | 7.6 | 7.0 | 6.3 | 8.6 | 13.9 |
| 9 |  |  | 2.6 | 5.6 | 9.0 | 9.0 | 11.6 | 14.6 |
| 10 |  |  | 4.4 | 3.0 | 6.7 | 5.0 | 11.1 | 8.0 |
| 11 |  |  | 2.9 | 5.2 | 12.0 | 15.3 | 14.9 | 20.5 |
| 12 |  |  | 3.7 | 5.8 | 10.0 | 11.3 | 13.7 | 17.1 |
| 13 |  |  | 2.6 | 7.8 | 8.3 | 16.5 | 10.9 | 24.3 |
| **Mean** |  | | **2.8** | **6.1** | **8.4** | **12.8** | **11.1** | **18.9** |
| **SD** |  |  | **1.1** | **2.0** | **2.4** | **5.5** | **3.1** | **7.1** |

| **Participant** | **Training Block** | **Day** | **Overall Stress** | **Overall Recovery** | **Sport-specific stress** | **Sport-specific recovery** | **Total stress** | **Total recovery** |
| --- | --- | --- | --- | --- | --- | --- | --- | --- |
|  | **Loading 2** | **24** |  |  |  |  |  |  |
| 1 |  |  | 1.3 | 10.0 | 6.0 | 22.3 | 7.3 | 32.3 |
| 2 |  |  | 1.7 | 6.6 | 4.0 | 15.0 | 5.7 | 21.6 |
| 3 |  |  | 2.3 | 7.6 | 12.0 | 13.0 | 14.3 | 20.6 |
| 4 |  |  | 3.9 | 6.2 | 12.3 | 10.0 | 16.2 | 16.2 |
| 5 |  |  | 3.7 | 4.2 | 9.7 | 10.0 | 13.4 | 14.2 |
| 6 |  |  | 3.7 | 3.8 | 10.0 | 7.5 | 13.7 | 11.3 |
| 7 |  |  | 2.9 | 3.6 | 7.7 | 7.5 | 10.5 | 11.1 |
| 8 |  |  | 1.6 | 6.0 | 5.3 | 8.5 | 6.9 | 14.5 |
| 9 |  |  | 3.4 | 5.2 | 10.7 | 4.5 | 14.1 | 9.7 |
| 10 |  |  | 4.6 | 3.8 | 7.0 | 5.5 | 11.6 | 9.3 |
| 11 |  |  | 4.0 | 5.6 | 13.0 | 14.3 | 17.0 | 19.9 |
| 12 |  |  | 4.3 | 5.0 | 14.3 | 8.8 | 18.6 | 13.8 |
| 13 |  |  | 2.9 | 8.2 | 8.0 | 16.5 | 10.9 | 24.7 |
| **Mean** |  | | **3.1** | **5.8** | **9.2** | **11.0** | **12.3** | **16.9** |
| **SD** |  |  | **1.1** | **1.9** | **3.2** | **5.0** | **4.0** | **6.7** |

| **Participant** | **Training Block** | **Day** | **Overall Stress** | **Overall Recovery** | **Sport-specific stress** | **Sport-specific recovery** | **Total stress** | **Total recovery** |
| --- | --- | --- | --- | --- | --- | --- | --- | --- |
|  | **Loading 2** | **26** |  |  |  |  |  |  |
| 1 |  |  | 1.7 | 10.0 | 11.3 | 21.8 | 13.0 | 31.8 |
| 2 |  |  | 2.4 | 6.2 | 5.7 | 13.0 | 8.1 | 19.2 |
| 3 |  |  | 3.7 | 7.4 | 13.7 | 13.8 | 17.4 | 21.2 |
| 4 |  |  | 3.9 | 5.6 | 12.3 | 10.3 | 16.2 | 15.9 |
| 5 |  |  | 2.4 | 5.6 | 8.0 | 10.8 | 10.4 | 16.4 |
| 6 |  |  | 1.9 | 4.4 | 5.3 | 9.5 | 7.2 | 13.9 |
| 7 |  |  | 3.3 | 4.0 | 8.7 | 7.5 | 12.0 | 11.5 |
| 8 |  |  | 2.0 | 6.2 | 7.3 | 9.5 | 9.3 | 15.7 |
| 9 |  |  | 3.1 | 4.4 | 9.0 | 9.0 | 12.1 | 13.4 |
| 10 |  |  | 5.0 | 4.2 | 9.0 | 7.0 | 14.0 | 11.2 |
| 11 |  |  | 2.7 | 6.2 | 12.7 | 15.3 | 15.4 | 21.5 |
| 12 |  |  | 8.0 | 3.4 | 19.3 | 3.8 | 27.3 | 7.2 |
| 13 |  |  | 3.1 | 8.0 | 8.0 | 16.8 | 11.1 | 24.8 |
| **Mean** |  | | **3.3** | **5.8** | **10.0** | **11.4** | **13.4** | **17.2** |
| **SD** |  |  | **1.7** | **1.9** | **3.8** | **4.7** | **5.2** | **6.5** |

| **Participant** | **Training Block** | **Day** | **Overall Stress** | **Overall Recovery** | **Sport-specific stress** | **Sport-specific recovery** | **Total stress** | **Total recovery** |
| --- | --- | --- | --- | --- | --- | --- | --- | --- |
|  | **Loading 2** | **29** |  |  |  |  |  |  |
| 1 |  |  | 1.1 | 7.2 | 8.3 | 21.3 | 9.5 | 28.5 |
| 2 |  |  | 3.0 | 5.6 | 6.0 | 12.3 | 9.0 | 17.9 |
| 3 |  |  | 5.7 | 5.8 | 17.0 | 9.3 | 22.7 | 15.1 |
| 4 |  |  | 4.6 | 6.6 | 16.0 | 9.8 | 20.6 | 16.4 |
| 5 |  |  | 2.7 | 5.4 | 9.3 | 12.0 | 12.0 | 17.4 |
| 6 |  |  | 2.4 | 4.2 | 6.0 | 9.8 | 8.4 | 14.0 |
| 7 |  |  | 3.1 | 3.4 | 7.3 | 8.8 | 10.5 | 12.2 |
| 8 |  |  | 1.6 | 7.0 | 3.7 | 11.3 | 5.2 | 18.3 |
| 9 |  |  | 2.9 | 5.0 | 11.0 | 10.0 | 13.9 | 15.0 |
| 10 |  |  | 4.4 | 5.2 | 12.3 | 5.8 | 16.8 | 11.0 |
| 11 |  |  | 2.3 | 5.4 | 11.0 | 13.3 | 13.3 | 18.7 |
| 12 |  |  | 5.0 | 4.4 | 16.0 | 5.8 | 21.0 | 10.2 |
| 13 |  |  | 2.7 | 8.2 | 7.7 | 16.0 | 10.4 | 24.2 |
| **Mean** |  | | **3.2** | **5.6** | **10.1** | **11.2** | **13.3** | **16.8** |
| **SD** |  |  | **1.4** | **1.3** | **4.2** | **4.1** | **5.4** | **5.1** |

| **Participant** | **Training Block** | **Day** | **Overall Stress** | **Overall Recovery** | **Sport-specific stress** | **Sport-specific recovery** | **Total stress** | **Total recovery** |
| --- | --- | --- | --- | --- | --- | --- | --- | --- |
|  | **Recovery 1** | **33** |  |  |  |  |  |  |
| 1 |  |  | 1.3 | 8.2 | 5.7 | 23.0 | 7.0 | 31.2 |
| 2 |  |  | 2.0 | 6.2 | 3.7 | 13.5 | 5.7 | 19.7 |
| 3 |  |  | 2.9 | 7.6 | 7.3 | 14.5 | 10.2 | 22.1 |
| 4 |  |  | 3.9 | 5.4 | 7.3 | 11.8 | 11.2 | 17.2 |
| 5 |  |  | 1.1 | 7.2 | 5.3 | 13.3 | 6.5 | 20.5 |
| 6 |  |  | 3.7 | 3.2 | 7.7 | 7.0 | 11.4 | 10.2 |
| 7 |  |  | 3.4 | 4.4 | 7.3 | 8.5 | 10.8 | 12.9 |
| 8 |  |  | 1.7 | 7.4 | 2.0 | 15.5 | 3.7 | 22.9 |
| 9 |  |  | 2.0 | 5.8 | 4.7 | 14.8 | 6.7 | 20.6 |
| 10 |  |  | 4.0 | 4.0 | 8.0 | 4.0 | 12.0 | 8.0 |
| 11 |  |  | 2.0 | 7.4 | 3.7 | 14.8 | 5.7 | 22.2 |
| 12 |  |  | 3.1 | 5.6 | 6.0 | 9.5 | 9.1 | 15.1 |
| 13 |  |  | 2.7 | 7.6 | 8.0 | 15.8 | 10.7 | 23.4 |
| **Mean** |  | | **2.6** | **6.2** | **5.9** | **12.8** | **8.5** | **18.9** |
| **SD** |  |  | **1.0** | **1.6** | **1.9** | **4.8** | **2.7** | **6.2** |

| **Participant** | **Training Block** | **Day** | **Overall Stress** | **Overall Recovery** | **Sport-specific stress** | **Sport-specific recovery** | **Total stress** | **Total recovery** |
| --- | --- | --- | --- | --- | --- | --- | --- | --- |
|  | **Recovery 1** | **36** |  |  |  |  |  |  |
| 1 |  |  | 1.4 | 9.2 | 4.0 | 23.3 | 5.4 | 32.5 |
| 2 |  |  | 1.6 | 7.8 | 2.3 | 18.0 | 3.9 | 25.8 |
| 3 |  |  | 2.4 | 8.6 | 10.0 | 15.8 | 12.4 | 24.4 |
| 4 |  |  | 3.0 | 7.4 | 2.7 | 13.3 | 5.7 | 20.7 |
| 5 |  |  | 1.4 | 8.2 | 4.0 | 12.8 | 5.4 | 21.0 |
| 6 |  |  | 4.3 | 3.2 | 6.7 | 5.3 | 11.0 | 8.5 |
| 7 |  |  | 2.7 | 4.2 | 6.7 | 8.8 | 9.4 | 13.0 |
| 8 |  |  | 1.3 | 7.4 | 2.3 | 18.0 | 3.6 | 25.4 |
| 9 |  |  | 1.9 | 5.8 | 5.3 | 15.0 | 7.2 | 20.8 |
| 10 |  |  | 3.1 | 5.6 | 11.0 | 4.8 | 14.1 | 10.4 |
| 11 |  |  | 2.7 | 7.4 | 6.7 | 17.5 | 9.4 | 24.9 |
| 12 |  |  | 3.1 | 5.8 | 7.3 | 9.8 | 10.5 | 15.6 |
| 13 |  |  | 2.6 | 7.8 | 8.0 | 15.0 | 10.6 | 22.8 |
| **Mean** |  | | **2.4** | **6.8** | **5.9** | **13.6** | **8.4** | **20.4** |
| **SD** |  |  | **0.9** | **1.8** | **2.8** | **5.3** | **3.4** | **6.9** |

| **Participant** | **Training Block** | **Day** | **Overall Stress** | **Overall Recovery** | **Sport-specific stress** | **Sport-specific recovery** | **Total stress** | **Total recovery** |
| --- | --- | --- | --- | --- | --- | --- | --- | --- |
|  | **Recovery 2** | **40** |  |  |  |  |  |  |
| 1 |  |  | 2.6 | 10.0 | 5.0 | 22.3 | 7.6 | 32.3 |
| 2 |  |  | 1.3 | 8.2 | 2.3 | 17.8 | 3.6 | 26.0 |
| 3 |  |  | 2.3 | 8.0 | 6.3 | 16.3 | 8.6 | 24.3 |
| 4 |  |  | 5.1 | 5.6 | 4.3 | 5.3 | 9.5 | 10.9 |
| 5 |  |  | 1.3 | 7.6 | 4.3 | 13.3 | 5.6 | 20.9 |
| 6 |  |  | 5.9 | 3.8 | 5.3 | 2.5 | 11.2 | 6.3 |
| 7 |  |  | 3.3 | 4.8 | 5.0 | 9.0 | 8.3 | 13.8 |
| 8 |  |  | 1.0 | 7.2 | 2.3 | 17.3 | 3.3 | 24.5 |
| 9 |  |  | 1.7 | 5.8 | 5.0 | 13.3 | 6.7 | 19.1 |
| 10 |  |  | 3.0 | 4.4 | 4.7 | 5.5 | 7.7 | 9.9 |
| 11 |  |  | 3.0 | 4.4 | 4.7 | 5.5 | 7.7 | 9.9 |
| 12 |  |  | 3.1 | 4.8 | 3.7 | 13.3 | 6.8 | 18.1 |
| 13 |  |  | 3.7 | 5.6 | 5.0 | 6.5 | 8.7 | 12.1 |
| **Mean** |  | | **2.9** | **6.2** | **4.5** | **11.3** | **7.3** | **17.5** |
| **SD** |  |  | **1.5** | **1.9** | **1.1** | **6.1** | **2.2** | **7.8** |
